# MVSE: An R‐package that estimates a climate‐driven mosquito‐borne viral suitability index

**DOI:** 10.1111/2041-210X.13205

**Published:** 2019-06-19

**Authors:** Uri Obolski, Pablo N. Perez, Christian J. Villabona‐Arenas, Julien Thézé, Nuno R. Faria, José Lourenço

**Affiliations:** ^1^ School of Public Health Tel Aviv University Tel Aviv Israel; ^2^ Porter School of the Environment and Earth Sciences Tel Aviv University Tel Aviv Israel; ^3^ Department of Infectious Disease Epidemiology Imperial College London London UK; ^4^ Centre for Mathematical Modelling of Infectious Diseases Department of Infectious Disease Epidemiology Faculty of Epidemiology and Population Health, London School of Hygiene and Tropical Medicine London UK; ^5^ Department of Zoology University of Oxford Oxford UK

**Keywords:** community ecology, community ecological modelling, disease ecology, disease ecological modelling, microbial ecology, mosquitoes, mosquito-borne Viral Suitability Estimator, viruses

## Abstract

1. Viruses, such as dengue, Zika, yellow fever and chikungunya, depend on mosquitoes for transmission. Their epidemics typically present periodic patterns, linked to the underlying mosquito population dynamics, which are known to be driven by natural climate fluctuations. Understanding how climate dictates the timing and potential of viral transmission is essential for preparedness of public health systems and design of control strategies. While various alternative approaches have been proposed to estimate local transmission potential of such viruses, few open‐source, ready to use and freely available software tools exist.
2. We developed the **M**osquito‐borne **V**iral **S**uitability **E**stimator (MVSE) software package for the R programming environment. MVSE estimates the index P, a novel suitability index based on a climate‐driven mathematical expression for the basic reproductive number of mosquito‐borne viruses. By accounting for local humidity and temperature, as well as viral, vector and human priors, the index P can be estimated for specific host and viral species in different regions of the globe.
3. We describe the background theory, empirical support and biological interpretation of the index P. Using real‐world examples spanning multiple epidemiological contexts, we further demonstrate MVSE's basic functionality, research and educational potentials.

## Abstract (pt)

Introdução: Os vírus da dengue, Zika, febre amarela e chikungunya mantém‐se em ciclos de transmissão entre humanos e mosquitos. As epidemias desses vírus apresentam padrões oscilatórios periódicos de carácter universal, que refletem flutuações no número total de mosquitos presentes, por sua vez influenciadas por flutuações climáticas naturais que afetam o ciclo de vida do mosquito. O conhecimento de como o clima resulta em janelas de oportunidade favorável à transmissão desses vírus é essencial para o planeamento de ações de saúde pública, como campanhas de prevenção, alocação de recursos em sistemas de saúde e ações de controlo do mosquito. Enquanto que vários métodos que estimam o potencial desses vírus segundo dados climáticos tȇm sido publicados em anos recentes, ferramentas de software grátis e open‐source são praticamente inexistentes. Resultados: Nós desenvolvemos o Mosquito‐borne Viral Suitability Estimator (MVSE), um pacote de software para o sistema de programação R. MVSE estima o index P, uma nova medida informada por dados climáticos, baseada na equação do número reprodutivo básico (R0) de um modelo dinâmico. A expressão do R0 usada considera informação a priori para parâmetros humanos, virais e entomológicos, assim como séries temporais de temperatura e humidade. Assim sendo, o index P pode ser calculado para qualquer hospedeiro e vírus de interesse em qualquer região do globo para qual dados climáticos estão disponíveis. Conclusões: Neste manuscrito, a teoria, o suporte empírico e a interpretação biológica do novo index P são introduzidas e discutidas. Usando exemplos reais em múltiplos contextos epidemiológicos da América do Sul e Central, nós demonstramos também as funcionalidades básicas do MVSE e o seu potencial para fins académicos e educacionais.

## 1 INTRODUCTION

Common mosquito‐borne viruses include the dengue (DENV), chikungunya (CHIKV), Zika (ZIKV), yellow fever (YFV), Rift Valley fever (RVFV), West Nile (WNV) and Japanese encephalitis (JEV) viruses. Due to ongoing human and climatic trends that favour the establishment of mosquitoes and movement of infectious hosts, these pathogens are becoming increasingly detrimental for human health and local economies (Jaenisch et al., 2014; Schwarz et al., 2012; Stanaway et al., 2016). For instance, in the last 5 years, Europe has witnessed its first DENV and CHIKV sustained outbreaks (Guzzetta et al., 2017; Lourenço et al., 2018). At the same time, ZIKV and CHIKV have recently taken a toll on the populations of island nations (Cauchemez et al., 2016; Duffy et al., 2009), South America and the Caribbean (Faria et al., 2016a,2016b, 2017b; Louren¸co et al., 2017). In Africa, YFV outbreaks in Angola and other countries have recently (2016–2017) developed into a global vaccination crisis (Kraemer et al., 2016; Wu, Peak, Leung, & Lipsitch, 2016). More recently (2017–2018), YFV has also emerged from its sylvatic cycle in Brazil (Faria et al., 2018). Not only is the absolute scale of these outbreaks unprecedented, but the emergence of severe pathologies such as ZIKV‐related neonatal microcephaly, CHIKV‐induced Guillain–Barré syndrome and a very high YFV mortality rate have had substantial negative public health and socio‐economic impacts (Cao‐Lormeau et al., 2016; 16; Johansson, Mier‐y Teran‐Romero, Reefhuis, Gilboa, & Hills, 2016; Oehler et al., 2015; de Oliveira et al., 2017).

The evolutionary and host–pathogen history of mosquito‐borne viruses is vastly diverse. For instance, while a strong ecological barrier separates human and zoonotic variants of DENV and ZIKV (Holmes & Twiddy, 2003; Lessler et al., 2016), such a barrier is less evident for YFV, WNV and JEV (Lanciotti et al., 1999; Rosen, 1986). DENV also uniquely presents four antigenically distinct lineages (serotypes DENV1–4) with complex immunological responses (Flasche et al., 2016; 22; Lourenço et al., 2018; Lourenço & Recker, 2016), whereas WNV has the vastest host tropism including primates, equines, birds and reptiles (Mackenzie, Gubler, & Petersen, 2004; Rosen, 1986). Importantly, however, the population biology of these viruses shares one unifying characteristic: the dynamics of their epidemic behaviour are inherently linked to the underlying population dynamics of their vector species. Mosquito‐population dynamics are known to be dictated by a wide range of factors, such as climate, altitude, population density of humans or other mammals, pollution levels and natural or artificial water reservoirs (Chen et al., 2012; Lozano‐Fuentes et al., 2012; Monteiro, de Souza, & de Albuquerque, 2007; Williams, Bader, Kearney, Ritchie, & Russell, 2010). While most of these factors can dictate absolute population sizes (carrying capacity), seasonal oscillations in size are directly and indirectly driven by natural climate variations (Brady et al., 2013; Dutta, Khan, Khan, Sharma, & Mahanta, 2010; Honório, Codeço, Alves, Magalhaes, & Lourenço‐De‐Oliveira, 2009; LaBeaud, Bashir, & King, 2011; Madeira, Macharelli, & Carvalho, 2002; Mohammed & Chadee, 2011; Oo, Storch, Madon, & Becker, 2011; Yasuno & Tonn, 1960).

Climatic variables act on mosquito metabolic processes, triggering individual‐level physical and behavioural (trait) changes which translate into population‐level dynamic changes. Controlled laboratory experiments have been able to measure such individual‐level effects on mosquito traits. For instance, temperature has been shown to affect adult mortality (Brady et al., 2013; Yang, Macoris, Galvani, Andrighetti, & Wanderley, 2009), aquatic phase mortality (Yang et al., 2009), pathogen extrinsic incubation period (Focks, Daniels, Haile, & Keesling, 1995; MacDonald, 1957; Otero, Solari, & Schweigmann, 2006; Schoolfield, Sharpe, & Magnuson, 1981; Watts, Burke, Harrison, Whitmire, & Nisalak, 1987), flight performance (Rowley & Graham, 1968), biting rate (Hamlet et al., 2018a), aquatic phase transition rate (Yang et al., 2009), probability of transmission to a host per infectious bite (Lambrechts et al., 2011; Watts et al., 1987; Xiao et al., 2014), oviposition rate (Ezeakacha, 2015; Yang et al., 2009), body size (Alto & Juliano, 2001; Ezeakacha, 2015) and so on. Humidity has also been shown to affect adult mortality (Alto & Juliano, 2001; Ezeakacha, 2015), biting rate (Yasuno & Tonn, 1960), wing length (Alto & Juliano, 2001), flight performance (Rowley & Graham, 1968), pathogen extrinsic incubation period (Thu, Aye, & Thein, 1998), egg‐hatching success (Dickerson, 2007; Ezeakacha, 2015; Russell, Kay, & Shipton, 2001), oviposition behaviour (Madeira et al., 2002) and so on. The accumulation of empirical data from such experimental studies has allowed the derivation of mosquito climate–trait relationships, for which mathematical expressions now exist (see e.g. Mordecai et al., 2017; Yang et al., 2009).

Mechanistic transmission models have long been used to research the population biology and dynamics of mosquito‐borne pathogens. In this context, transmission potential can be defined through the basic reproduction number (R0)—the expected number of new cases generated by a single infection in a completely susceptible population—of which the classic mosquito‐borne example is the Ross‐Macdonald formulation applied to malaria (MacDonald, 1957). Generally, the R0 of a mosquito‐borne pathogen involves a complex interplay between multiple host, viral and entomological factors. Since many of these are difficult to parameterize, simplifications are often implemented, and a multitude of R0‐based approaches of measuring the key components of transmission by mosquitoes exists in the literature (Smith et al., 2012). One of the most widely used is the concept of vectorial capacity (VC), which stems from solely considering R0′s entomological factors (Brady et al., 2016; Smith et al., 2012). VC, also termed the daily reproduction number, is the expected number of infective mosquito bites that would result from all potentially biting mosquitoes of a single infectious host during 1 day. VC has contributed immensely to the theory of malaria elimination programmes (Brady et al., 2016; Gething et al., 2011; Smith et al., 2012). Furthermore, VC has recently been parameterized with the aforementioned mathematical climate–trait relationships from empirical studies, and has been successfully applied to derive new transmission potential measures for emerging mosquito‐borne viruses, such as DENV (Brady et al., 2014; Liu‐Helmersson, Stenlund, Wilder‐Smith, & Rocklӧv, 2014; Mordecai et al., 2017), CHIKV (Mordecai et al., 2017), ZIKV (Messina et al., 2016; Mordecai et al., 2017) and YFV (Hamlet et al., 2018b).

We have previously developed a mechanistic climate‐driven transmission model, which incorporates mathematical formulations of climate–trait relationships into key model parameters. This model has been successfully used to study the entomological and epidemiological determinants of the 2012 DENV1 outbreak in the island of Madeira (Portugal) (Louren¸co et al., 2014), the 2014 DENV4 outbreak in Rio de Janeiro (Brazil) (Faria et al., 2017a) and the 2015–2017 ZIKV outbreak in Feira de Santana (Brazil) (Lourenço et al., 2017). In these case studies, the model was fit to notified epidemic curves using Markov chain Monte Carlo, allowing the estimation of epidemiological parameters such as R0. Following a similar rationale to previous work based on vectorial capacity measures (Brady et al., 2014; Gething et al., 2011; Hamlet et al., 2018a; Mordecai et al., 2017), we have recently shown that a mosquito‐borne viral suitability measure called the *index* P can be derived from the R0′s expression of our modelling framework (Perez‐Guzman et al., 2018). In theory, this index is a proxy for timing and scale of mosquito‐borne viral transmission potential, without the need to fit epidemic curves. In the context of Myanmar, we have recently shown that the index P is highly correlated with both city‐level mosquito infestation and dengue notifications (Perez‐Guzman et al., 2018).

In this article, we translate our experience with this modelling framework and index P into a novel R‐package named the **M**osquito‐borne **V**iral **S**uitability **E**stimator (MVSE). The main goals of MVSE are to serve as a free software tool for estimation of transmission potential, while sparing the user from the mathematical complexity of epidemiological models, free of the need for incidence time series and independent of variables and data sources that are difficult to access. We describe in detail the theoretical and empirical rationales behind the index P as well as MVSE's functionalities using real‐world examples spanning multiple epidemiological contexts.

## 2 THEORY, DESIGN AND IMPLEMENTATION

### 2.1 The suitability index P

#### 2.1.1 Index derivation and interpretation

The transmission potential of a pathogen can be summarized through the basic (R0) and effective (Re) reproduction numbers. The R0 of a pathogen is the number of secondary cases generated, on average, by a single infected host in a totally susceptible population. In the case of mosquito‐borne viruses, R0 can also be interpreted as the sum of the reproductive potential (transmission) of each adult female mosquito, $P_{\left(u,t\right)}$, over the total number of female mosquitoes per human, *M*, in a totally susceptible host population (Equation 1). The Re of a mosquito‐borne virus can be interpreted in a similar manner, but taking into consideration the presence of immune hosts hampering transmission potential (Equation 2, with $S_{h}$, $S_{v}$ the proportion of susceptible humans and mosquitoes, respectively).

(1) $${R_{0}}_{\left(u,t\right)}=\sum_{n=1}^{M}\frac{{a_{\left(u\right)}^{v}}^{2}\phi_{\left(t\right)}^{v\to h}\phi^{h\to v}\gamma_{\left(t\right)}^{v}\gamma^{h}}{\mu_{\left(u,t\right)}^{v}\left(\sigma^{h}+\mu^{h}\right)\left(\gamma^{h}+\mu^{h}\right)\left(\gamma_{\left(t\right)}^{v}+\mu_{\left(u,t\right)}^{v}\right)}=MP_{\left(u,t\right)}$$

(2) $${R_{e}}_{\left(u,t\right)}={R_{0}}_{\left(u,t\right)}S_{h}S_{v}$$

(3) $$P_{\left(u,t\right)}=\frac{\;{a_{\left(u\right)}^{v}}^{2}\;\phi_{\left(t\right)}^{v\to h}\;\phi^{h\to v}\;\gamma_{\left(t\right)}^{v}\;\gamma^{h}}{\mu_{\left(u,t\right)}^{v}\;\left(\sigma^{h}+\mu^{h}\right)\;\left(\gamma^{h}+\mu^{h}\right)\;\left(\gamma_{\left(t\right)}^{v}+\mu_{\left(u,t\right)}^{v}\right)}$$

There are a total of eight parameters in the expression of R0, four of which are climate independent (the human life span 1/*μ* ^*h*^, the transmission probability from infected human to mosquito per bite $\phi^{h\to v}$, the human infectious period 1/*σ* ^*h*^, and human incubation period 1/*γ* ^*h*^) and four are climate dependent (the life span of adult mosquitoes $1/\mu_{\left(u,t\right)}^{v}$, the extrinsic incubation period $1/\gamma_{\left(t\right)}^{v}$, the daily biting rate $a_{\left(u\right)}^{v}$ and the probability of transmission from infected mosquito to human per bite $\phi_{\left(t\right)}^{v\to h}$). Climate‐dependent parameters are defined as functions dependent on humidity (*u*) and temperature (*t*), which have previously been determined in experimental studies through laboratory estimates of entomological data under various climate conditions. A list of these functions, sources and details can be found in the Supporting Information S2 Text.

Quantification of R0 (and Re) requires an estimation of the number of adult female mosquitoes per human (M). It is rarely the case that adequate estimations of M exist for regions or mosquito species of interest. Given that the mosquito population size fluctuates in and out of season, M is expected to present oscillatory behaviour. Thus, the R0 (and Re) of a mosquito‐borne virus, dependent on M, also presents seasonal oscillations. However, such oscillations are only partially driven by M, and are also determined by $P_{\left(u,t\right)}$ (equation 1). In our mathematical framework, $P_{\left(u,t\right)}$'s contribution to this oscillatory behaviour stems from the four climate‐driven parameters: the life span of adult mosquitoes, the extrinsic incubation period, the daily biting rate and the probability of transmission from infected mosquito to human per bite.

Theoretically, the potential for outbreaks is determined by the epidemic thresholds of R0 >1 or Re >1. $P_{\left(u,t\right)}$ is a positive number that can be interpreted as the absolute potential of an adult female mosquito. Thus, if at least one female mosquito exists per human (M>=1) and $P_{\left(u,t\right)}>1$, then $R0=MP_{\left(u,t\right)}>1$ and epidemic growth is possible. However, interpreting $P_{\left(u,t\right)}$ on its own means that no direct assessment on the classic epidemic thresholds can be made. Similarly, a large number of mosquitoes per human (M>>1) can in theory compensate for a very small single‐mosquito transmission potential ($P_{\left(u,t\right)}<<1$) and no direct interpretations of very small P equating to no transmission potential should be made. Hence, in this manuscript, we do not make particular interpretations of $P_{\left(u,t\right)}>1$ or $P_{\left(u,t\right)}\approx 0$, but argue and demonstrate instead that the index P's absolute value is informative for the timing and amplitude of transmission when assessed locally in time or between regions.

$P_{\left(u,t\right)}$ can be estimated for any region for which humidity and temperature are available, and can be parameterized for any species of virus, host or vector. We thus define $P_{\left(u,t\right)}$ as the *mosquito‐borne viral suitability index P*. A key difference from other suitability indices not simply based on VC (e.g. Kraemer et al., 2015b; Messina et al., 2016), is that the numerical scale of the index P has direct biological interpretation: P is the reproductive (transmission) potential of an adult female mosquito. We note that since P takes into consideration factors that are not solely of entomological nature (e.g. transmission probability from infected human to mosquito $\phi^{h\to v}$, human mortality 1/*μ* ^*h*^ and human infectious period 1/*σ* ^*h*^), it is not entirely equivalent to reducing R0 to vectorial capacity (or the daily reproduction rate) (Brady et al., 2014; Gething et al., 2011; Hamlet et al., 2018a; Mordecai et al., 2017).

#### 2.1.2 Index estimation

In previous studies on ZIKV and DENV, we estimated R0 and Re by fitting a dynamic transmission model to notified epidemic curves within a Bayesian Markov chain Monte Carlo (bMCMC) framework, whereby unobserved parameters were estimated by their resulting posterior distributions (Faria et al., 2017a; Lourenço et al., 2017, 2014). Here, however, our goal is to estimate suitability for transmission, through $P_{\left(u,t\right)}$, independently of the availability of epidemiological data.

The Bayesian strategy implemented in MVSE focuses on defining priors for all the parameters in the expression of $P_{\left(u,t\right)}$ for which adequate support exists in the scientific literature. For the climate‐driven parameters ($\mu_{\left(u,t\right)}^{v}$, $\gamma_{\left(t\right)}^{v}$, $a_{\left(u\right)}^{v}$ and $\phi_{\left(t\right)}^{v\to h}$), which affect the amplitude and timing of transmission, such informed priors bound the possible solutions of $P_{\left(u,t\right)}$ for the virus, mosquito and region of interest. Code examples, methodological details and a step‐by‐step description on how the $P_{\left(u,t\right)}$ is estimated by MVSE can be found in S2 Text.

### 2.2 MVSE implementation, availability and requirements

**Figure 1:**
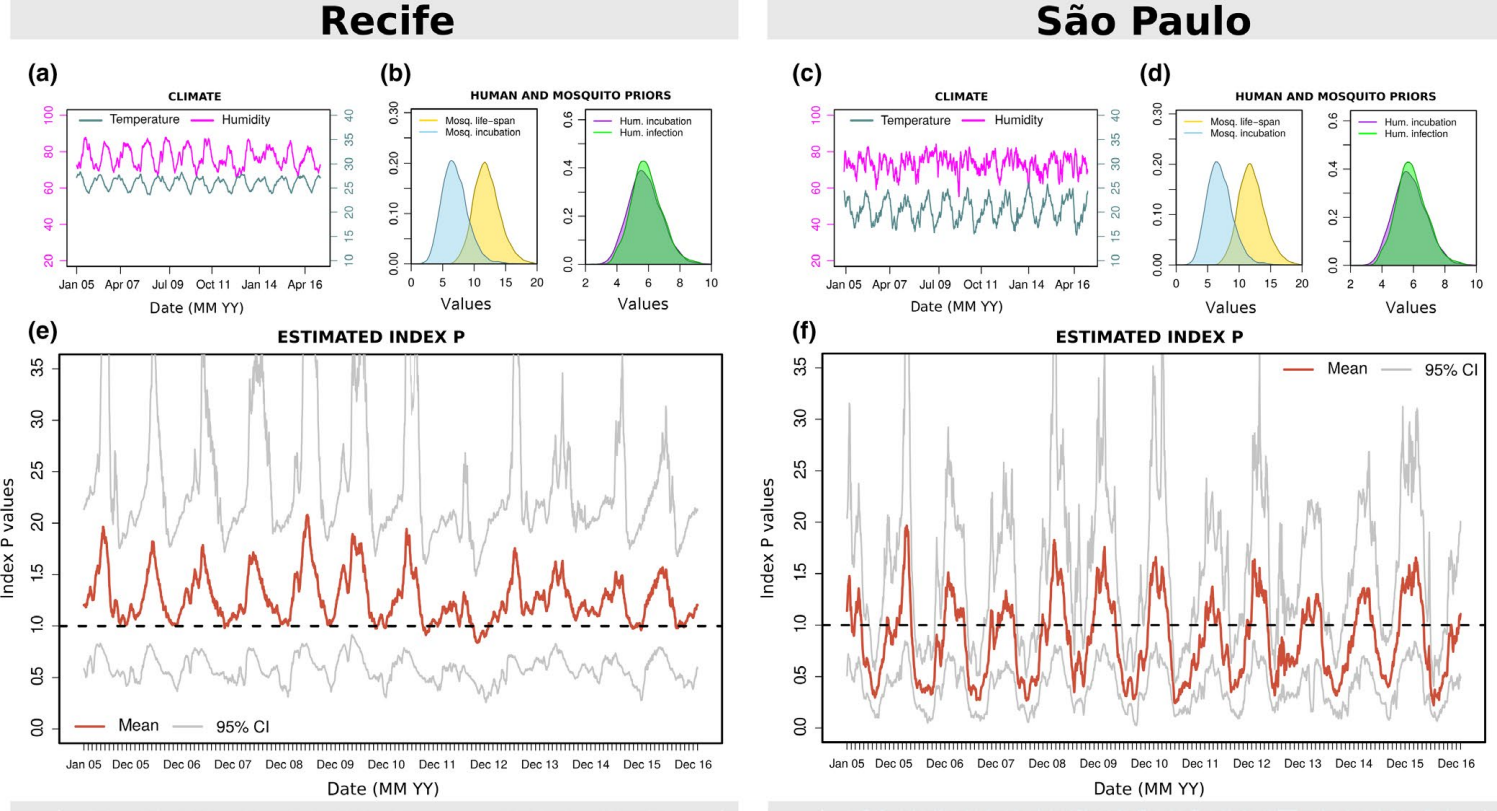
Climate, priors and estimated index P for Recife and São Paulo. (a, c) Local humidity (magenta) and temperature (turquoise) time series per day. (b, d) Examples of MVSE informed priors: mosquito life span (blue) and incubation period (yellow); human incubation (purple) and infectious (green) periods. (e, f) Estimated index P per day with mean (red) and 95% confidence interval (grey). Priors were assumed to be the same for Recife and São Paulo. See Supplementary Information S2 Text for prior distributions

**Figure 2:**
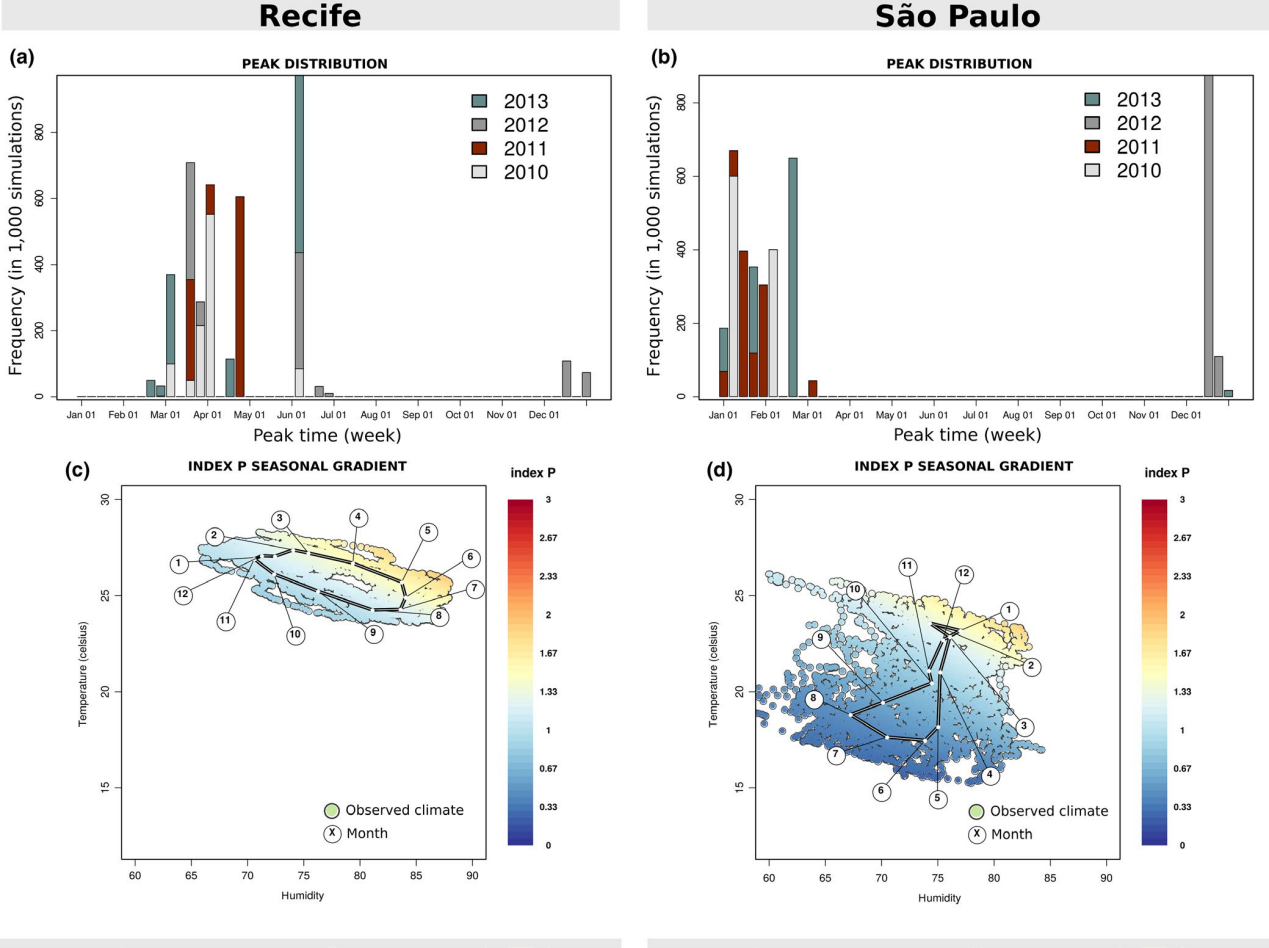
Seasonality of index P for Recife and São Paulo. (a, b) For each year coloured differently, the week with highest suitability is identified across all estimated index P solutions of that year (solutions’ mean and 95% CI in Figure 1). The frequency of each peak week shown across the 1000 simulations. (c,d) Two‐dimensional sensitivity of mean index P per humidity (x) and temperature (y) observed time point (day). Each point is a combination of observed climate variables, coloured according to the mean index P estimated (colour scale on the right). The white dots (over the black link) mark the mean humidity and temperature of each month over the period of the data (2005–2016); the floating circles with numbers identify each month's number

The R‐package MVSE is available under a GNU GPL 3.0 license at a SourceForge repository (sourceforge.net/projects/mvse/), where both a source‐based package file and PDF reference manual can be found. MVSE is platform‐independent, requiring R (>=3.4) and R‐packages *pbapply* (for user‐console feedback), *scales* (for plotting), *genlasso* (for time‐series smoothing). The version of the package used in this manuscript is v0.3 (cryptonym: spectral). Newer versions and related materials will be deposited in this repository and we refer the reader to it for further information and future changes. Detailed technical features of the package can be found in S2 Text, including details on the climate‐driven functions for ento‐epidemiological parameters, and priors used in the main text. MVSE code examples for the estimation steps of the index P, including a complete example that reproduces Figure 1, 2 and Figures S1–S4 (S1 Text) can also be found in S2 Text.

## 3 SELECTION OF RESULTS

### 3.1 Index P dynamics in Recife and São Paulo

We first present a comparison of the estimated index P per day in two major urban centres—Recife and São Paulo (Brazil)—for the time period between 2005 and 2016. Recife is located in the state of Pernambuco in the north‐east of Brazil with a tropical savanna climate, while São Paulo is located in the south with a humid subtropical climate (Köppen climate classification (Peel, Finlayson, & McMahon, 2007). The two cities are known to have contrasting epidemic and endemic histories for mosquito‐borne viruses. For instance, Recife has experienced a large ZIKV epidemic and is known to be endemic to DENV and CHIKV (de Araújo et al., 2016; Magalhaes et al., 2017; Sabino et al., 2016). In contrast, São Paulo is yet to experience a sustained ZIKV epidemic, DENV incidence is known to be low for the national average (Teixeira, Siqueira, Ferreira, Bricks, & Joint, 2013) and transmission of mosquito‐borne viruses is unstable, with phylogenetic studies showing that epidemics are seeded by new lineages almost every year (Faria et al., 2017a,2017b). In the notified dengue data that we obtained for 2007–2012 (See Data accessibility3), the total incidence per 100k individuals in Recife was approximately 11‐fold higher than it was in São Paulo (2184.3 notifications vs. 195.2).

The input (climate time series, user‐defined priors) and index P output for the two cities is summarized in Figure 1 (for a more complete set of possible outputs see S1 Text). Here, for simplicity, and for a lack of evidence of significant differences between the two cities, we assume all informed priors to be the same (Figures 1b,d). As expected, differences can be seen in the local climatic trends, with São Paulo showing, for instance, lower temperatures compared to Recife (Figures 1a,c). Figures 1e,f show the estimated index P for the two cities, which present significantly different dynamics. For instance, the mean index P in Recife is estimated to be 1.5‐fold higher than in São Paulo (1.26 vs. 0.84). Seasonality is also different, with Recife showing less pronounced oscillations and with mean P generally maintained above 1 (i.e. one female mosquito per human would necessarily be enough for transmission, equation 1). In contrast, São Paulo shows pronounced oscillations, with P presenting substantial troughs.

These key differences in index P are in accordance to what is known about the transmission potential of mosquito‐borne viruses in the two cities. For instance, the stabler seasonality patterns and higher index P in Recife suggests year‐round potential for single adult mosquitoes to contribute to epidemic expansion, and can thus help explain why dengue incidence in Recife can be one order of magnitude higher than in São Paulo. Concurrently, the substantial troughs of index P estimated in São Paulo suggest very low transmission potential off‐season, which may partially explain why persistence of mosquito‐borne viral lineages between seasons is rarely observed in the city.

### 3.2 Index P seasonality in Recife and São Paulo

We next present two visual outputs from MVSE, comparing some of the seasonal differences in the estimated index P between Recife and São Paulo (for a more complete set of possible outputs see S1_Text). Figure 2 shows the timing of highest index P and sensitivity of P to each of the climatic variables. For visual convenience, we restrict MVSE's output to the period of 4 years, between 2010 and 2013.

For each year and city, the week with highest suitability is identified across all estimated index P solutions (*simulations*, of which the mean and 95% CI is in Figures 1e,f). As seen in Figures 2a,b, the two cities vary in their detected peak weeks. In Recife, suitability tends to peak between March and May (regional autumn), with the occasional occurrence of higher suitability in June (in particular in the years 2012 and 2013). In contrast, suitability peaks earlier in São Paulo, between late December and early February (regional summer).

MVSE also offers visual output which highlights the regional sensitivity of the index P to climatic changes in time (Figures 2c,d). Once again, the differences between the cities are clear. Humidity and temperature values are less variable in Recife than in São Paulo, and Recife has both higher humidity and temperature. This visualization also reveals a clear gradual trend in suitability across the months in Recife. In contrast, this gradient is only clear for São Paulo in the winter months (May–September).

### 3.3 Index P spatiotemporal characterization across Brazil

**Figure 3:**
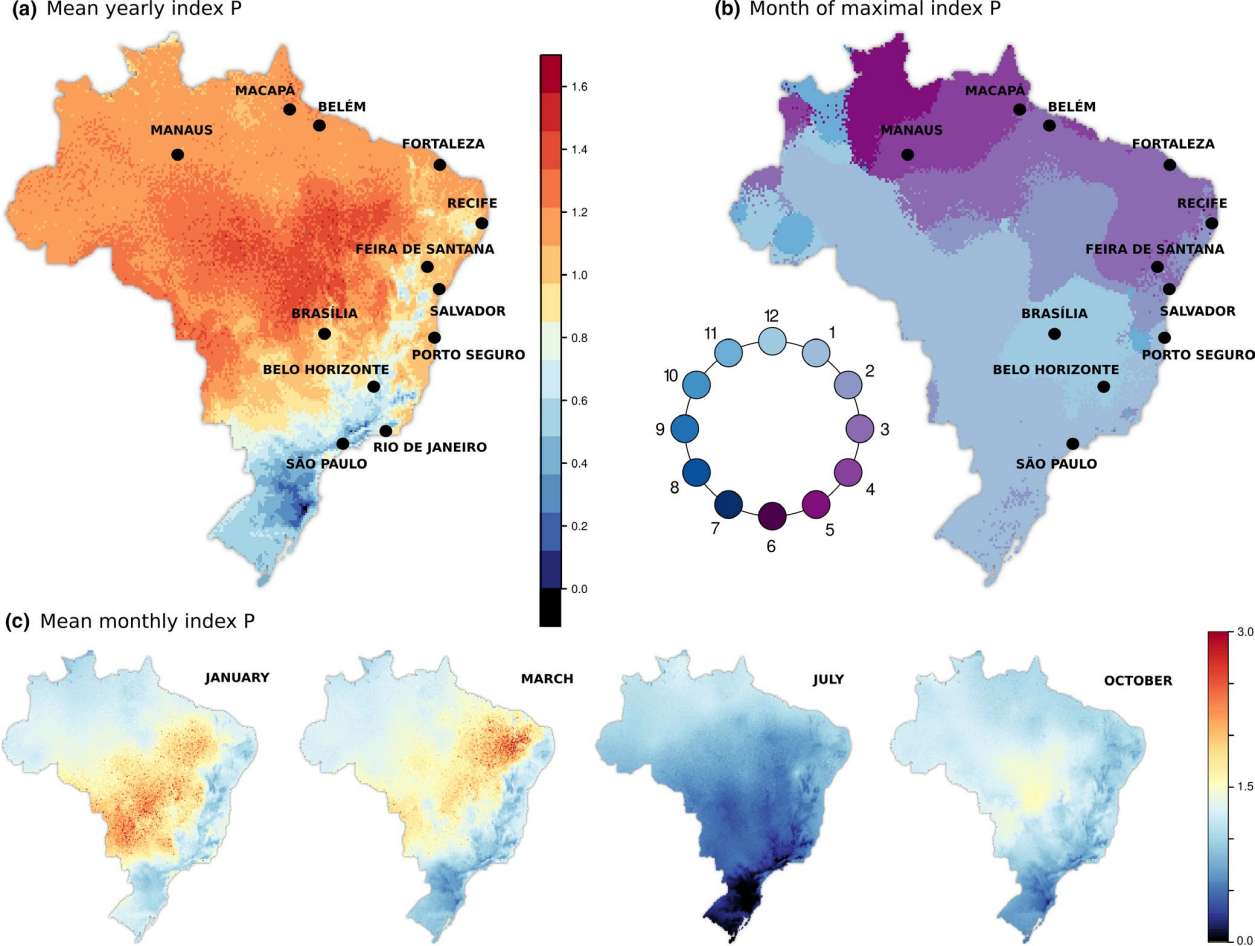
Spatiotemporal characterization of index P across Brazil. (**a**) Map presents the mean index P per pixel ($\approx 340{\text{Km}}^{2}$). Values coloured according to scale on the right. (b) Using the estimated index P of each pixel, with 12 points representing months, the month with highest index P is identified. Each pixel is coloured according to that month, with the colour scale represented in a circle. (c) Same as (a), but presenting the mean for selected months. In all maps, priors were assumed to be the same per pixel, as used for Recife and São Paulo. See Supplementary Information S2 Text for prior distributions. These solutions are made available as Supplementary Files

In the previous figures, we demonstrated how the index P can be used to compare transmission potential and timing between two Brazilian cities. In this section, we demonstrate the usefulness of index P to characterize the transmission potential across vast geographical ranges, using Brazil as a case study. We use the WorldClim V2 dataset, which holds mean world‐wide climatic data per month, during 1970–2000 (see Data accessibility3 for details). Our analysis is performed over a regular grid of Brazil, in which each pixel represents an area of $\approx 340{\text{km}}^{2}$. For each pixel, we use 12 time points (months) of humidity and temperature to run MVSE and estimate the dynamics of index P over 1 year. Although computationally intensive, due to the size of the grid, this exercise only employs functions available in MVSE. For further details on the data, and outputs of a similar exercise over the entire South American continent, see S2 Text. Figure 3 presents a series of maps derived from MVSE's output.

The estimated mean suitability presents high variation across Brazil (Figure 3 a). As expected, the southern regions present the lowest transmission potential (colder colours, *p *< 0.6), while the highest potential is estimated in the centre and along the northern coast (warmer colours, *p* > 1.0). The elevated regions of the country, running in parallel to the eastern coast, present a pattern of intermediate suitability (P≈0.8) close to half the maximum potential presented elsewhere (P≈1.7).

We further identify the month of peak suitability for each region (Figure 3 b). This discretization highlights a seasonal gradient in the month of maximal suitability across Brazil. In the southern regions, peak suitability is during the early summer months, and moving north along the eastern and then northern coasts shifts peak suitability further into regional autumn. Notably, no region of Brazil is seen to experience peak suitability during the core winter months (July–September, darker blue).

We also map the mean monthly index P for a subset of months (Figure 3c). In these maps, the generally lower suitability in the south is clearly highlighted, as is the fact that patches of higher suitability occur at different months in different regions.

### 3.4 Index P and notified cases in Brazilian cities

In Figures 1 and 2, we characterized the index P for the cities of Recife and São Paulo. We use climate data from 2007 to 2012 for these and 13 other cities in Brazil, and present the correlation between the estimated suitability and notified dengue cases. Access to adequate climatic data restricted the number of cities we could explore, but with this small set of cities we aimed at presenting examples along the coastal line of Brazil, which present interesting geographical and temporal variation in suitability (shown in Figure 3).

Notifications were originally per month, for every year over the period 2007–2012 (see Data accessibility3 for details). We estimated index P per day in the period 2007–2012, and then calculated mean P for each of the 12 months across those years. We also calculated the mean of dengue notified cases for each of the 12 months across those years. By averaging across 5 years, we aimed at quantifying suitability into what can be termed a *typical year* that is the expected average monthly suitability of each city in any year.

**Figure 4:**
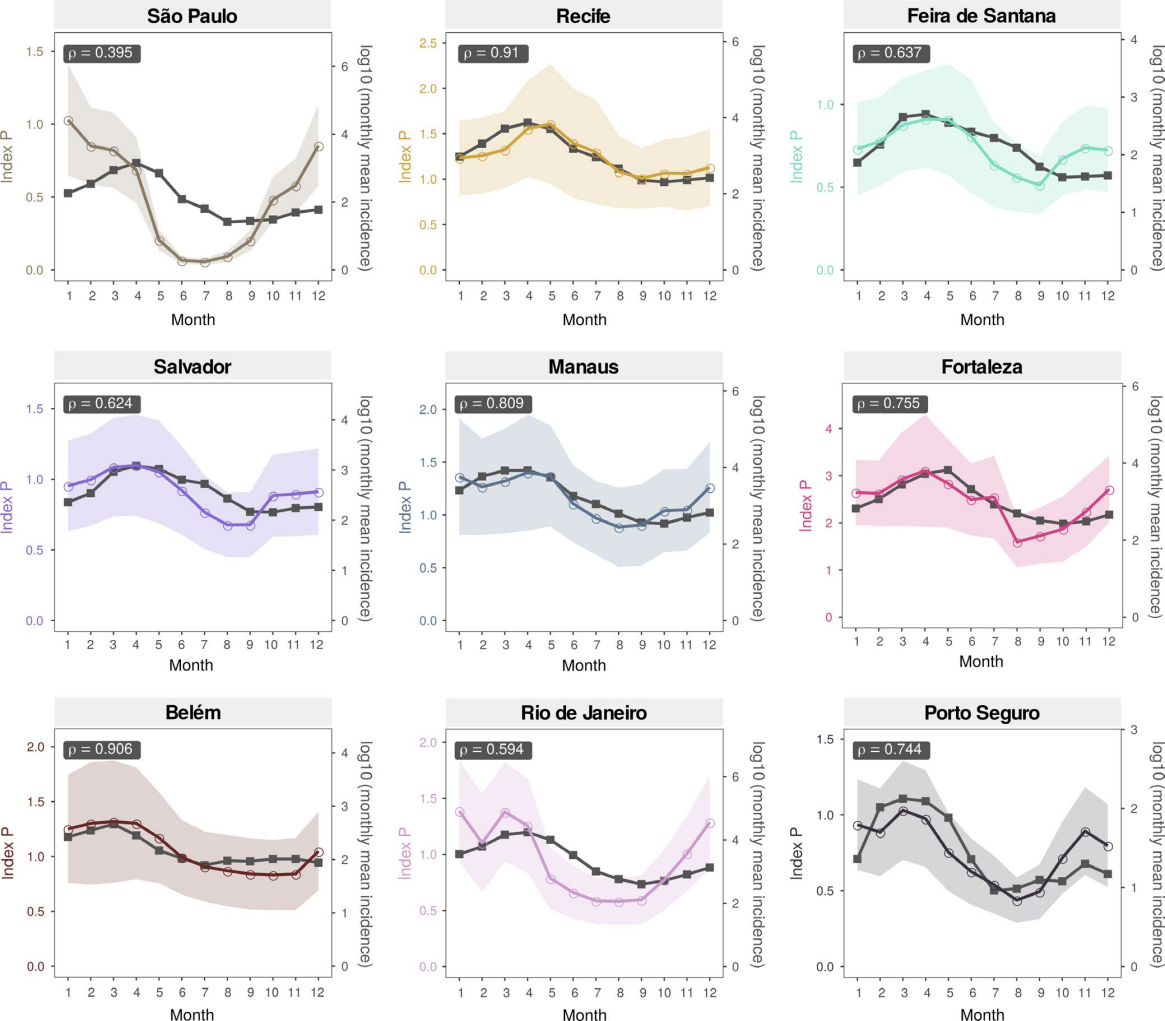
Correlation of index P and notified cases in nine Brazilian cities. Monthly mean time series of index P and dengue notified case data (period 2007–2012) are shown for nine cities (in order, per row, left to right): São Paulo, Recife, Feira de Santana, Salvador, Manaus, Fortaleza, Belém, Rio de Janeiro and Porto Seguro. The geographical location of these cities is shown in the maps of Figure 3. Pearson's correlation coefficient (ρ) is shown within each subplot. The coloured shaded areas standard deviation of the index P estimates. Six other cities that widen the geographical range of the examples are presented in Figure S5 in S1 Text. Sensitivity of ρ (Pearson's) to two priors (biting rate and mosquito life span) are presented in Figures S6–S7 in S1 Text

Figure 4 presents the typical year suitability against the case notifications, per city. Pearson's correlation (ρ) shows that the index P is highly and positively correlated with dengue notifications across several Brazilian cities (for more cities see Figure S5 in S1 Text). Interestingly, the correlations of the index with data are lower for São Paulo and Rio de Janeiro–the two most southern cities and the ones within areas estimated to have lower yearly index P (blue colours, Figure 3a). It is possible that in such regions, other demographic and/or entomological factors not considered in the estimation of the index P may become more relevant, or that stochastic effects are stronger in effective transmission. In general, these results are similar to results we have previously obtained for Myanmar at the country and city levels (Perez‐Guzman et al., 2018).

### 3.5 Index P and the *Aedes aegypti* suitability score

Finally, we set out to demonstrate that the simple approach underlying the index P can be as informative as another widely used suitability measure. For a robust comparison, we looked for a case study for which we could find (a) estimations of an alternative suitability measure, (b) incidence for three mosquito‐borne viruses (CHIKV, DENV and ZIKV) at two spatial scales (city, country) and (c) temperature and humidity series from local weather stations. The selected case study was of Honduras and its capital city Tegucigalpa (capital city), for which we had those data after having recently explored it in the context of the emergence of ZIKV in the Americas (Thézé et al., 2018)

The suitability measure commonly known as the *Aedes aegypti* suitability score (AaS) was considered for comparison (Brady et al., 2014; Kraemer et al., 2015b; Messina et al., 2016). AaS includes a myriad of variables that are known to affect entomological and viral factors but which are not included in the formulation of the index P—for instance, precipitation, vegetation levels, urbanization levels and geo‐occurrence (reports of) adult or aquatic forms of *Ae. aegypti* and *Aedes albopictus* (Kraemer et al., 2015b). The implementation of AaS is explicitly spatial, with temporal and spatial resolutions restricted by the underlying WorldClim dataset used (maximum resolution of 12 months and spatial resolution from 1 to 340 ${\text{km}}^{2}$ per geopixel). To follow the 12 month time‐scale of AaS, we transformed the three weekly incidence time series into months by adding up each month's weeks.

**Figure 5:**
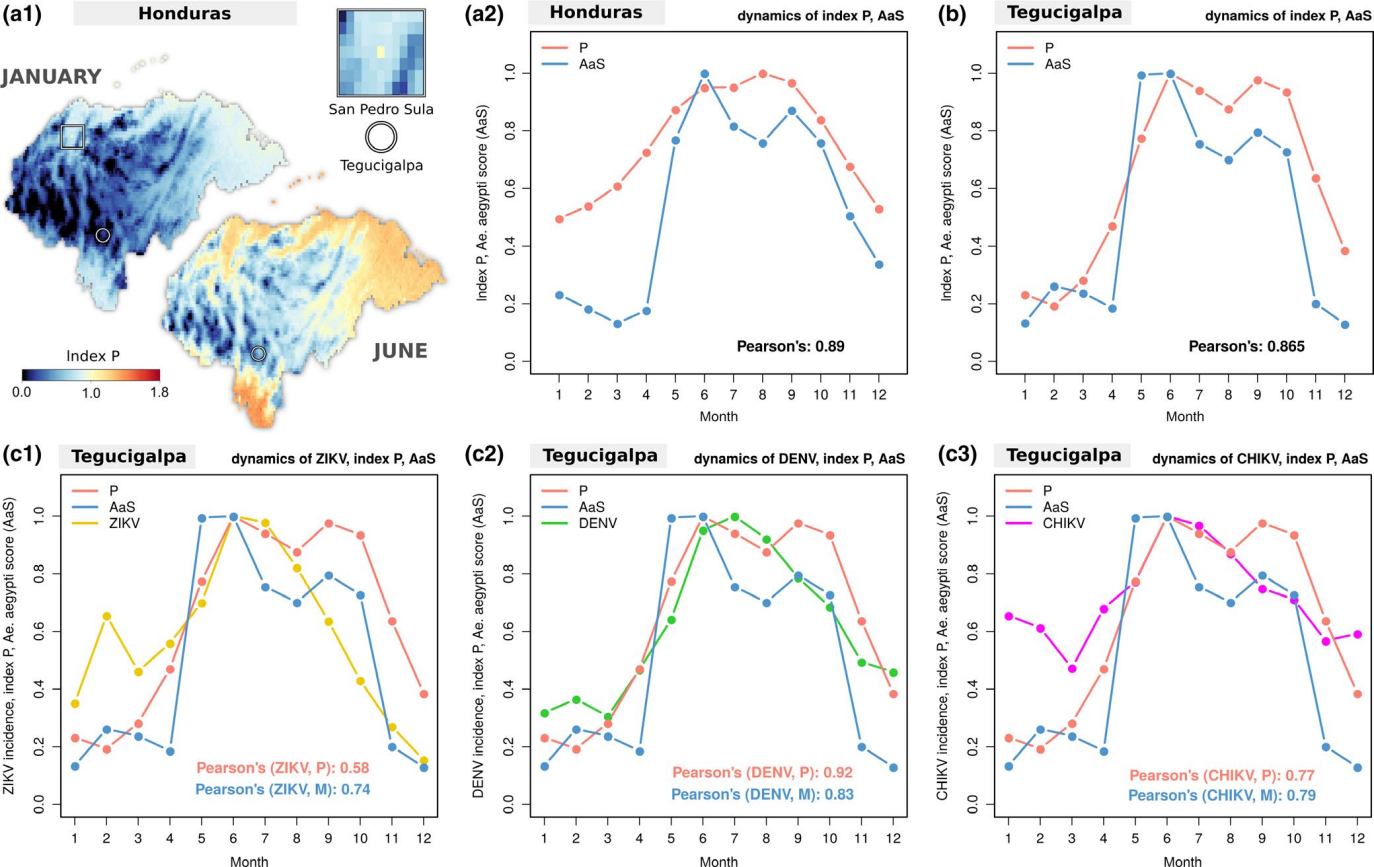
Index P across Honduras and its relationship with mosquito‐borne viruses and Aedes aegypti suitability score in the capital city. (a1) Maps of Honduras showing estimated index P at 25 ${\text{km}}^{2}$ (selected months). Tegucigalpa (the capital city) and San Pedro Sula (city) are highlighted on the maps (circles and square respectively). The inset square presents a close‐up of the region of San Pedro Sula, in which the centre geopixel is the city exhibiting high index P values relative to its surroundings. (a2) Typical year index P (red) and *Aedes aegypti* suitability score (AaS, blue) per month at the country level with Pearson's correlation of 0.89. Index P at each month is the mean across all geopixels (e.g. maps in (a1)). AaS is the score of Figure 3 in Thézé et al. (2018). (b) Typical year index P (red) AaS (blue) per month at the city level with Pearson's correlation of 0.865. Index P at each month is the mean of P at each month between 2005 and 2013 when using temperature and humidity per day from a local weather station (shown in Figure S8 in S1 Text). AaS is the score of Figure 3 in Thézé et al. (2018). (c1) Same P and AaS as in (b), and Zika virus (ZIKV) incidence per month (yellow, for 2016). (c2) Same P and AaS as in (b), and dengue virus (DENV) incidence per month (green, for 2015). (c3) Same P and AaS as in (b), and chikungunya virus (CHIKV) incidence per month (magenta, for 2015). (c1–c3) Pearson's correlation for each pair of suitability measure vs incidence is presented within each subplot. In the legend of Pearson's correlation, M refers to AaS. (b‐c3)All variables are normalized to 0–1 by their maximum value for visualization purposes. Incidence variables are log10 before being normalized

At the country level, we estimated index P in time and space using the same approach as we used for Brazil (Figure 3) and South America (S2 Text). The spatial dimension (Figure 5 a1) revealed a year‐round low index P in regions with high elevation, known to present the lowest DENV and CHIKV incidence in the country (Zambrano et al., 2017). We found numerous similarities to previously published AaS maps of Honduras (Thézé et al., 2018). For instance, the index P presented widespread low suitability in January and high suitability in June, while northern and eastern regions had year‐round higher P compared to the rest of the country in the same months. We also found the geopixel representative of the location of San Pedro Sula to present an unusually high index P in January compared to nearby locations (seen in the inset square of Figure 5 a1, with P≈1.15 for the city, vs. P≈0.70 elsewhere). Critically, this was also observed for the same geopixel when estimating AaS, which has helped Thézé and colleagues explain why the first ZIKV epidemic in Honduras peaked in January in San Pedro Sula, and in June in Tegucigalpa (Thézé et al., 2018). To compare the index P directly to the monthly AaS estimation at the country level, we averaged the index P in space per month (e.g. for January, P was the mean of the values presented in the map of Figure 5a1). The resulting Honduras index P was highly correlated with the AaS estimated in a similar manner (Figure 5 a2).

At the city level, we estimated index P in time using daily temperature and humidity data during the period 2005–2013 (from a weather station at the centre of Tegucigalpa, see Data accessibility3). Similarly to what was done for the Brazilian cities (Figure 4), we averaged P over each month across the years. The AaS estimation was performed as in Thézé et al., based on a geopixel located within the geographical boundaries of Tegucigalpa (Thézé et al., 2018). The resulting Tegucigalpa index P was highly correlated with the AaS estimates (Figure 5b).

We next compared the correlations of both the index P and AaS with incidence patterns for ZIKV, DENV and CHIKV at the city level (Figures 5c1‐c3). The two suitability measures performed equally well for CHIKV, whereas AaS performed better for ZIKV, and index P better for DENV.

Overall, both at the country and city levels, the two suitability measures were highly correlated, and performed similarly in regards to the temporal patterns of three vector‐borne viruses. Given that the index P ignores precipitation, vegetation levels, urbanization levels and mosquito geo‐occurrence, it performed remarkably well. Furthermore, the index P can be estimated at a higher temporal resolution, allowing it to be analysed at the original weekly scale of the incidence time series (presented in Figure S8 in S1 Text with a Pearson's correlation of 0.9).

## 4 POSSIBLE LIMITATIONS AND FUTURE DIRECTIONS

The index P uses a simple approach to achieve a complex goal, taking into consideration only two variables (temperature and humidity) as well as a few priors on mosquito, human and virus factors. Our approach does not consider a wide variety of explanatory variables shown or suggested to affect mosquito dynamics. Namely, we do not consider altitude (Watts et al., 2017), number of female mosquitoes per human (Brady et al., 2014; Gething et al., 2011), vegetation (Kraemer et al., 2015a,2015b; Messina et al., 2016; Yanoviak, Paredes, Lounibos, & Weaver, 2006), urbanization (Kraemer et al., 2015a,2015b; Messina et al., 2016), climate and adult flight performance (Rowley & Graham, 1968), precipitation (Gardner, Boťa, Gangavarapu, Kraemer, & Grubaugh, 2018; Kraemer et al., 2015a,2015b; Messina et al., 2016), temperature and the probability of infection from humans to vector (Liu‐Helmersson et al., 2014), socio‐economic factors (Gardner et al., 2018; Mordecai et al., 2017), human mobility (Gardner et al., 2018; Kraemer et al., 2015a), or deforestation and land use (Gardner et al., 2018; Norris, 2004; Yanoviak et al., 2006).

In the current version of MVSE, we show that the index P only has low explanatory power (Pearson's ρ<0.5 with long‐term DENV incidence) for 2 out of 15 Brazilian cities (Figures 4 in main text and S5 in S1 Text). In Figure 5 (main text) and Figure S8 (S1 Text), we also demonstrate that P can have similar performance to the widely used *Ae. aegypti* suitability score in a case study including two spatial (city, country) and temporal (weeks, months) resolutions. We plan to update the theoretical framework that, in the future, will allow to consider the contribution of variables beyond temperature and humidity. The educational and research benefits of their inclusion will be explored in future publications.

In the main results, we have focused on mosquito‐borne viruses which are typically maintained in a human–mosquito transmission cycle. This is because the expressions of R0 and index P (equations 1 and 3) are derived from a dynamic model which considers only one main host (here parameterized as a human host). This single‐host formulation has implications when applying the index P to other viruses whose epidemiology critically depends on multiple hosts—for example, WNV and YFV for which birds and primates (respectively) are necessary hosts. For these particular examples, human infections are mainly spill over events from the zoonotic cycle (e.g. for YFV see [Faria et al., 2018]). Thus, while the single‐host formulation should be minded, a potential use of the index P is to parameterize suitability to the reservoir–host and reservoir–mosquito species, and interpret P as a proxy for risk of spill over to human populations.

Finally, we note that contrary to other suitability measures such as AaS (Figure 5), the index P cannot assess the likelihood of the presence or absence of a mosquito species. This is because P ignores the number of adult female mosquitoes per human (M). In fact, the index P can have a non‐zero value for a region in which a mosquito of interest is not present. In this case, the index P can be interpreted as the natural potential of a single female mosquito to transmit a particular virus once introduced into the region. One potential line for future research is to integrate the index P with mosquito suitability measures such as AaS to assess the likelihood of both the presence of the mosquito and its capacity for transmission of a particular virus in the presence of a specific host.

## 5 CONCLUSIONS

In this manuscript, we have introduced in detail a mosquito‐borne viral suitability measure which we named index P. We apply and exemplify the capacities of the index P to estimate transmission potential and characterize it in space and time using real world examples. We also introduce and provide MVSE, an R‐package that implements various functionalities allowing to estimate and analyse the index P in regions for which humidity and temperature time series are available.

We believe that both MVSE and the index P are unique in the current literature. Other suitability approaches exist and have been widely used, but are generally based on complex computations, dependent on many variables from various data sources (not necessarily available), with little direct biological interpretation on the measured scale, and critically, for which no freely available (estimation) software tools exist. In contrast, index P is simply based on the R0 expression of a mosquito‐borne dynamic model and two climate variables, has a direct biological interpretation, and is easily calculated and explored through MVSE, a complete and freely available software tool.

With its aforementioned mathematical simplicity, dependency on only two climatic variables at any possible time‐scale (hours, days, weeks, months) and freely available R‐package MVSE, we foresee the potential of the index P not only for direct research purposes, but also for education (e.g. epidemiological courses on arboviruses) and field ento‐epidemiology (e.g. for entomologists and epidemiologists collecting real‐time data). New ideas and methodological updates will be implemented in future versions of MVSE as the user base will keep growing and our own research projects on mosquito‐borne viruses’ progress. Any future updates to the R‐package will be made available in the package's online repository (see Theory, Design and Implementation1).

## COMPETING INTERESTS

The authors declare that they have no competing interests.

## AUTHOR'S CONTRIBUTIONS

J.L. developed the index P theory. P.N.P. demonstrated the theory in the first case studies. J.L., C.J.V.A. and N.R.F. planned the development of the R‐package. J.L. and U.O. curated the data, coded the R‐package, performed the experiments and wrote the manuscript. J.T. supervised the Honduras case study and provided essential data sources. All authors tested the code, and revised both the examples and manuscript text.

## Data Availability

Climatic data (average relative humidity and minimum temperature) were collected from the Brazilian public repository ‘Meteorological database for education and research’ (banco de dados meteorológicos para ensino e pesquisa) available at the BDMEP website for several cities of Brazil (period 2005–2016). These data are free to access, use and archive (conditions at INMET website). The climatic data used are made available on Figshare https://doi.org/10.6084/m9.figshare.c.4485503 (Louren¸co, 2019). WorldClim V2 global data (Fick & Hijmans, 2017) were downloaded from a public repository online (https://www.worldclim.org/). Updated in July 2016, these data contain average monthly climatic measurements, representative of the period 1970–2000. The spatial resolution used for the maps of Brazil and South America was of 10 minutes ($\approx 340{\text{km}}^{2}$). The spatial resolution used for the maps of Honduras was 2.5 minutes ($\approx 25{\text{km}}^{2}$). These data are not made available in this manuscript due to license restrictions, but are publicly available at WorldClim webpage (https://www.worldclim.org/). See Supporting Information S2 Text for more details. Dengue notification data (by clinical evaluation, not molecularly confirmed) were obtained from the Brazilian ministry of health, available at the TABNET webportal. These notification data included the total number of dengue cases per month, per year, over the period 2007–2012 for the main municipality of each city. The data used are made available on Figshare https://doi.org/10.6084/m9.figshare.c.4485503 (Louren¸co, 2019). Dengue and chikungunya incidence from Tegucigalpa (city) for the year 2015 were taken from the study by Zambrano et al. (2017) with permission from Elsevier (license number 4576391190534). Counts were collected by the national surveillance system and refer to cases clinically and laboratory confirmed (by serology and RT‐PCR). Data series were extracted from Figure 5 using Engauge Digitizer Software V10.4 (https://doi.org/10.5281/zenodo.2585387). Zika incidence from Tegucigalpa (city) for the year 2016 is the same as in our previous study by Thézé et al. (2018). Counts refer to suspected, not confirmed cases. The data used are made available on Figshare https://doi.org/10.6084/m9.figshare.c.4485503 (Louren¸co, 2019). *Aedes aegypti* suitability score for Tegucigalpa (city) and Honduras (country level) is the same as in our previous study by Thézé et al. (2018). The data used are made available on Figshare https://doi.org/10.6084/m9.figshare.c.4485503 (Louren¸co, 2019). Climate data (minimum temperature and humidity 2005–2013) per day for Tegucigalpa (city) was obtained from the National Centers for Environmental Information of the National Oceanic Atmospheric Administration (NCDC NOAA) (free to be accessed at https://www.ncdc.noaa.gov). The weather station ID was 78720099999, located at the Toncontin International Airport. The data used are made available on Figshare https://doi.org/10.6084/m9.figshare.c.4485503 (Louren¸co, 2019).
