# Supplementary material for: MVSE: An R‐package that estimates a climate‐driven mosquito‐borne viral suitability index
Source: Methods Ecol Evol. 2019 Jun 19;10(8):1357–70. doi: 10.1111/2041-210X.13205 (PMC7202302; doi:10.1111/2041-210X.13205)
Supplement: Supplementary file 1 [file MEE3-10-1357-s001.zip › mee313205-sup-0003-TextS1.pdf]

Appendix: Complete visual output (supplementary figures).

MVSE: an R-package that estimates a climate-driven mosquito-borne viral suitability index.

December 27, 2018

Here we include and describe the complete set of visual output for the results presented in the main text. We expand on observations and methodological details in the figure legends. This supporting file complements the examples and results of the main text.

List of Figures

1 **Entomological parameters, generation time and vectorial capacity.** 2
2 **Index P trend filtering and threshold crossing timing.** 3
3 **Heatmaps for empirical and theoretical index P.** 4
4 **Heatmaps for generation time and vectorial capacity.** 5
5 **Pearson's correlation of index P and dengue notifications for 6 cities in Brazil.** 6
6 **Sensitivity of Pearson's correlation between index P and dengue notifications for Brasília, Rio de Janeiro, Boa Vista, Maceió, Fortaleza, Curitiba, São Luis, Porto Seguro.** 7
7 **Sensitivity of Pearson's correlation between index P and dengue notifications for Belo Horizonte, Recife, Salvador, Feira de Santana, São Paulo, Manaus, Porto Alegre, Belém.** 8
8 **Index P, DENV incidence and Aedes aegypti suitability score in Tegucigalpa and Honduras at different time scales.** 9

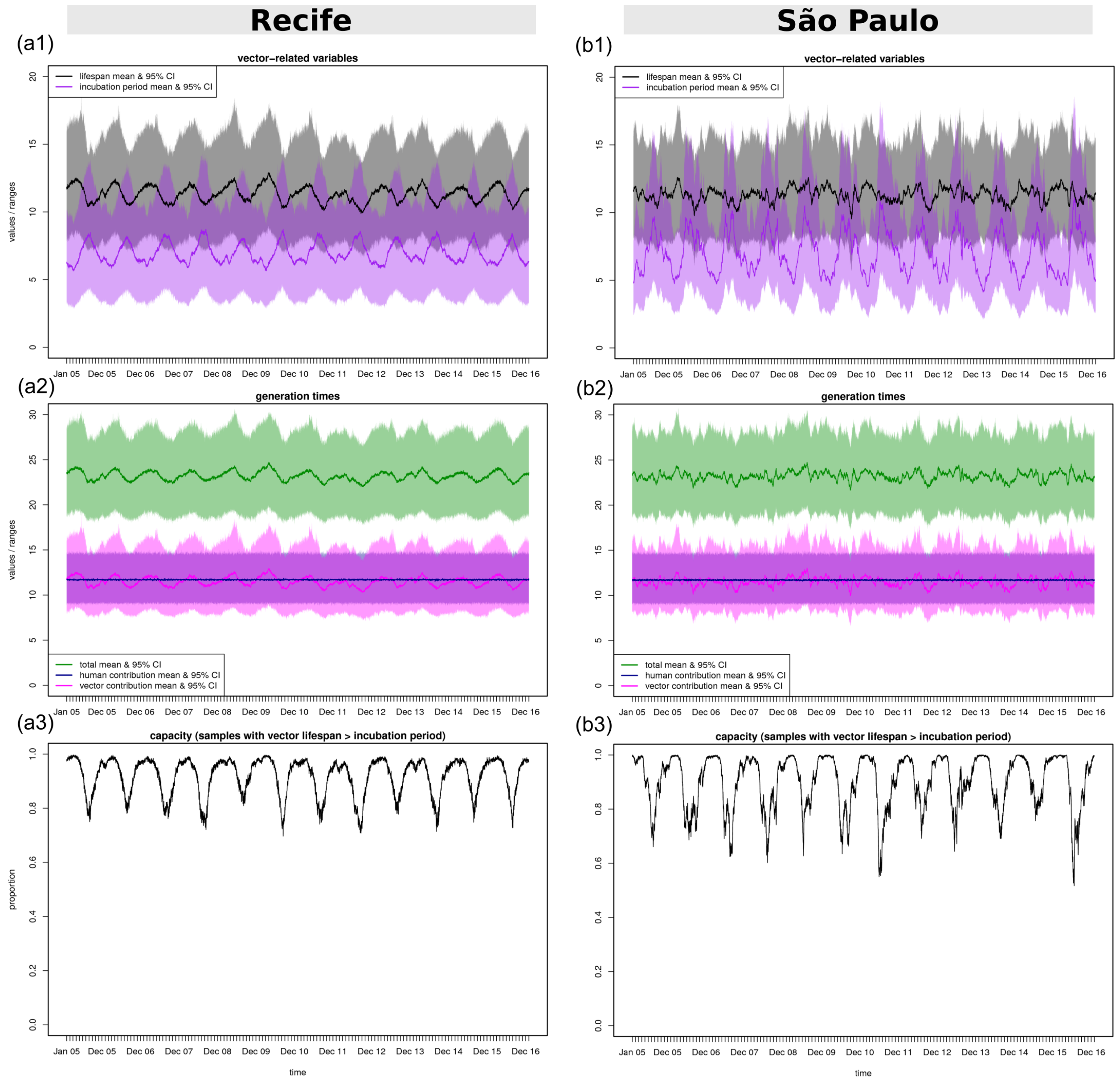

Figure 1: **Entomological parameters, generation time and vectorial capacity.** Recife (a1-3) and São Paulo (b1-3). **(a1,b1)** Adult mosquito lifespan (black) and mosquito incubation period (purple) in time. Full lines are the mean, shaded areas the 95% CI. ***Observations / interpretation:*** *Mosquito lifespan and incubation period oscillate in time due to natural climate fluctuations. The variables present a negative relationship, with periods of longer incubation synchronized with shorter lifespans. This phenomenon is known to be a key determinant of vectorial capacity. The noisy and less clear seasonal signal in lifespan for São Paulo is a result of almost no fluctuations in humidity.* **(a2,b2)** Generation time (GT, green) and the human (blue) and mosquito (magenta) independent contributions GT. Full lines are the mean, shaded areas the 95% CI. ***Observations / interpretation:*** *GT oscillates, as the mosquito contribution fluctuates due to the negative relationship between lifespan and incubation period. GT is an increasing function of lifespan, as longer lived mosquitoes imply a longer time range for infectious bites.* **(a3,b3)** Vectorial capacity (VC, black). ***Observations / interpretation:*** *VC dips when lifespan is shorter and incubation period is longer. In São Paulo, VC presents deeper troughs.* **MVSE details:** Visual output can be generated with the function *plotEntoParameters()*. Parameters used are the same as in the main text for both regions.

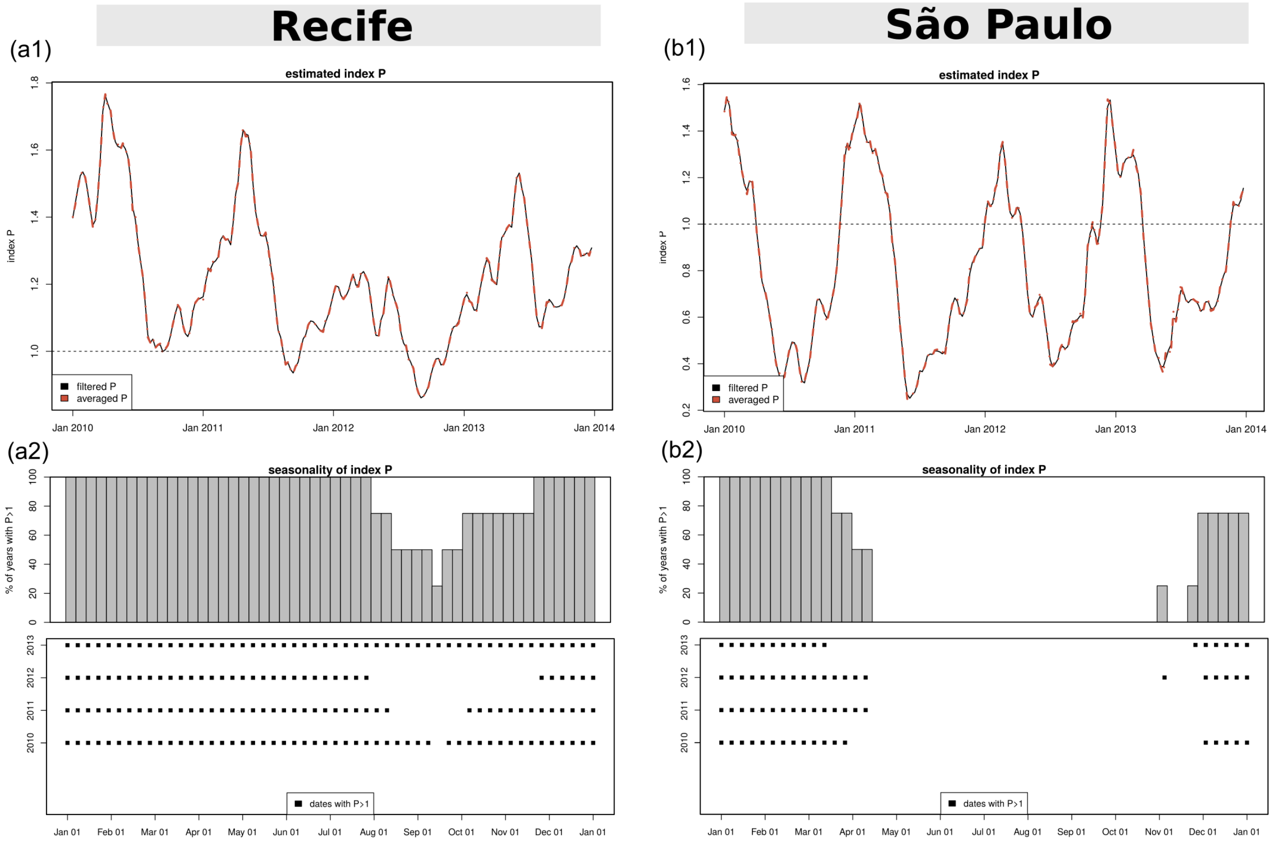

Figure 2: **Index P trend filtering and threshold crossing timing.** Recife (a1-2) and São Paulo (b1-2). **(a1,b1)** Trend filtering (black) of mean index P (dashed red). ***Observations / interpretation:*** *The filtered index P follows the mean index P closely.* **(a2,b2)** Identification of weeks for which index P is above 1 (black squares for yes) per year (bottom subpanel). Percent of years for which the index P was above 1 (grey bars, top subpanel). ***Observations / interpretation:*** *Recife's index P values remain mostly above 1 throughout the observed years, except for the period between August and November. For São Paulo, the index P spends most time under 1, with a clear signal for P>1 between December and late March.* Visual output can be generated by the *suitableSeason()* function. **MVSE details:** Parameters used are the same as in the main text for both regions. Years chosen for analysis: 2010-2013.

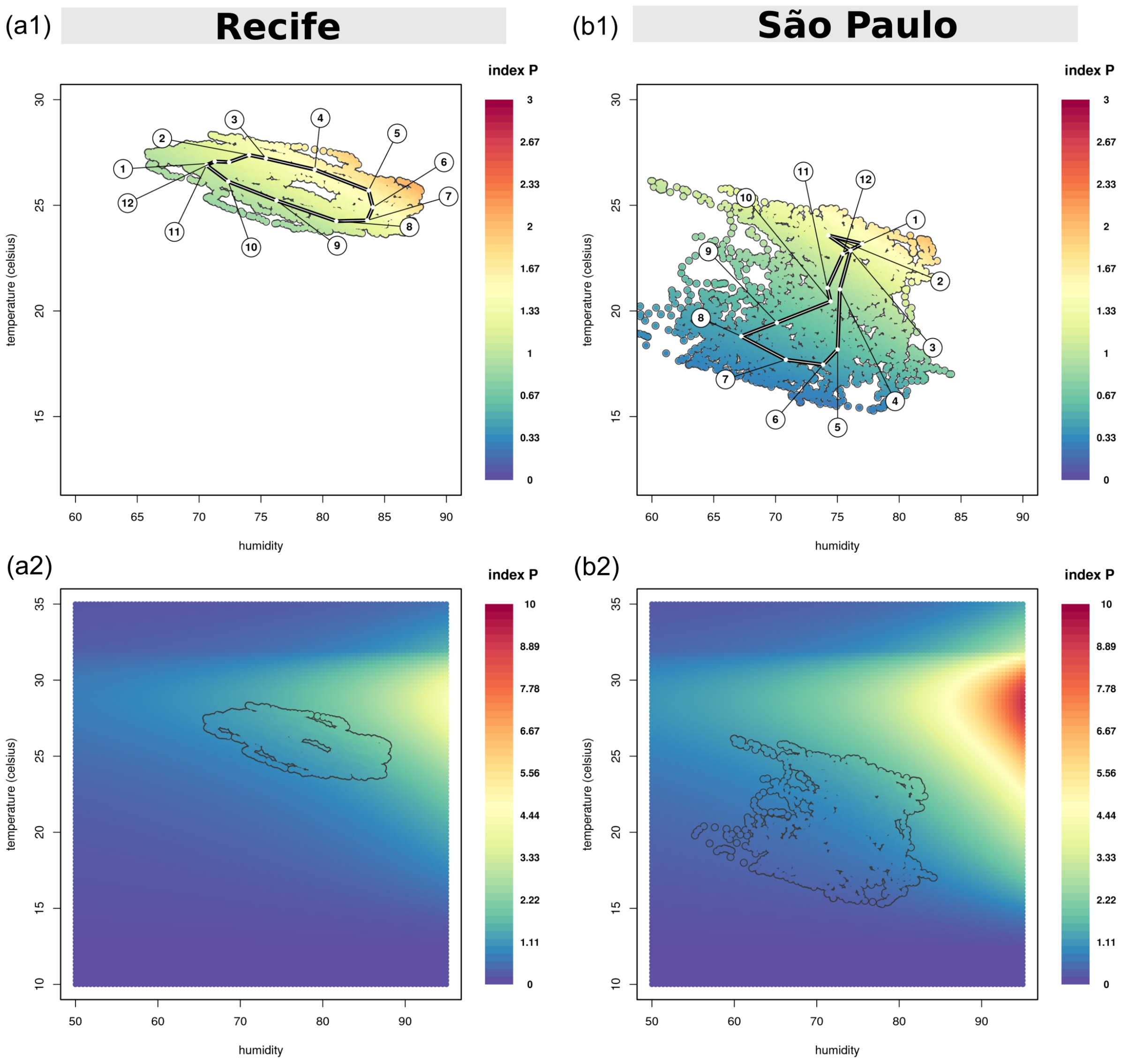

Figure 3: **Heatmaps for empirical and theoretical index P.**
Recife (a1-2) and São Paulo (b1-2). **(a1,b1)** Heatmaps for empirical index P. Each colored point is an observed combination of relative humidity and temperature, colored according to the value of the estimated mean index P (scale on the right). The numbered circles mark the mean temperature and humidity of each month (with January=1 etc). The black and white curve interpolates the means of each month. ***Observations / interpretation:*** *In Recife, the temporal gradient of the index P is clear, with each month appearing on an approximate ellipse. In São Paulo, the temporal gradient of the index P is less clear in the period of October-April, but again follows an approximate ellipse in the May-September period. Note that these Heatmaps are best suited for visualization of mean index P behaviour, rather than characterization of peak times. For the latter purpose, other visualisations may be more useful (see main text).* **(a2,b2)** Heatmaps for empirical index P. Every value of the climatic variables within the ranges 50-95% for humidity and 10-35 Celsius for temperature (step 0.05) are considered and the index P is estimated (colored points, scale on the right). The 'cloud' of points with black borders are the empirical observations of subplots a1 and b1. **MVSE details:** Visual output can be generated with the *plotEmpiricalSuitRespMap()* and *plotTheoreticalSuitRespMap()* functions. Parameters used are the same as in the main text for both regions. Note the different color scales for empirical and theoretical index P.

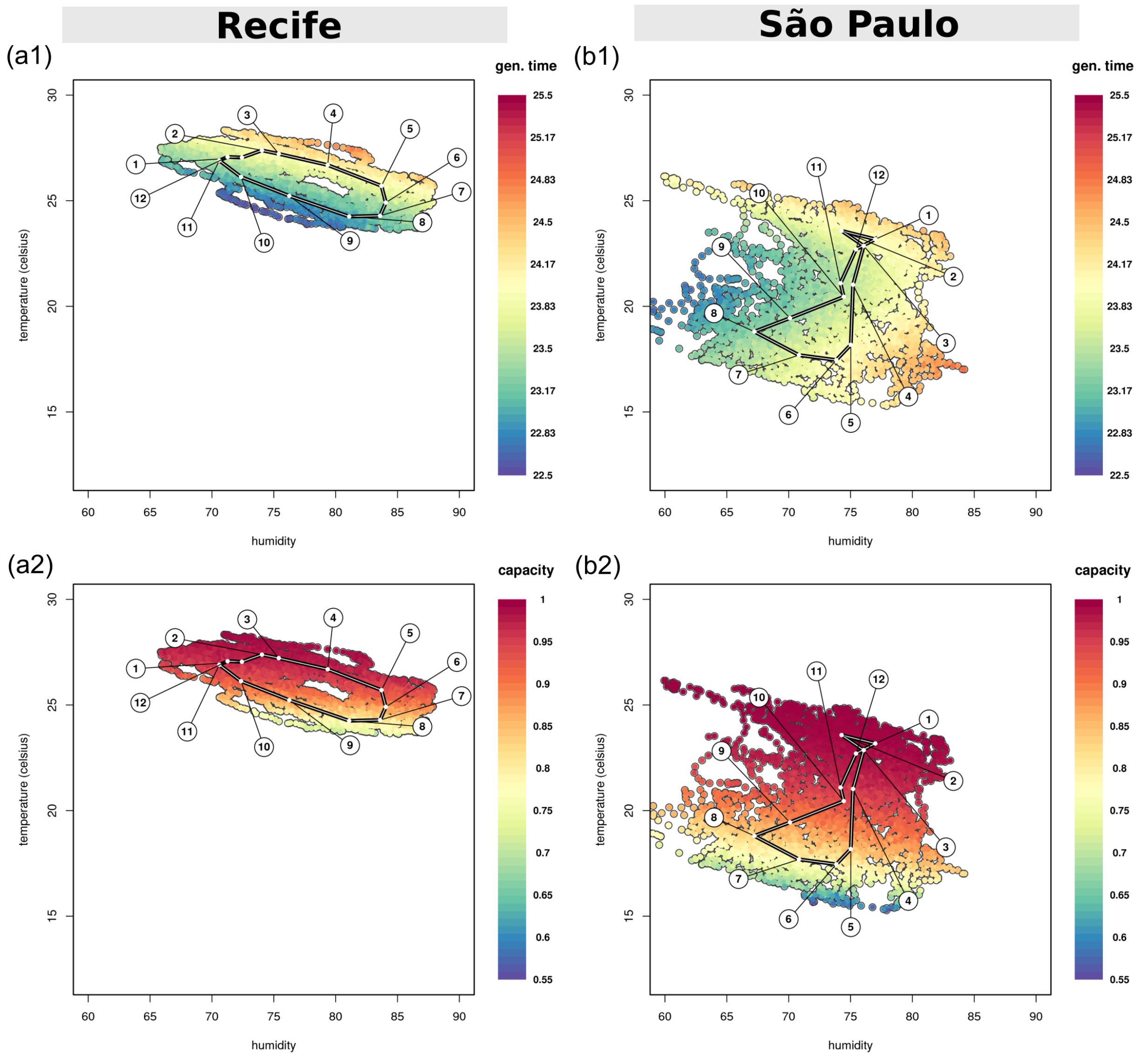

Figure 4: **Heatmaps for generation time and vectorial capacity.**
Recife (a1-2) and São Paulo (b1-2). **(a1,b1)** Heatmaps for the generation time. Each colored point is an observed combination of relative humidity and temperature, colored according to the value of the estimated mean index P (scale on the right). The numbered circles mark the mean temperature and humidity of each month (with January=1 etc). The black and white curve interpolates the means of each month. **(a2,b2)** Heatmaps for vectorial capacity. ***Observations / interpretation:*** *In Recife, the temporal gradient of the generation time is clear, with each month appearing on an approximate ellipse. In São Paulo, the temporal gradient of the index P is less clear in the period of October-April, but but again follows an approximate ellipse in the May-September period. Both higher temperature and humidity seem to be correlated with longer generation times, while only higher temperature dictates higher capacity.* **MVSE details:** Visual output can be generated with functions *plotEmpiricalVecCapMap()* and *plotEmpiricalGenTimeMap()*.Parameters used are the same as in the main text for both regions. Note the different color scales for empirical and theoretical index P.

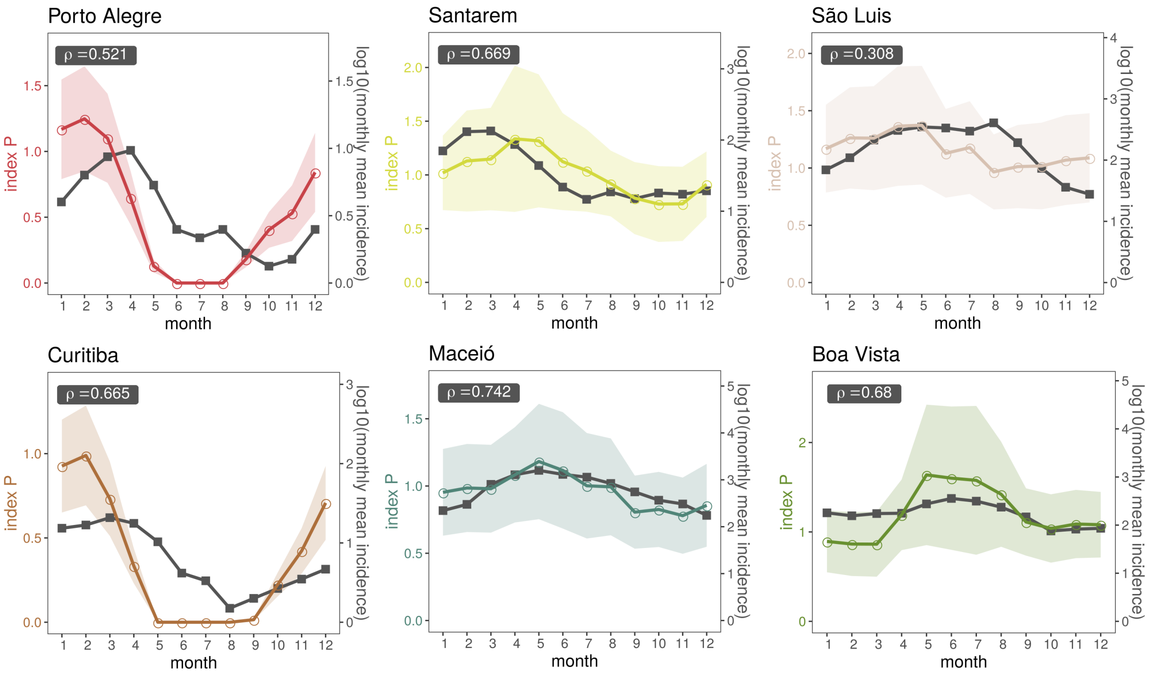

Figure 5: **Pearson's correlation of index P and dengue notifications for 6 cities in Brazil.** These subplots show the index P and mean number of cases per month (2007-2012) for 6 cities of Brazil The shaded areas are the standard deviation of the estimated index P per month. ρ is the Pearson's correlation. This figure complements Figure 4 of the main text.

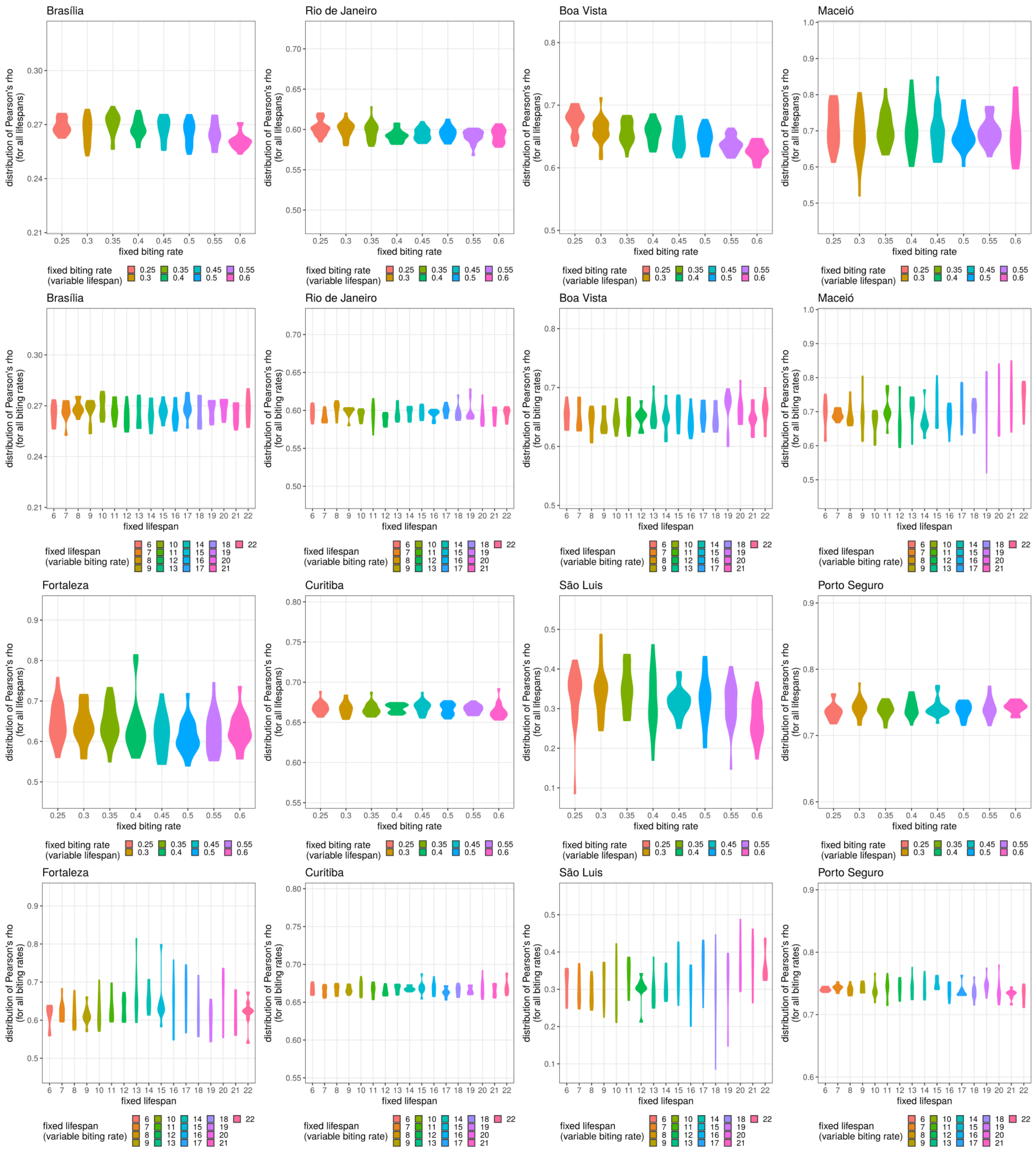

Figure 6: **Sensitivity of Pearson's correlation between index P and dengue notifications for Brasília, Rio de Janeiro, Boa Vista, Maceió, Fortaleza, Curitiba, São Luis, Porto Seguro.** Figure presents two subplots per city (city names on the top left of each subplot). **For each city the top subplot** presents the distribution of Pearson's correlation (rho) when the biting rate is fixed (according to x-axis and colour scale), but the mosquito lifespan is varied. **For each city the bottom subplot** presents the distribution of Pearson's correlation (ρ) when the lifespan is fixed (according to x-axis and colour legends), but the mosquito biting rate is varied. The ranges used for biting rate and lifespan are in the colour legends. ***Observations/interpretation:*** *For most cities, changing the priors does not significantly alter the correlations of P with notifications. Two exceptions (e.g.): Boa Vista and São Luis, which present higher* ρ *for lower bitting rates independently of the lifespan; São Luis also presents higher* ρ *for longer lifespans. This figure is part 1 of a series of 2 Figures (S6-7).*

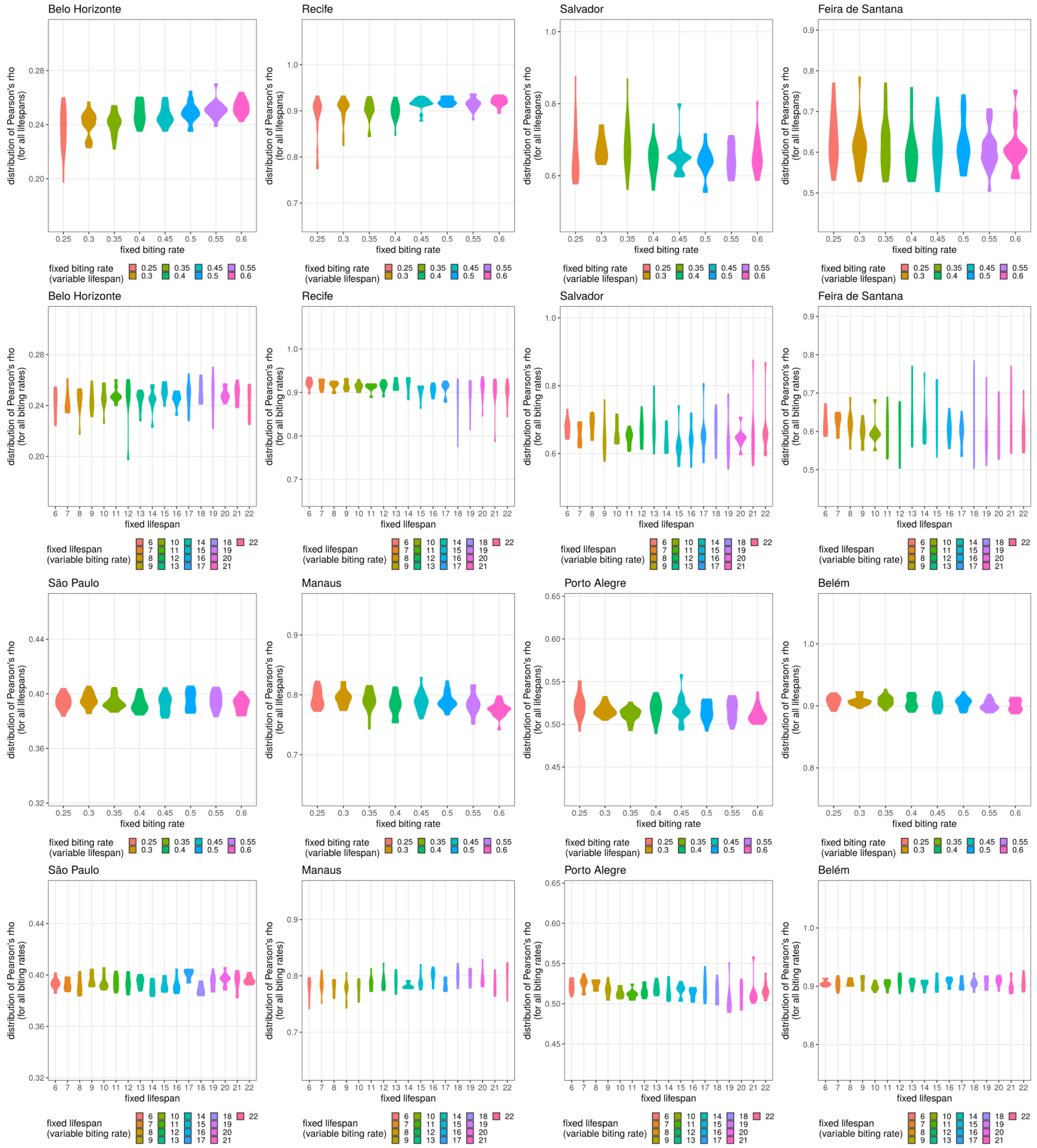

Figure 7: **Sensitivity of Pearson's correlation between index P and dengue notifications for Belo Horizonte, Recife, Salvador, Feira de Santana, São Paulo, Manaus, Porto Alegre, Belém.**
See legend of Figure S6 for details. This figure is part 2 of a series of 2 Figures (S6-7).

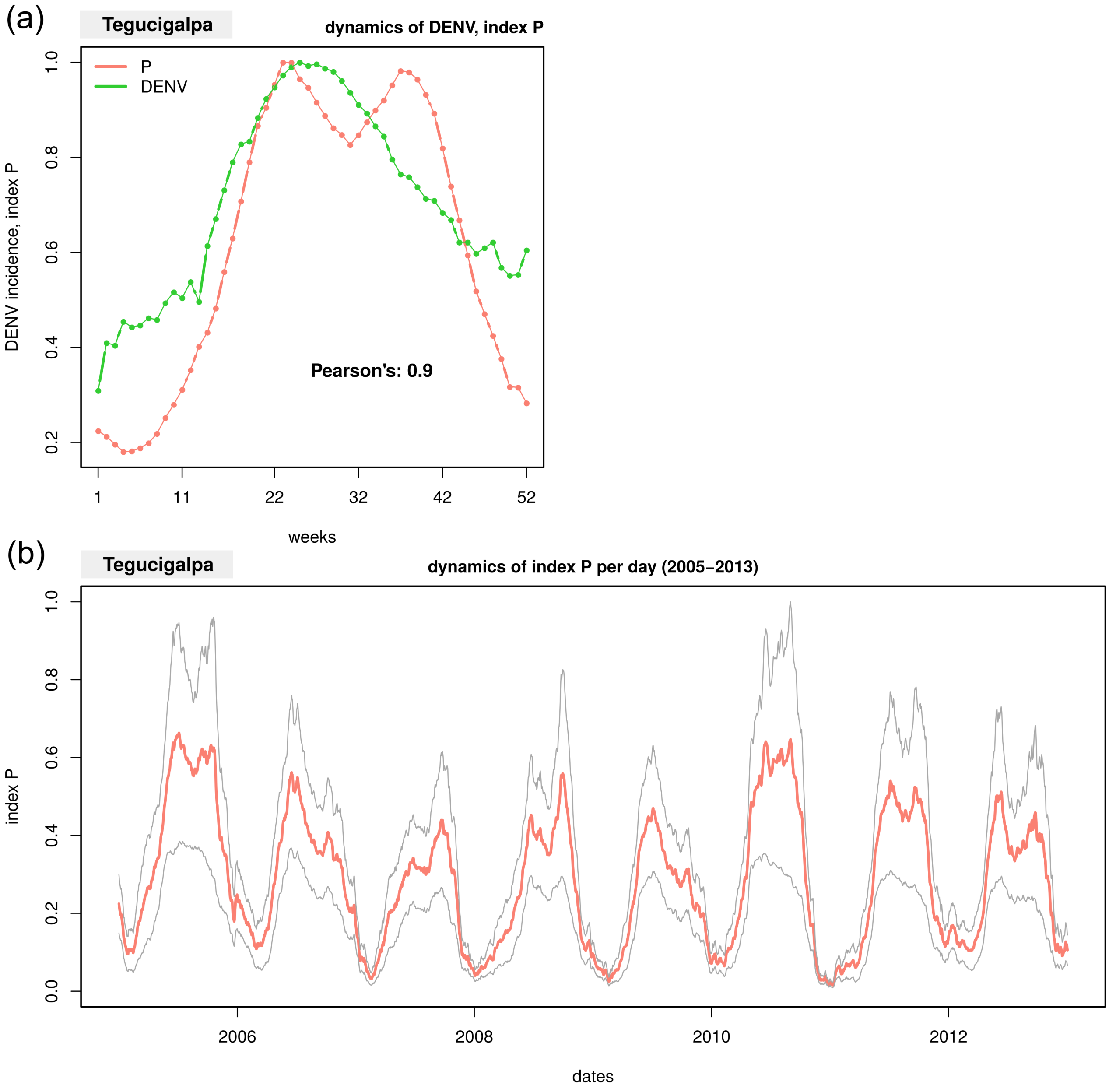

Figure 8: **Index P, DENV incidence and Aedes aegypti suitability score in Tegucigalpa and Honduras at different time scales.**
(a) Normalised index P (red) and *log10* DENV incidence (green) per week (normalised by maximum value in each time series). Index P at each week is the mean across the same week in the period 2005-2013; effectively presenting the index P for 'typical weeks' based on past climate data. The resulting Pearson's correlation is presented within the panel. **(b)** Daily time series of estimated index P and 95% CI (grey) between 2005 and 2013. ***Observations/interpretation:*** *This figure complements figure 5 in the main text. It shows that: the index P can be estimated / used at higher temporal resolution when using local climate data from weather stations, and that P and AaS behave similarly at the country level.*
