# Supplementary material for: MVSE: An R‐package that estimates a climate‐driven mosquito‐borne viral suitability index
Source: Methods Ecol Evol. 2019 Jun 19;10(8):1357–70. doi: 10.1111/2041-210X.13205 (PMC7202302; doi:10.1111/2041-210X.13205)
Supplement: Supplementary file 1 [file MEE3-10-1357-s001.zip › mee313205-sup-0004-TextS2.pdf]

MVSE: an R-package that estimates a climate-driven mosquito-borne viral suitability index.

Full methods and data description.

January 7, 2019

Contents

1 MVSE detailed methods 2

1.1 Climate-driven functions for ento-epidemiological parameters . . . 2

1.2 Estimation of the index P . . . 3

1.3 Priors used for the results presented . . . 5

2 MVSE demonstrations 5

2.1 Estimation of index P accross South America . . . 5

2.2 Estimation of index P across Brazil . . . 6

3 MVSE implementation, functionality and outputs 7

3.1 Climate data importation . . . 7

3.2 Setting the framework's parameters . . . 8

3.3 Ecological scaling factors (α, η, ρ) and empirical index P estimation . . . 8

3.4 Index P trend filtering . . . 8

3.5 Index P peak timing . . . 9

3.6 Seasonal timing and length . . . 9

3.7 Empirical versus theoretical index P . . . 9

3.8 Generation time . . . 10

3.9 Vectorial capacity . . . 10

3.10 Graphical outputs . . . 10

3.11 Data outputs . . . 13

3.12 Assumptions on missing climatic data . . . 13

3.13 Minimal code example . . . 14

1 MVSE detailed methods

1.1 Climate-driven functions for ento-epidemiological parameters

Climate-dependent expressions exists for all of the ento-epidemiological parameters used to calculate index P (equation 3, main text). These expressions are the same as those used in our previous modelling studies for DENV and ZIKV [1, 2, 3].

For temperature (t in Celsius, tk in Kelvin), we use polynomial expressions taken from previous studies fitting laboratory-estimated entomological measurements to temperature. Adult vector mortality (μ^v) was modelled by a polynomial estimated in a study by Yang et al. under temperature-controlled experiments on populations of *Aedes aegypti* (equation 1) [4]. Extrinsic incubation period ($1/\gamma^v$) was modelled as a function of temperature, based on the formulation by Focks et al., which assumes that replication is determined by a single rate-controlling enzyme [5, 6, 7] (equation 2, were R is the universal gas constant in $cal\ deg^{-1}\ mol^{-1}$). The probability of transmission per mosquito bite ($\phi^{v\to h}$) was modelled (equation 3) as estimated by Lambrechts et al. [8].

$$\breve{\mu}^v_{(t)} = 0.8692 - 0.1599t + 0.01116t^2 - 0.0003408t^3 + 0.000003809t^4 \quad (1)$$

$$\breve{\gamma}^v_{(t)} = \frac{0.003359\frac{tk}{298} \times \exp(\frac{15000}{R}(\frac{1}{298} - \frac{1}{tk}))}{1 + \exp(\frac{6.203\times10^{21}}{R}(\frac{1}{-2.176\times10^{30}} - \frac{1}{tk}))} \quad (2)$$

$$\breve{\phi}^{v\to h}_{(t)} = 0.001044t \times (t - 12.286) \times (32.461 - t)^{1/2} \quad (3)$$

The relationships between humidity (u) and entomological parameters are introduced using the expressions below (equations 4, 5) [3]. We normalise the time series of humidity to [0,1], and use the mean normalised values ($\bar{u}$) as reference for extreme deviations from the expected local tendencies [9, 10]. Humidity is assumed to increase the vector biting rate (a^v) [11] while decreasing the adult mosquito mortality rate (μ^v) [12].

$$\breve{a}^v_{(u)} = (u - \bar{u})/\sqrt{1 + (u - \bar{u})^2} \quad (4)$$

$$\breve{\mu}^v_{(u)} = \bar{u} - (u - \bar{u})/\sqrt{1 + (u - \bar{u})^2} \quad (5)$$

Finally, we combine the effects of humidity and temperature to get the complete expressions for the entomological parameters:

$$\mu^v_{(u,t)} = \eta\breve{\mu}^v_{(t)}[1 + \breve{\mu}^v_{(u)}]^\rho \quad (6)$$

$$\gamma^v_{(t)} = \alpha\breve{\gamma}^v_{(t)} \quad (7)$$

$$\phi^{v\to h}_{(t)} = \breve{\phi}^{v\to h}_{(t)} \quad (8)$$

$$a^v_{(u)} = a[1 + \breve{a}^v_{(u)}]^\rho \quad (9)$$

These expressions include the *multiplicative coefficients* (α, η, ρ) to which we generally refer to as *scaling ecological factors.* The inclusion of α and η does not alter the relative effect of climate variation on the entomological parameters (as determined in other studies [4, 5, 6, 7]), but allows for the parameters' baselines to be different from the ideal laboratory conditions of the original research (e.g. [4]). In practice, the effect of temperature on these parameters can be considered to be the same as observed under laboratory conditions if $\eta, \alpha \approx 1$; weaker if η, α <1; and stronger if η, α >1. The exponential coefficient ρ allows instead to modulate the strength by which adult mosquito mortality and biting rate

react to deviations from local mean humidity. In practice, the effect of humidity is switched off when $\rho \approx 0$ and made stronger when $\rho > 1$. For a discussion on possible biological conditions that may justify these factors' divergence from 1, please refer to the original description of the method [1] and to a separate study from Brady and et al. [13].

1.2 Estimation of the index P

MVSE uses prior knowledge about the ento-epidemiological parameters that compose the index P expression (equation 3, main text) to estimate the index, through time, in three essential steps (Figure 1):

Step 1 - setting informed priors

The expression of the index P (main text, equation 3) depends on eight parameters, four are climate-dependent and four and climate-independent. MVSE sets informed priors (mean, standard deviation, type of distribution) for all parameters. For simplicity, these priors are assumed to remain unchanged for the entirety of the time range under study. For example, if a time range of three years is studied, it is assumed that a prior for mosquito life-span with a mean of 12 days and a standard deviation of 3 days is representative of the entire time period, including the potential natural oscillations in life-span between winter and summer months (i.e. in and off season).

The climate-independent parameters are: the human life-span ($1/\mu^h$), the transmission probability from infected human to mosquito per bite ($\phi^{h\to v}$), the human incubation period ($1/\sigma^h$), and the human latency period ($1/\gamma^h$). The climate-dependent parameters are: the life-span of adult mosquitoes ($\mu^v_{(u,t)}$, equation 6), the extrinsic incubation period ($1/\gamma^v_{(t)}$, equation 7), the daily biting rate ($a^v_{(u)}$, equation 9) and the probability of transmission from an infected mosquito to a human ($\phi^{v\to h}_{(t)}$, equation 8).

Step 2 - estimating posteriors for the scaling ecological factors

MVSE implements a Bayesian Markov chain Monte Carlo (bMCMC) to explore the parameter space of η and ρ which takes into consideration the priors of $a^v_{(u)}$ and $\mu^v_{(u,t)}$ (equations 6, 9). Independently and in parallel, it explores the parameter space of α through sampling that considers the prior of $\gamma^v_{(t)}$ (equation 7).

In effect, for every combination of α, η and ρ sampled by the bMCMC, equations 6, 7, 9 are used to estimate the climate-driven time series of $\gamma^v_{(t)}$, $a^v_{(u)}$ and $\mu^v_{(u,t)}$. The likelihood that these time series represent the proposed priors, given the parameter combination α, η and ρ, is assessed through the product of the conditional probabilities of each time point of $\gamma^v_{(t)}$, $a^v_{(u)}$ and $\mu^v_{(u,t)}$ with the prior probabilities of those parameters. The conditional probabilities are calculated according to the type of distribution set on the priors. The result of running the bMCMC is the estimation of posteriors for α, η and ρ.

Step 3 - prior, posterior sampling and estimation of index P

For each time point in the climatic data series, N samples are drawn from the priors and posteriors that compose the expression of the index P (equation 3, mains text). That is, N points are drawn from the informative priors of parameters (independent of the scaling ecological factors) for each of the parameters $1/\mu^h, \phi^{h\to v}, 1/\sigma^h$, $1/\gamma^h, \phi^{v\to h}$; and N samples are obtained from the posteriors of each of the scaling factors; then, these are plugged into equations 6, 7, 9 to obtain N values of each of the parameters $\gamma^v_{(t)}$, $a^v_{(u)}$ and $\mu^v_{(u,t)}$ (given humidity and temperature at that time point). Finally, these parameter values

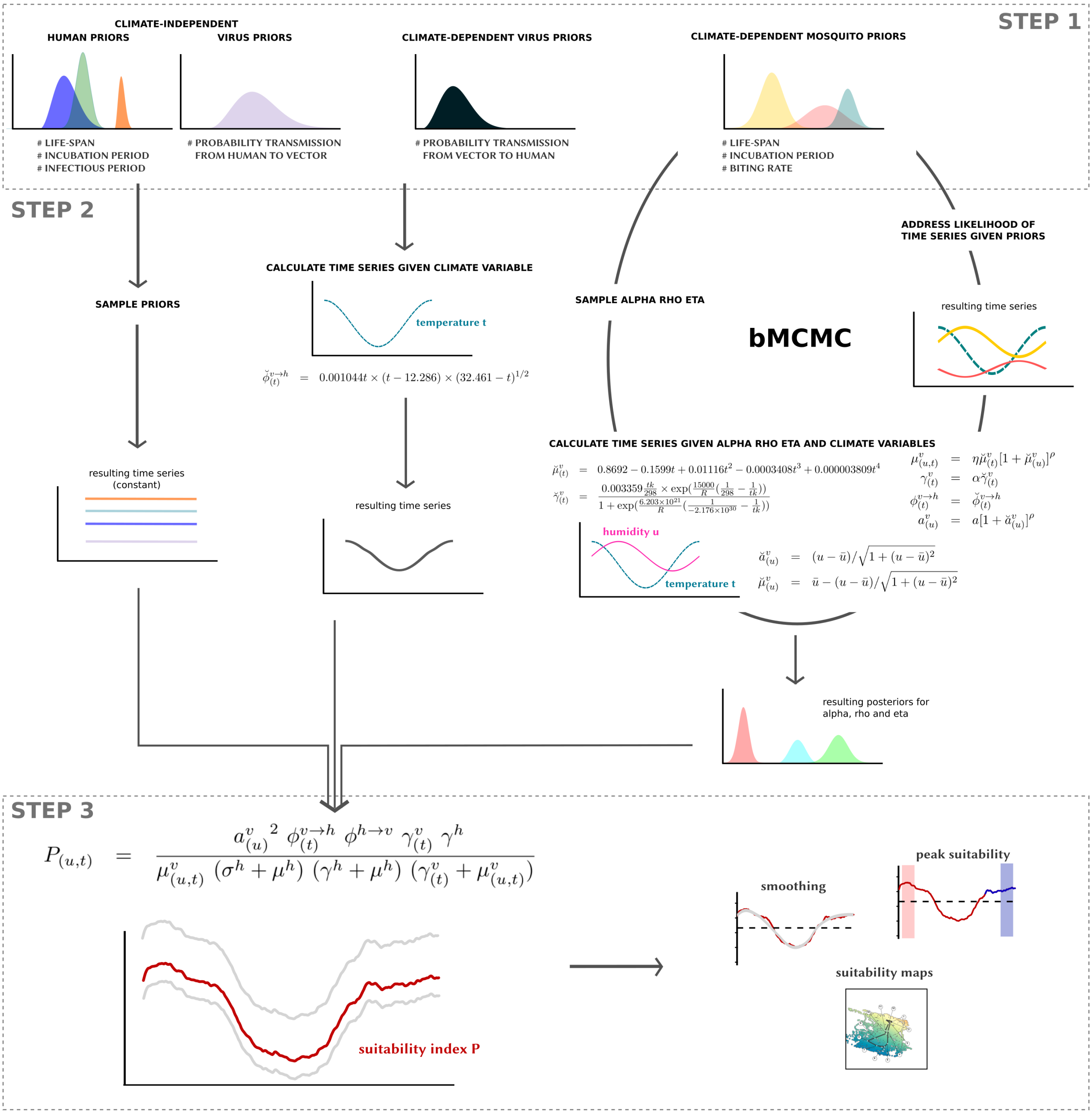

$$P_{(u,t)} = \frac{{a^v_{(u)}}^2 \ \phi^{v\rightarrow h}_{(t)} \ \phi^{h\rightarrow v} \ \gamma^v_{(t)} \ \gamma^h}{\mu^v_{(u,t)} \ (\sigma^h + \mu^h) \ (\gamma^h + \mu^h) \ (\gamma^v_{(t)} + \mu^v_{(u,t)})}$$

Figure 1: **Diagram of steps taken to estimate the index P.**

Name	symbol	mean	stdev.	refs.
adult mosquito life-span	$1/\mu^v_{(u,t)}$	12 days	2	[14, 15]
mosquito incubation period	$1/\gamma^v_{(t)}$	7 days	2	[16, 17, 18]
mosquito biting rate	$a^v_{(u)}$	0.25 / day	0.01	[11, 19]
human life-span	$1/\mu^h$	71 years	2	—
human incubation period	$1/\gamma^h$	5.8 days	1	[3, 16, 20]
human infectious period	$1/\sigma^h$	5.9 days	1	[3, 16, 20]
human to mosquito probability of transmission	$\phi^{h\to v}$	0.5	0.01	—-

Table 1: Prior distributions used.

are plugged into the index P expression (equation 3, main text), and summary statistics of P (mean, standard deviation and confidence interval) are extracted per time point.

1.3 Priors used for the results presented

Results presented in both the main text and supplementary material used the same informed priors (unless stated otherwise). We used gamma distributed priors (although Gaussian priors produce very similar results, not shown). Priors were generally based on literature sources regarding *Aedes* mosquitoes and DENV / ZIKV viruses, and when unavailable, our past experience with the mathematical model from which the index P is derived. Table 1 summarizes the priors.

2 MVSE demonstrations

2.1 Estimation of index P accross South America

We used WorldClim (version 2) global data to estimate the index P across South America using the MVSE software package (Figure 2, [21]). This data set, updated in July 2016, contains, among other variables, average monthly climate data for mean temperature and water vapor pressure (kPa), for the period of 1970-2000, and is publicly available (http://worldclim.org/version2). The spatial resolution used was of 10 minutes ($\approx 340km^2$). Figure 2 presents examples of the two mentioned variables in January and June.

We select the region of South America by truncating the world data to locations between rows 470 and 850, and between columns 590 and 880. MVSE requires relative humidity (RH) instead of water vapour pressure (WVP), which can be obtained using the expressions below, with Pws as the water vapour *saturation pressure*, WVP converted to hPa, and the constants A=6.116441, m=7.591386 and Tn=240.7263 (valid for temperature ranges of [-20,50] Celsius up to an error of 0.083% (Vaisala 2013):

$$RH = 100 \times \frac{WVP}{Pws} \quad (10)$$

$$Pws = A \times 10^{\frac{m \times Tc}{Tc+Tn}} \quad (11)$$

After transformation of WVP into RH, the WorldClim data is effectively a grid of raster pixels, with 12 points of temperature and relative humidity per pixel (a 3D array). Given the resolution of the data, each pixel represents a square with edges of $\approx$ 18 Km. It is thus possible to apply MVSE's Bayesian approach to estimate index P per pixel over the 12 months. We ran MVSE for 110,200 pixels

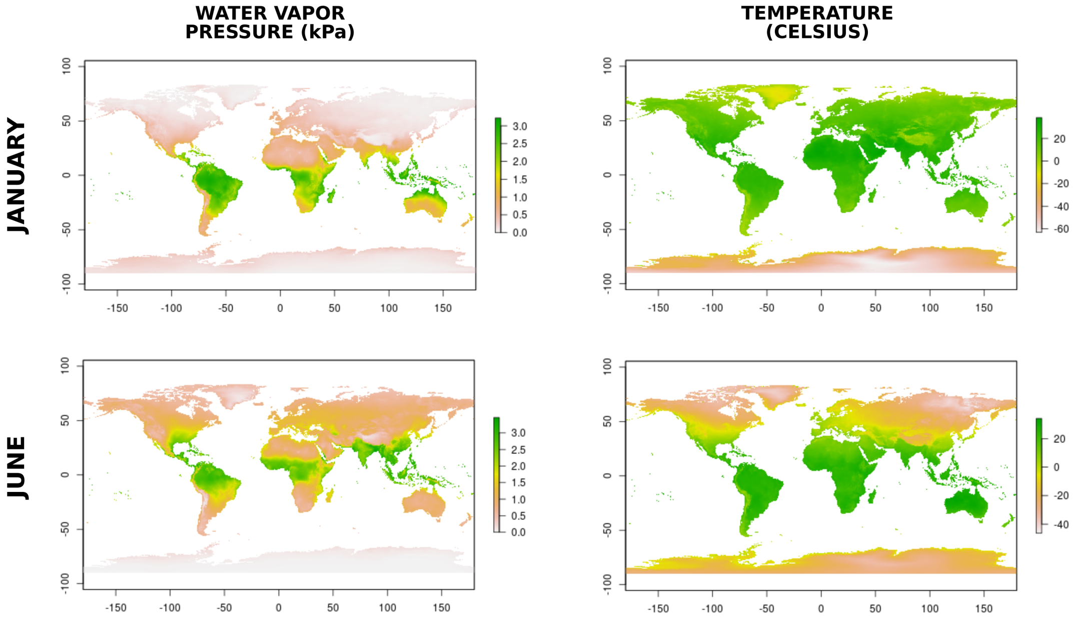

Figure 2: **Examples of WorldClim V2 data.**
(left) Water vapour pressure (in kPa) for January and June, coloured according to the scale on the right. **(right)** Temperature (in Celsius) for January and June, coloured according to the scale on the right.

corresponding to the region of South America using the same human, viral and mosquito parameters as for Recife and São Paulo in all the presented results of the main text. We ran each MCMC for 1 million steps. The estimated index P was based on an average of N=1000 simulations.

The resulting output was index P estimated over 12 points (months) per pixel. As supplementary material, we include a multi-page PDF file with the spatio-temporal frames over the 12 months for mean index P and an Rdata file with the solution. The later is a list of 12 entries (frames representing Jan - Dec), each with a matrix (380x290) for index P. Frames can be visualised using the R base function *image()*.

Results are summarized in the maps of Figure 3. Mosquito-borne viral suitability is estimated to be highest, on a yearly average, in the centre of Brazil, Colombia and Venezuela - countries which are known to be endemic to DENV, CHIKV and ZIKV [22, 23, 24, 25, 26, 3, 27]. Suitability is also high along the northern coast of the continent and the Caribbean Sea, an areas believed to be niches for arboviral persistence [22, 23]. In contrast, suitability decreases from north to south, with the lowest values in Chile, Argentina and Uruguay. Chile and Uruguay are the only South American countries which are known to not have indigenous dengue transmission, and Argentina is known to present very low incidence [27, 28]. Notably, index P is mostly zero along the *highlands* of the continent (Andes), where climate is expected to unsuitable for *Aedes* [29].

2.2 Estimation of index P across Brazil

A shapefile of Brazil was used to extract the region of the country from the WorldClim (version 2) global data [21]. Once this (sub)grid of raster pixels was obtained, the same approach as explained above was used for South America. The results for Brazil are in the main text. We also make available

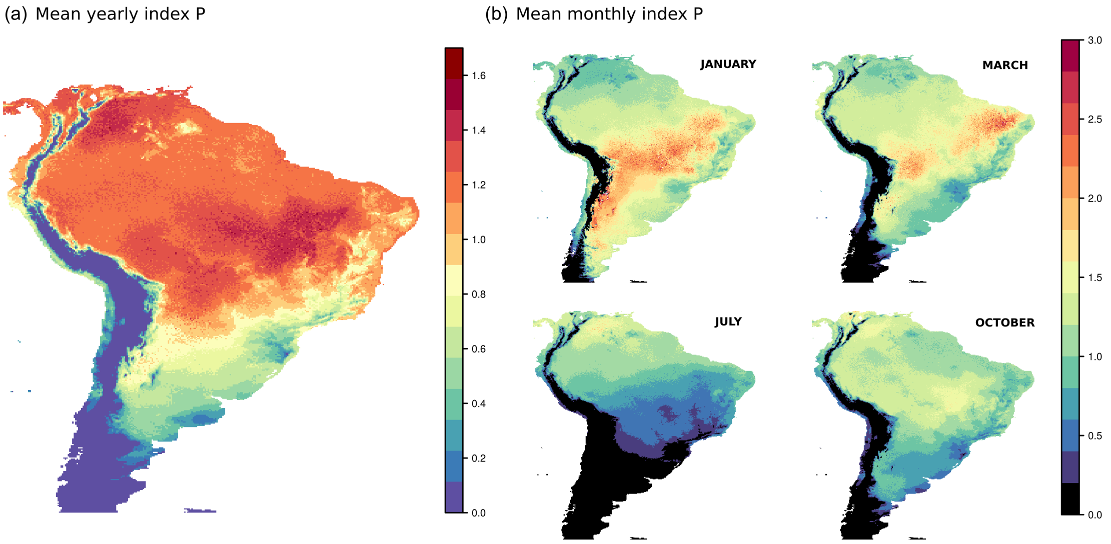

Figure 3: **South American maps for mean index P per year and month.**
(a) Map for mean yearly index P. Each colored pixel ($\approx 340km^2$) is the mean index P of the 12 (months) data points in the data set WorldClim (version 2) (see supplementary full methods and data description). The color scale on the right is the estimated range of index P values. **(b)** Maps for mean monthly index P. Each pixel is colored in the same way as in (a). **MVSE details:** There is no function to analyse or explore the WorldClim (version 2) data set, since it can not be redistributed. The estimations per month are made available in supplementary files.

as supplementary material a multi-page PDF file with the spatio-temporal frames and Rdata file with solutions.

3 MVSE implementation, functionality and outputs

The MVSE package was implemented in R, and contains a set of functions that produce estimation, interpretation and exportation of the index P. In the main text, we present graphical outputs for some of the implemented functions which support the use of the index P as a measure of mosquito-borne viral suitability.

Here, we offer a full description and exemplification of MVSE's functionalities, as well as code examples. For simplicity, we do not present a full description of all the parameters available for each implemented function. Further details on such optional parameters are available in MVSE's manual (included with the source code of the package).

3.1 Climate data importation

The function *setEmpiricalClimateSeries* is available for the importation of climatic time series from comma-separated values (CSV) files. The format requirements are 3 numeric columns: T, H, date; for temperature, humidity and date, respectively. Temperature is expected in Celsius, (relative) humidity in the range 0 - 100 (%) and date in the format YYYY-MM-DD. MVSE allows for the time intervals in the climatic series to be of any scale (e.g. days, weeks). Some functions allow for missing time points (see *Assumptions on missing data* below).

```
setEmpiricalClimateSeries(filepath="TableSz_climate_SaoPaulo.csv)
```

3.2 Setting the framework's parameters

Seven functions are available for setting the prior distributions (mean, standard deviation, type of distribution) for the framework's parameters (see expression 3 in main text).

In the context of entomological parameters, the function *setMosqLifeExpPrior* sets the prior for the adult life-span, *setMosqIncPerPrior* sets the prior for the extrinsic incubation period, and *setMosqBitingPrior* sets the prior for the biting rate. Values are expected in the scale of days.

```
setMosqLifeExpPrior(pmean=14, psd=3, pdist='gaussian')
setMosqIncPerPrior(pmean=7, psd=2, pdist='gaussian')
setMosqBitingPrior(pmean=0.25, psd=0.05, pdist='gaussian')
```

For the human parameters, *setHumanLifeExpPrior* sets the prior for life-span, *setHumanIncPerPrior* sets the prior for the incubation period, *setHumanInfPerPrior* sets the prior for the infectious period, and *setHumanMosqTransProbPrior* sets the prior for the transmission probability per bite from infectious human to susceptible mosquito. Values are expected to be in years for life-span, days for the infection and incubation periods, and between 0 and 1 for transmission.

```
setHumanLifeExpPrior(pmean=priorHumanLS, psd=2, pdist='gaussian')
setHumanIncPerPrior(pmean=5, psd=1, pdist='gaussian')
setHumanInfPerPrior(pmean=5, psd=1, pdist='gaussian')
setHumanMosqTransProbPrior(pmean=0.5, psd=0.01, pdist='gaussian')
```

3.3 Ecological scaling factors (α, η, ρ) and empirical index P estimation

After importing the climatic data and incorporating prior knowledge on the framework's parameters, the ecological scaling factors α, η and ρ can be estimated.

For this, the function *estimateEcoCoefficients* allows the user to set Markov chain Monte Carlo (MCMC) parameters (number of steps and burn-in) as well as initial guesses for the coefficients η and ρ and sample (jump) step size. No parameters of this function (except nMCMC dictating number of samples) are related to α, since it is estimated directly from sampling priors. The result of this function call is the creation of a global variable (*MVSE_results*) which contains the posterior distributions for the coefficients.

```
estimateEcoCoefficients(nMCMC=1000000, bMCMC=0.5,
                        cRho=0.1, cEta=1, gauJump=0.75)
```

```
simulateEmpiricalIndexP(nSample=1000)
```

The mean index P and the 95% confidence interval (CI) obtained per time step are added to the global variable *MVSE_results*. The simulated index P series (N= *nSample*) are also saved in a global variable *MVSE_indexP* which is a matrix of size N x T, where T is the number of time steps originally included in the climatic time series.

3.4 Index P trend filtering

Climate variables can be very noisy and thus yield a highly fluctuating estimate of the index P series. Therefore, a desired output might be a smoothed version of the index P.

The function *expectedPosterior* was added to achieve smoothed estimates of the expected values of index P. The function applies a quadratic trend filter to each of the posterior index P series stored in the variable *MVSE_indexP*. The filtered series are subsequently averaged to create a single time series output. The function expects a numeric matrix of simulated index P values, with rows as samples,

in addition to a vector of dates corresponding to sampling times. The dates should be equally spaced and missing values should be imputed, if necessary, beforehand. This method fits piecewise quadratic polynomials to the data, using least-squares estimation with penalization for the fit's smoothness set by a regularization parameter. The regularization parameter is determined via cross validation with the 1-SE rule [30], using a 10-fold cross validation. The function can be applied by the user on the entire time range of the input climatic series or per year (in case the input spans multiple years) by setting the parameter *breakyears* to TRUE or FALSE. This method was implemented using the *genlasso* function in the R package with the same name [31].

The output of this function is also added to the global variable *MVSE_results*.

```
expectedPosterior(indexP, dates, breakyears=FALSE)
```

3.5 Index P peak timing

Another variable of interest might be the time when suitability peaks. The *distributionPeak* function estimates the peak times of index P based on the maximum values from simulated index P series. The function uses the same input as *expectedPosterior*, to create a histogram of peak times, with colors assigned according to peaks estimated in different years. A list containing peak time estimates and confidence intervals is also produced. The output of this function is added to the global variable *MVSE_results*.

```
distributionPeak(indexP, dates)
```

3.6 Seasonal timing and length

Complementary to the time of peak suitability, the length of a season might be of interest. The function *suitableSeason* allows the user to estimate when the index P increases and remains above a user-defined threshold, set by the parameter Pthreshold. To avoid noisy estimates of seasonality, the user can also define a time period for which dipping below the threshold will not disrupt the estimate of seasonality, set by the timethreshold parameter (e.g. timethreshold=1 will not consider a single-day dip below Pthreshold, followed by a return above it, as 'breaking' the season). The function accepts a vector of index P estimates (averaged or filtered) across multiple years, and outputs a plot of the seasons when index P is above the defined P threshold, as well a list of the relevant values.

```
suitableSeason(indexP, dates, timethreshold=0, Pthreshold=1)
```

3.7 Empirical versus theoretical index P

MVSE offers the possibility of estimating index P for a range of theoretical climatic values. The priors and posteriors used are the ones obtained when estimating the empirical index P (see above). For this, a call to *setTheoreticalClimateSeries* is required to set the desired climatic ranges, followed by a call to *simulateTheoreticalIndexP*, which estimates the index P for every combination of temperature and humidity values set on the first call.

```
setTheoreticalClimateSeries(Trange=seq(10,35,length.out=100),
                            Hrange=seq(50,95,length.out=100))
simulateTheoreticalIndexP(nSample=1000)
```

The output is saved in a similar manner to the empirical index P. The mean theoretical index P and the 95% confidence interval (CI) of the ranges obtained per time step are added to the global variable *MVSE_results*. The simulated theoretical index P series (N= *nSample*) are also saved in a global variable

MVSE_indexP_theoretical which is a matrix of size N x T, where T is the number of time steps originally included in the climatic time series.

3.8 Generation time

We take the *generation time* (GT) as the expected time for one human case to transmit the virus to a vector and then to another human. The function *simulateGenerationTime* samples (N) the estimated posteriors of the vector lifespan and incubation period, together with the priors for human infectious period and incubation period, to achieve a posterior distribution of GT.

```
simulateGenerationTime(N=200)
```

GT is the sum of time of two processes: the time it takes a human to transmit the virus to a vector (incubation + *effective infectious waiting time*), and the time it takes the vector to transmit the virus to a second human (incubation + *effective infectious waiting time*). It is assumed that transmission may take place at any time point during the infectious period of the human and the vector, where the mosquito's infectious period is the incubation period subtracted from the lifespan. That is, for simplicity, we assume the *effective infectious waiting time* to be uniformly distributed (i.e. if an infectious period is X days, we sample from a uniform distribution between 0 and X days to determine the time of transmission).

The mean GT and the 95% confidence interval (CI) of the ranges obtained per time step are added to the global variable *MVSE_results*.

3.9 Vectorial capacity

We define vectorial capacity (VC) as the fraction of time (between 0 and 1) in which the mosquito lifespan is estimated to be longer than the incubation period. VC is estimated using the above described function *simulateGenerationTime*. The mean VC and the 95% confidence interval (CI) of the ranges obtained per time step are added to the global variable *MVSE_results*.

3.10 Graphical outputs

All graphical examples presented in main text (except for the geographical maps) are produced by functions available to the user. Below are some examples of function calls for visual output (which assume estimations and analyses have already been done beforehand, as described in the sections above). Some of these functions yield a larger number of visualisations with complementary outputs, which are not presented here. All outputs are in PDF.

```
plotClimate(outfilename='climate')
```

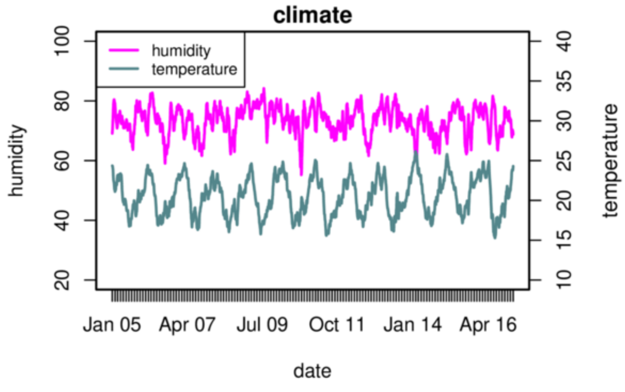

```r
plotEntoParameters(outfilename='ento',entoPostLim=c(0,30),
                   bitPostLim=c(0,1))
```

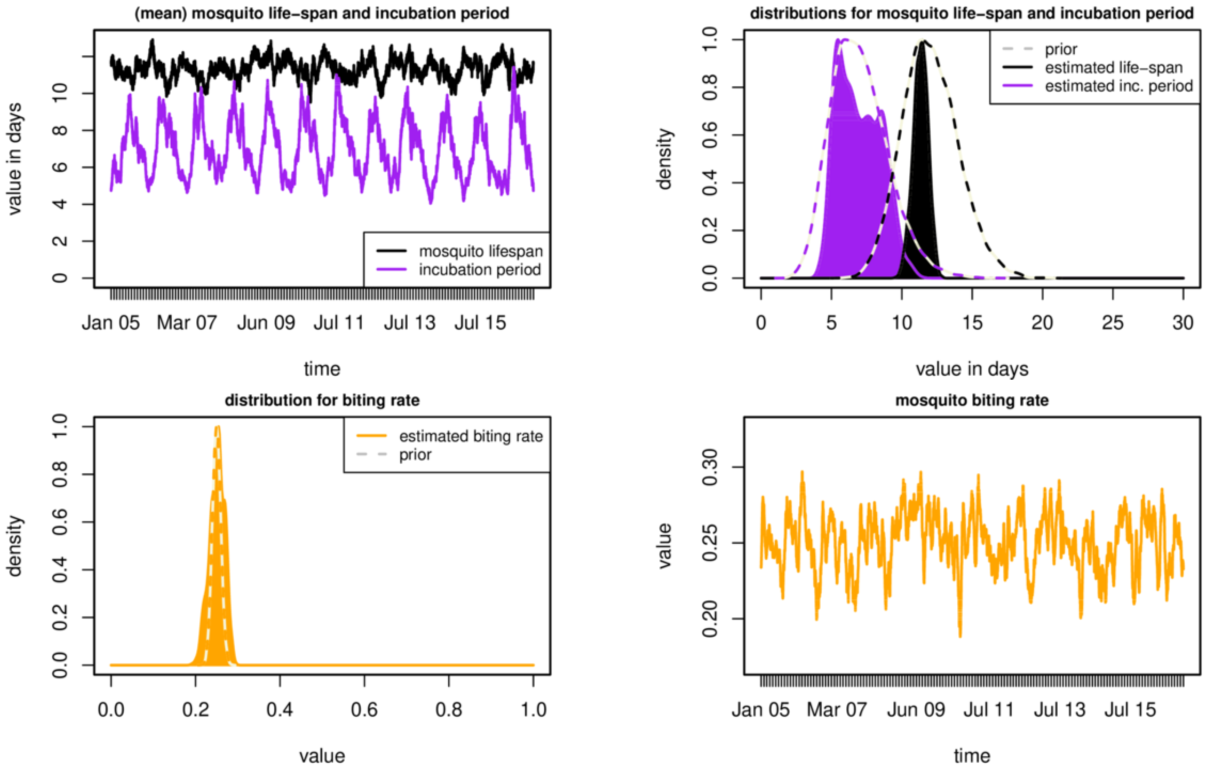


```r
plotEcoCoefPosteriors(outfilename='dist', etaLim=c(0,12),
                      alphaLim=c(0,12), rhoLim=c(0,12))
```

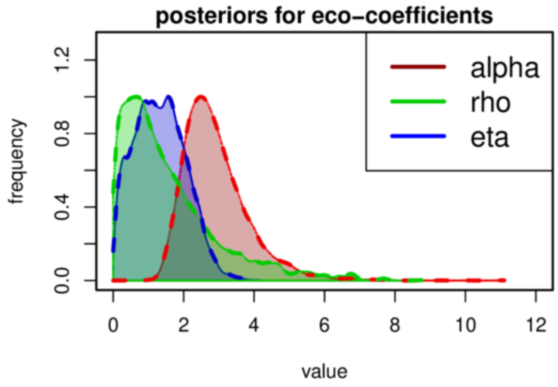


```r
plotEmpiricalIndexP(outfilename='indexP')
```

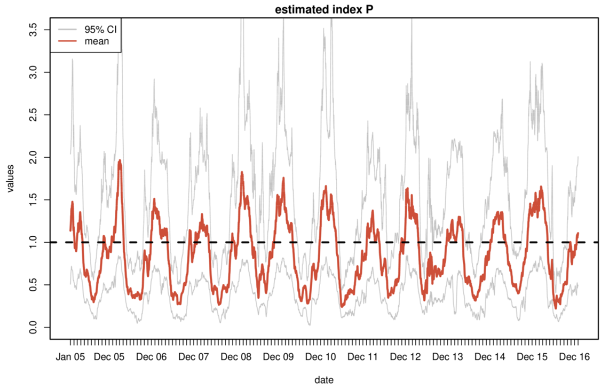


```
plotGenerationTimes(entoLim=c(0,20),
                    humLim=c(0,10),
                    genLim=c(0,30))
```

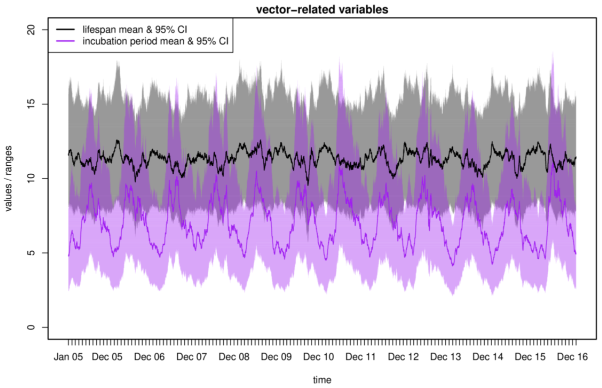

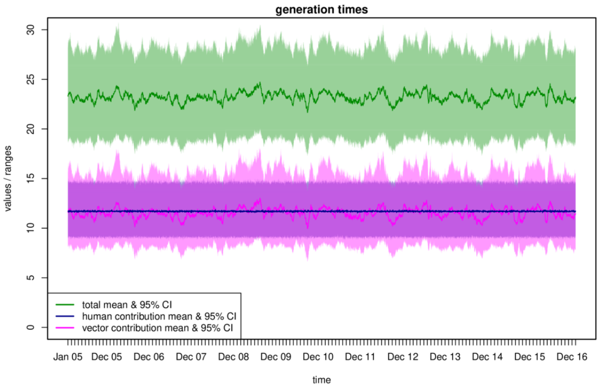

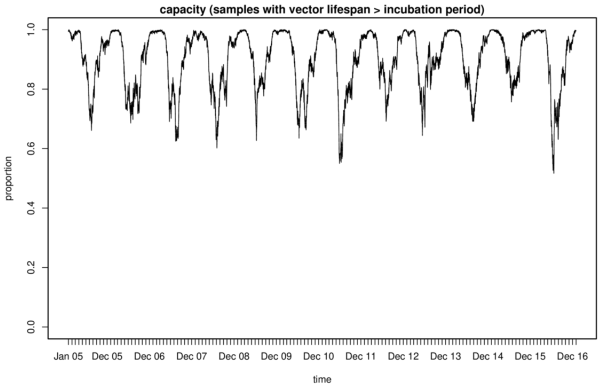

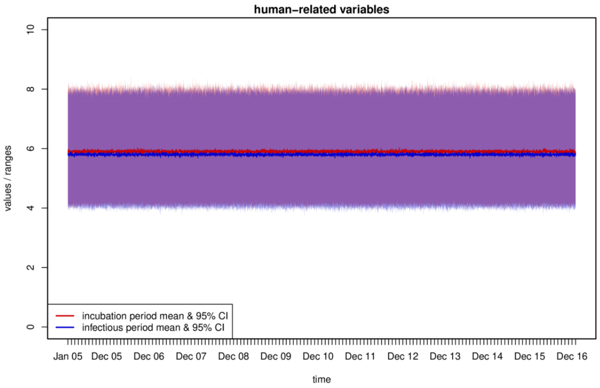


```
plotEmpiricalGenTimeMap(outfilename='empirical_GT',
                        TempLim=c(12,30),
                        HumLim=c(60,90))

plotEmpiricalVecCapMap(outfilename='empirical_VC',
                       TempLim=c(12,30),
                       HumLim=c(60,90))

plotEmpiricalSuitRespMap(outfilename='empirical_mapP',
                         TempLim=c(12,30),
                         HumLim=c(60,90))

plotTheoreticalSuitRespMap(outfilename='theoretical_mapP')
```

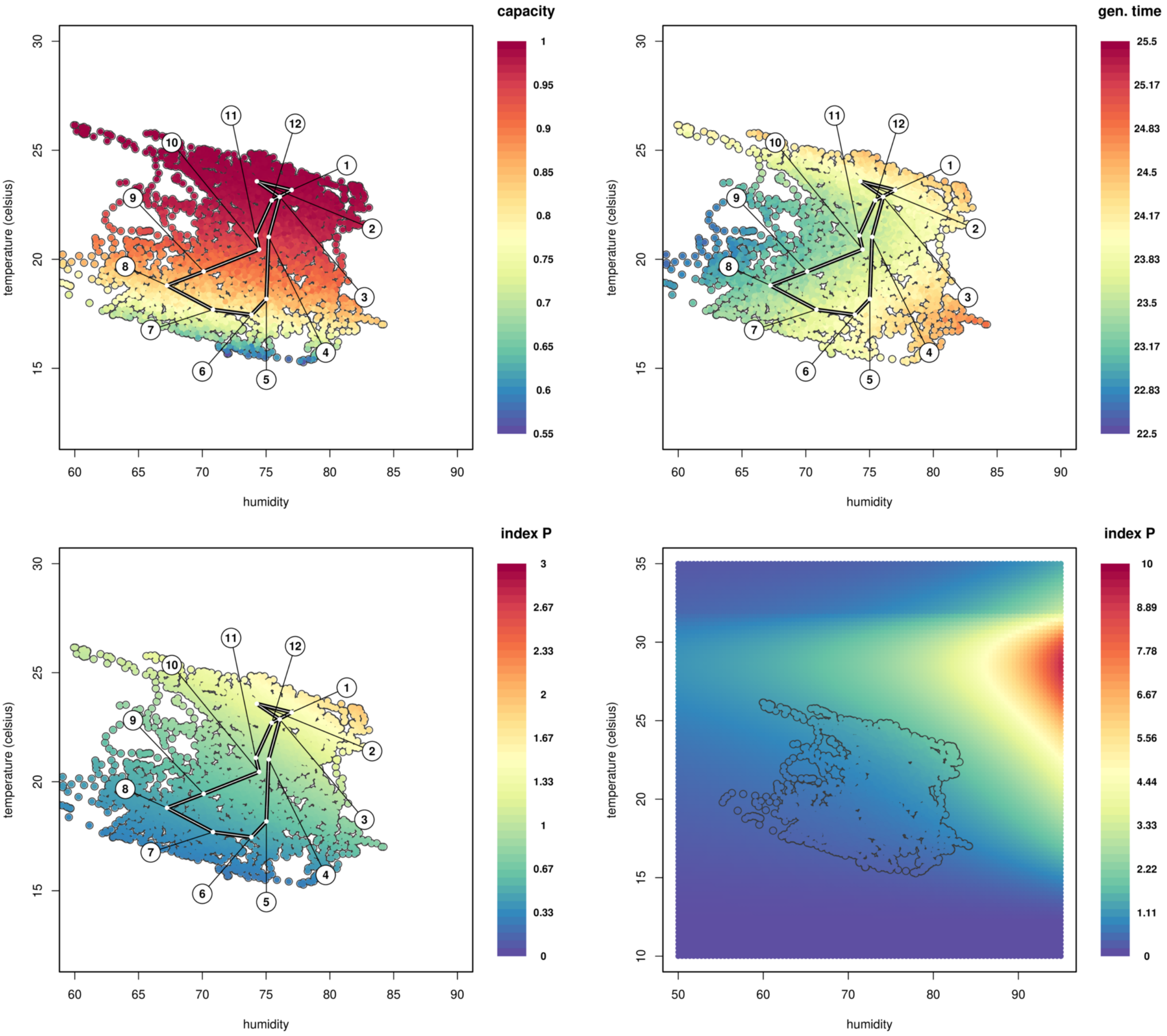

3.11 Data outputs

Functions for exportation of results into CSV files are also available. These include, for instance, exportation of mean and CI index P estimates over time. Below are some examples of these functions. For a list of all possible CSV outputs, please see MVSE's manual.

```
exportEcoCoefficients()
exportEntoParameters(Ns=1000)
exportEmpiricalIndexP()
exportGenerationTimes()
```

The output (tables) of *exportEntoParameters* and *exportEmpiricalIndexP* for the São Paulo results presented in the main text are included in Supplementary files.

3.12 Assumptions on missing climatic data

For all MVSE's functions, missing climatic data is allowed in the input (CSV) file by omitting entries (rows, i.e. time points, dates). The exception is the function *expectedPosterior*. Estimation of index P

(and any other variable) is performed only for time points given.

3.13 Minimal code example

The following minimal code executes the functionalities described in the above sections (for simplicity, it omits many of the options available per function - for the complete set of options, please see MVSE's manual included in the source code of the package).

```

## set import / export ########################

## set output folder and name-tag of this run

setOutputFilePathAndTag('testRun')

## read / store climatic time series

setEmpiricalClimateSeries('TableSz_climate_SaoPaulo.csv')

## set prior knowledge #######################

## set prior knowledge for mosquito parameters

setMosqLifeExpPrior(pmean=12, psd=2, pdist='gaussian')
setMosqIncPerPrior(pmean=7, psd=2, pdist='gaussian')
setMosqBitingPrior(pmean=0.25, psd=0.001, pdist='gaussian')

## set prior knowledge for human parameters

setHumanLifeExpPrior(pmean=71, psd=2, pdist='gaussian')

## set prior knowledge for human-virus parameters

setHumanIncPerPrior(pmean=5, psd=1, pdist='gaussian')
setHumanInfPerPrior(pmean=5, psd=1, pdist='gaussian')
setHumanMosqTransProbPrior(pmean=0.5, psd=0.01, pdist='gaussian')

## estimate variables / index p ##############

## estimate the ecological scaling factors

estimateEcoCoefficients(nMCMC=100000, bMCMC=0.5)

## simulate the empirical index P

simulateEmpiricalIndexP(nSample=1000)

## set a theoretical numeric range for climate

tR<- seq(10,35,length.out=100)
hR<- seq(0,100,length.out=100)
setTheoreticalClimateSeries(tR, hR)

## simulate the theoretical index P

simulateTheoreticalIndexP(nSample=1000)

## data outputs ##############################

## exports estimated posteriors of eco-coefficients
```

```

exportEcoCoefficients()

## exports entomological parameters in time

exportEntoParameters(Ns=1000)

## exports mean, CI, smoothing of index P in time

exportEmpiricalIndexP()

## graphical outputs ###########################

##plot posterior distributions of eco-coefficients

plotEcoCoefPosteriors()

##plot MCMC chains of eco-coefficients

plotEcoCoefMCMCChains()

##plot mosquito parameters in time and distributions

plotEntoParameters()

##plot (empirical) simulated index P in time

plotEmpiricalIndexP()

##plot empirical suitability map (colourmap)

plotEmpiricalSuitRespMap()

##plot theoretical suitability map (colourmap)

plotTheoreticalSuitRespMap()

## calculate and plot the seasons

suitableSeason()

## calculate and plot dates with peak index P

distributionPeak(nSamples=10)

## get a smooth estimator of index P

expectedPosterior(nSamples=10)
```

References

[1] Lourenço J, Recker M, Lourenco J, Recker M, Lourenço J, Recker M. The 2012 Madeira dengue outbreak: epidemiological determinants and future epidemic potential. PLoS neglected tropical diseases. 2014;8(8):e3083. doi:10.1371/journal.pntd.0003083.

[2] Faria NR, da Costa AC, Lourenço J, Loureiro P, Lopes ME, Ribeiro R, et al. Genomic and epidemiological characterisation of a dengue virus outbreak among blood donors in Brazil. Scientific Reports. 2017;7(1):15216. doi:10.1038/s41598-017-15152-8.

[3] Lourenço J, de Lima MM, Faria NR, Walker A, Kraemer MUGU, Villabona-Arenas CJ, et al. Epidemiological and ecological determinants of Zika virus transmission in an urban setting. eLife. 2017;6. doi:10.7554/eLife.29820.

[4] Yang HM, Macoris MLG, Galvani KC, Andrighetti MTM, Wanderley DMV. Assessing the effects of temperature on the population of Aedes aegypti, the vector of dengue. Epidemiology and infection. 2009;137(8):1188–202. doi:10.1017/S0950268809002040.

[5] Focks Da, Daniels E, Haile DG, Keesling JE. A simulation model of the epidemiology of urban dengue fever: literature analysis, model development, preliminary validation, and samples of simulation results. The American journal of tropical medicine and hygiene. 1995;53(5):489–506.

[6] Schoolfield RM, Sharpe PJHH, Magnuson CE. Non-linear regression of biological temperature-dependent rate models based on absolute reaction-rate theory. Journal of theoretical biology. 1981;88(4):719–31. doi:10.1016/0022-5193(81)90246-0.

[7] Otero M, Solari HG, Schweigmann N. A stochastic population dynamics model for Aedes aegypti: formulation and application to a city with temperate climate. Bulletin of mathematical biology. 2006;68(8):1945–1974. doi:10.1007/s11538-006-9067-y.

[8] Lambrechts L, Paaijmans KP, Fansiri T, Carrington LB, Kramer LD, Thomas MB, et al. Impact of daily temperature fl uctuations on dengue virus transmission by Aedes aegypti. Proceedings of the National Academy of Sciences of the United States of America. 2011;108(18):7460–5. doi:10.1073/pnas.1101377108.

[9] Bicout DJ, Sabatier P. Mapping Rift Valley Fever vectors and prevalence using rainfall variations. Vector borne and zoonotic diseases (Larchmont, NY). 2004;4(1):33–42. doi:10.1089/153036604773082979.

[10] Tran A, L'Ambert G, Lacour G, Benoît R, Demarchi M, Cros M, et al. A rainfall- and temperature-driven abundance model for Aedes albopictus populations. International Journal of Environmental Research and Public Health. 2013;10(5):1698–1719. doi:10.3390/ijerph10051698.

[11] Yasuno M, Tonn RJ. A study of biting habits of Aedes aegypti in Bangkok, Thailand. Population (English Edition). 1960;43(2):319–325.

[12] Alto BW, Juliano SA. Precipitation and temperature effects on populations of Aedes albopictus (Diptera: Culicidae): Implications for range expansion. Journal of Medical Entomology. 2001;38(5):646–656. doi:10.1603/0022-2585-38.5.646.

[13] Brady OJ, Johansson MA, Guerra CA, Bhatt S, Golding N, Pigott DM, et al. Modelling adult Aedes aegypti and Aedes albopictus survival at different temperatures in laboratory and field settings. Parasites & vectors. 2013;6(1):351. doi:10.1186/1756-3305-6-351.

[14] Hugo LE, Jeffery JAL, Trewin BJ, Wockner LF, Thi Yen N, Le NH, et al. Adult Survivorship of the Dengue Mosquito Aedes aegypti Varies Seasonally in Central Vietnam. PLoS Neglected Tropical Diseases. 2014;8(2):e2669. doi:10.1371/journal.pntd.0002669.

[15] Trpis M, Hausermann W. Population Size, Dispersal, and Longevity of Domestic Aedes aegypti aegypti (Diptera: Culicidae) by MarkReleaseRecapture in the Village of Shauri Moyo in Eastern. Journal of medical. 1995;32(1):27—-33(7).

[16] Ferguson NM, Cucunubá ZM, Dorigatti I, Nedjati-Gilani GL, Donnelly CA, Basáñez MGG, et al. Countering the Zika epidemic in Latin America. Science. 2016;353(6297):353–354. doi:10.1126/science.aag0219.

[17] Li MI, Wong PSJ, Ng LC, Tan CH. Oral Susceptibility of Singapore Aedes (Stegomyia) aegypti (Linnaeus) to Zika Virus. PLoS Neglected Tropical Diseases. 2012;6(8). doi:10.1371/journal.pntd.0001792.

[18] Wong PSJ, Li MzI, Chong CS, Ng LC, Tan CH. Aedes (Stegomyia) albopictus (Skuse): A Potential Vector of Zika Virus in Singapore. PLoS Neglected Tropical Diseases. 2013;7(8):1–5. doi:10.1371/journal.pntd.0002348.

[19] Trpis M, Hausermann W. Dispersal and other population parameters of Aedes aegypti in an African village and their possible significance in epidemiology of vector-borne diseases. American Journal of Tropical Medicine and Hygiene. 1986;35(6):1263–1279.

[20] Lessler J, Ott CT, Carcelen AC, Konikoff JM, Williamson J, Bi Q, et al. Times to key events in Zika virus infection and implications for blood donation: A systematic review. Bulletin of the World Health Organization. 2016;94(11):841–849. doi:10.2471/BLT.16.174540.

[21] Fick SE, Hijmans RJ. WorldClim 2: new 1-km spatial resolution climate surfaces for global land areas. International Journal of Climatology. 2017;37(12):4302–4315. doi:10.1002/joc.5086.

[22] Faria NR, Azevedo RdSdS, Kraemer MUG, Souza R, Cunha MS, Hill SC, et al. Zika virus in the Americas: Early epidemiological and genetic findings. Science (New York, NY). 2016;352(6283):aaf5036. doi:10.1126/science.aaf5036.

[23] Faria NR, Quick J, Claro IM, Thézé J, de Jesus JG, Giovanetti M, et al. Establishment and cryptic transmission of Zika virus in Brazil and the Americas. Nature. 2017;546(7658):406–410. doi:10.1038/nature22401.

[24] Lee JS, Lourenço J, Gupta S, Farlow A. A multi-country study of dengue vaccination strategies with Dengvaxia and a future vaccine candidate in three dengue-endemic countries: Vietnam, Thailand, and Colombia. Vaccine. 2018;36(17):2346–2355. doi:10.1016/j.vaccine.2018.03.002.

[25] Flasche S, Jit M, Rodríguez-Barraquer I, Coudeville L, Recker M, Koelle K, et al. The Long-Term Safety, Public Health Impact, and Cost-Effectiveness of Routine Vaccination with a Recombinant, Live-Attenuated Dengue Vaccine (Dengvaxia): A Model Comparison Study. PLoS Medicine. 2016;13(11):1–19. doi:10.1371/journal.pmed.1002181.

[26] Villabona-Arenas CJ, Miranda-Esquivel DR, Jimenez REO. Phylogeny of dengue virus type 3 circulating in Colombia between 2001 and 2007. Tropical Medicine & International Health. 2009;14(10):1241–1250. doi:10.1111/j.1365-3156.2009.02339.x.

[27] Murray NEA, Quam MB, Wilder-Smith A. Epidemiology of dengue: Past, present and future prospects. Clinical Epidemiology. 2013;5:299–309. doi:10.2147/CLEP.S34440.

[28] San Mart\'\in JL, Brathwaite O, Zambrano B, Solórzano JO, Bouckenooghe A, Dayan GH, et al. The epidemiology of dengue in the americas over the last three decades: a worrisome reality. The American journal of tropical medicine and hygiene. 2010;82(1):128–135. doi:10.4269/ajtmh.2010.09-0346.

[29] Pinault LL, Hunter FF. New highland distribution records of multiple Anopheles species in the Ecuadorian Andes. Malaria Journal. 2011;10(August). doi:10.1186/1475-2875-10-236.

[30] James G, Witten D, Hastie T, Tibshirani R. An Introduction to Statistical Learning. vol. 103 of Springer Texts in Statistics. New York, NY: Springer New York; 2013.

[31] Tibshirani RJ, Taylor J. The solution path of the generalized lasso. The Annals of Statistics. 2011;39(3):1335–1371. doi:10.1214/11-AOS878.
