# Supplementary figures and images for: MVSE: An R‐package that estimates a climate‐driven mosquito‐borne viral suitability index
Source: Methods Ecol Evol. 2019 Jun 19;10(8):1357–70. doi: 10.1111/2041-210X.13205 (PMC7202302; doi:10.1111/2041-210X.13205)

JANUARY

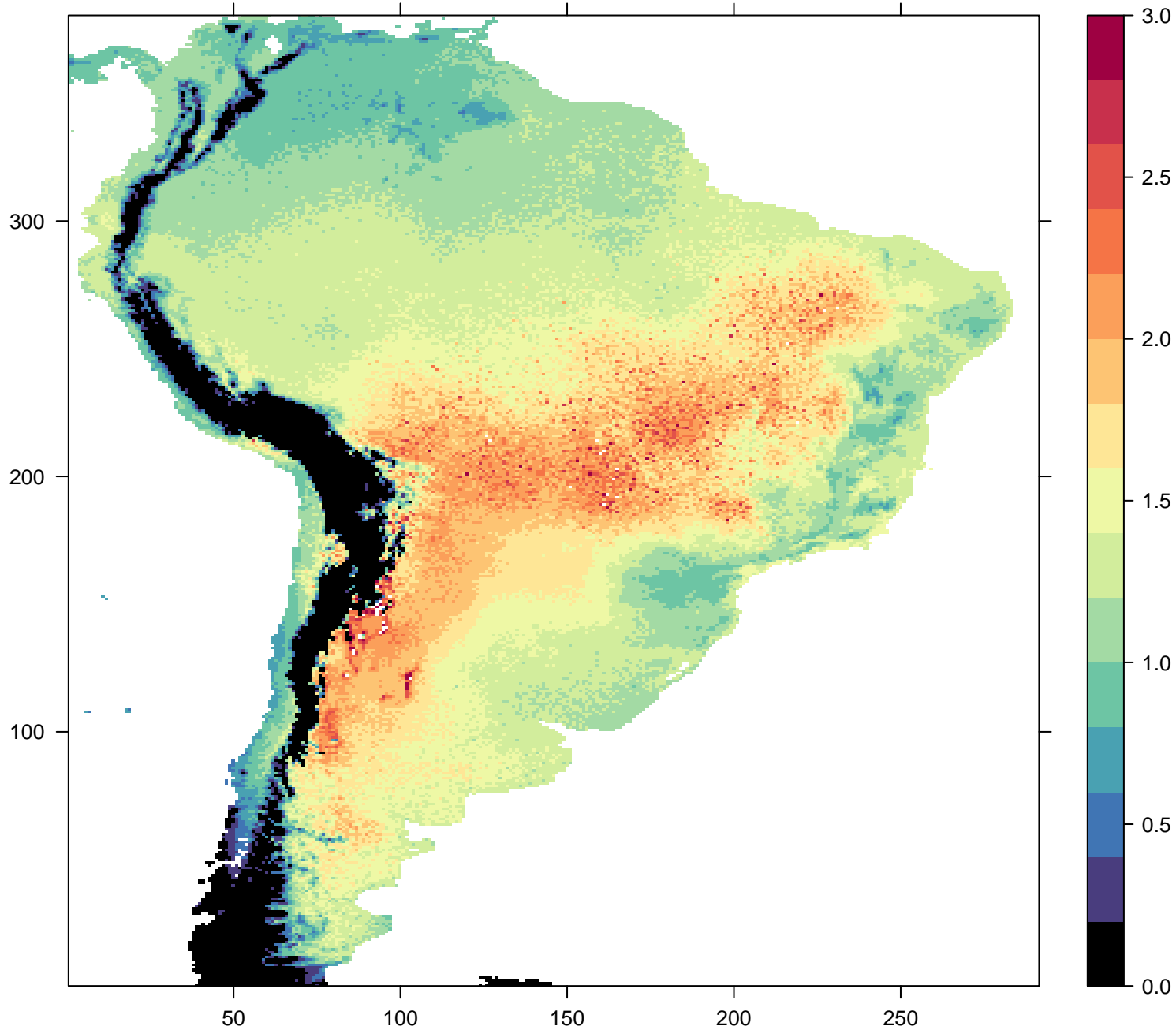

# FEBRUARY

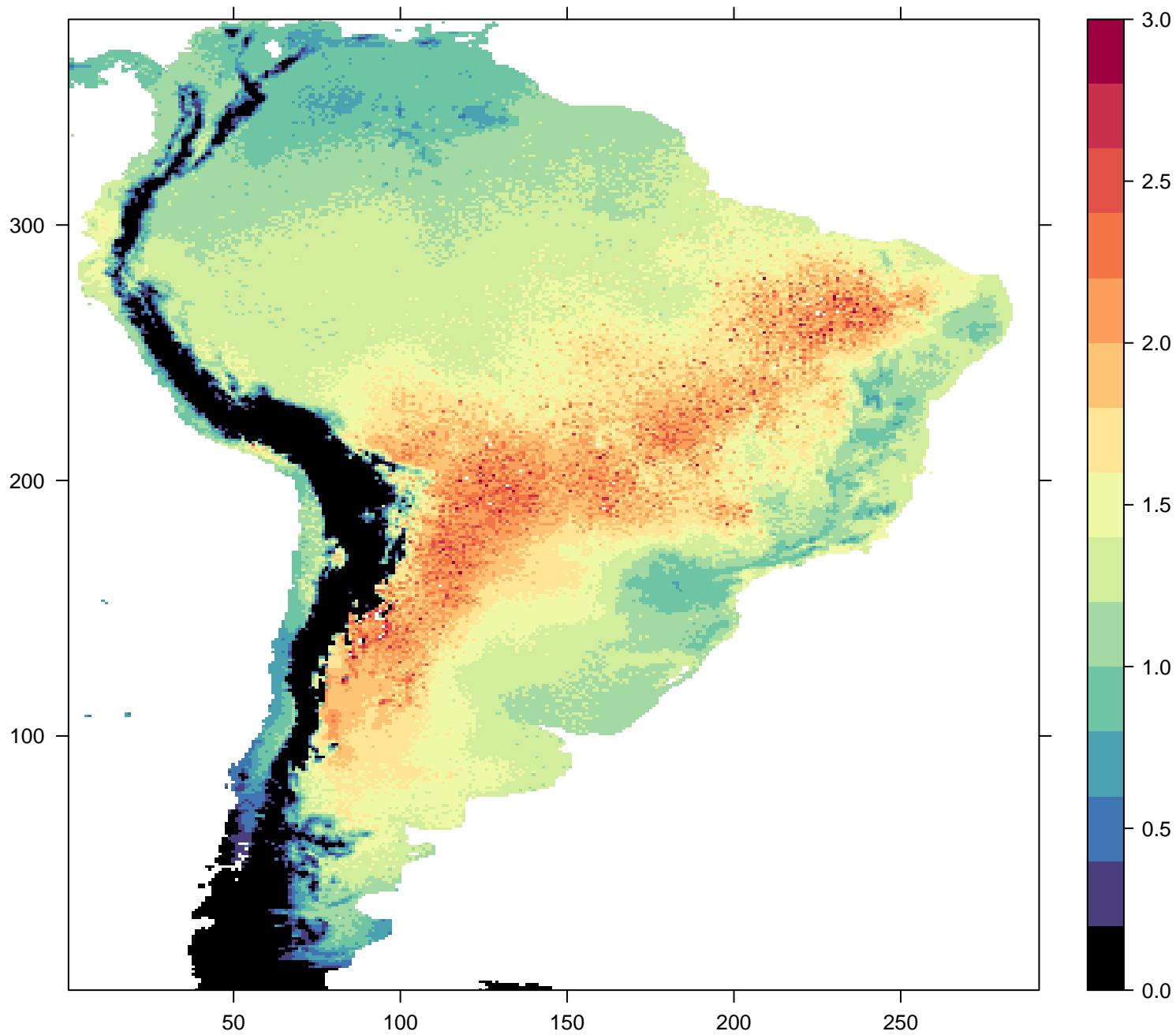

MARCH

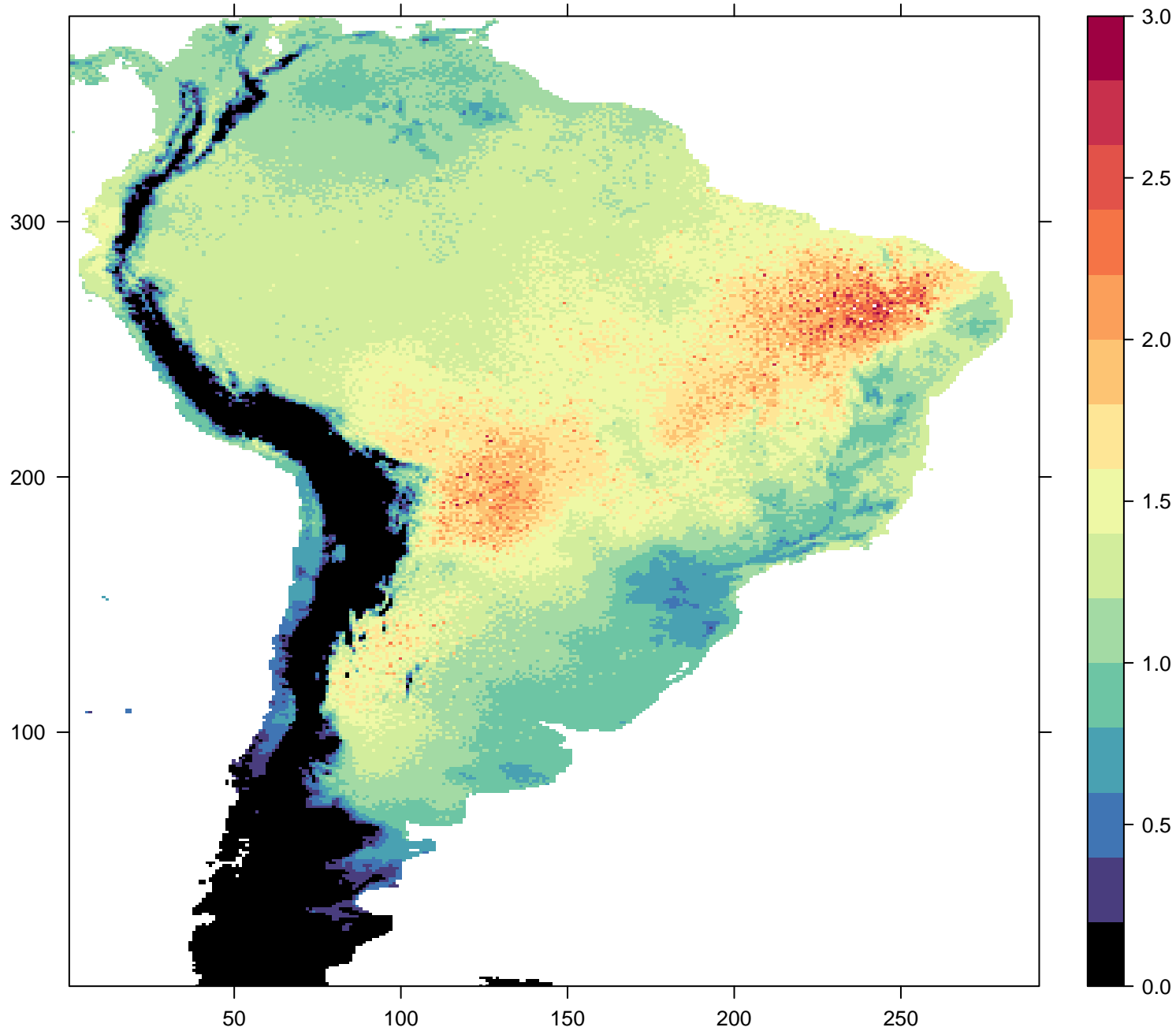

APRIL

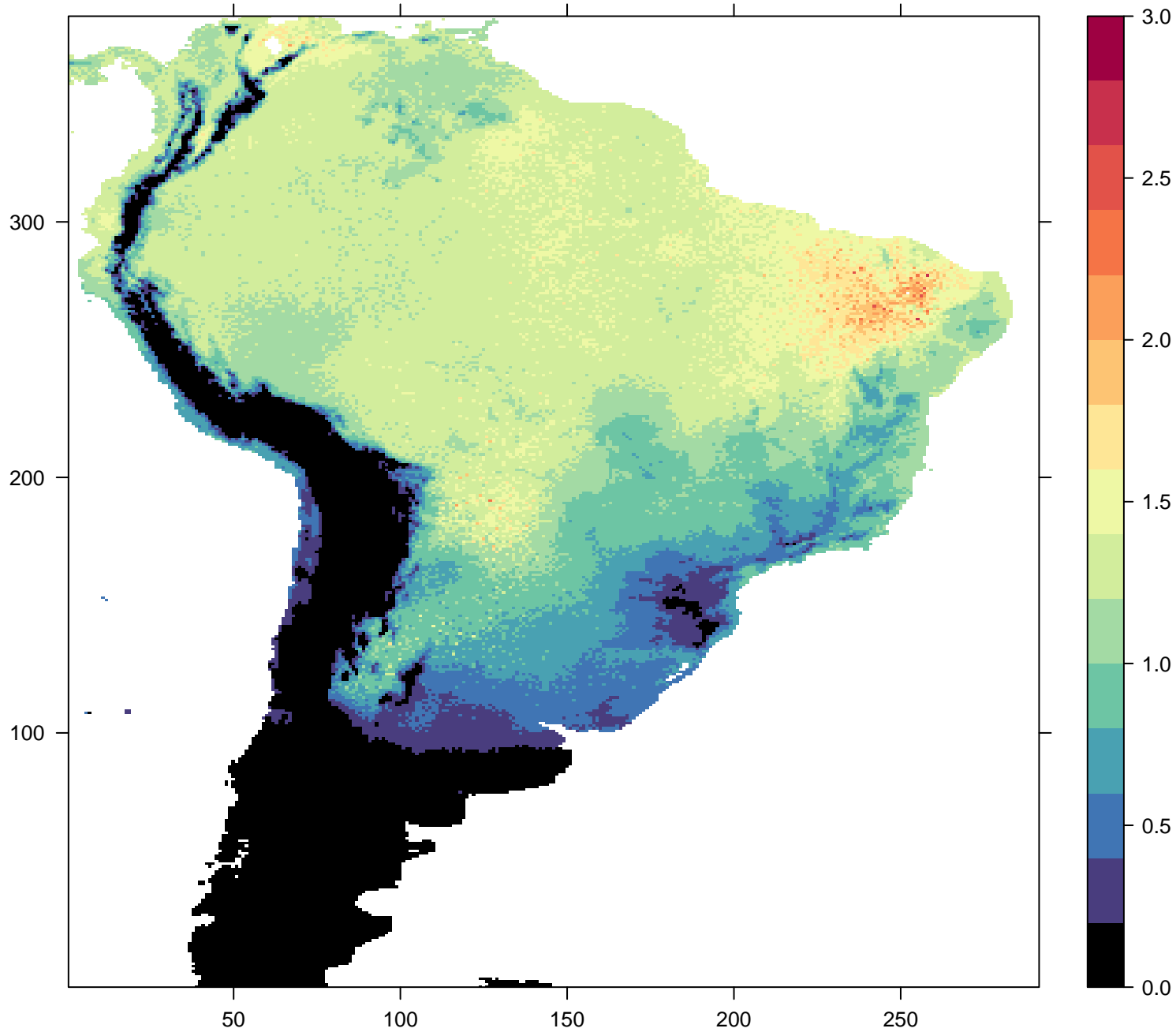

MAY

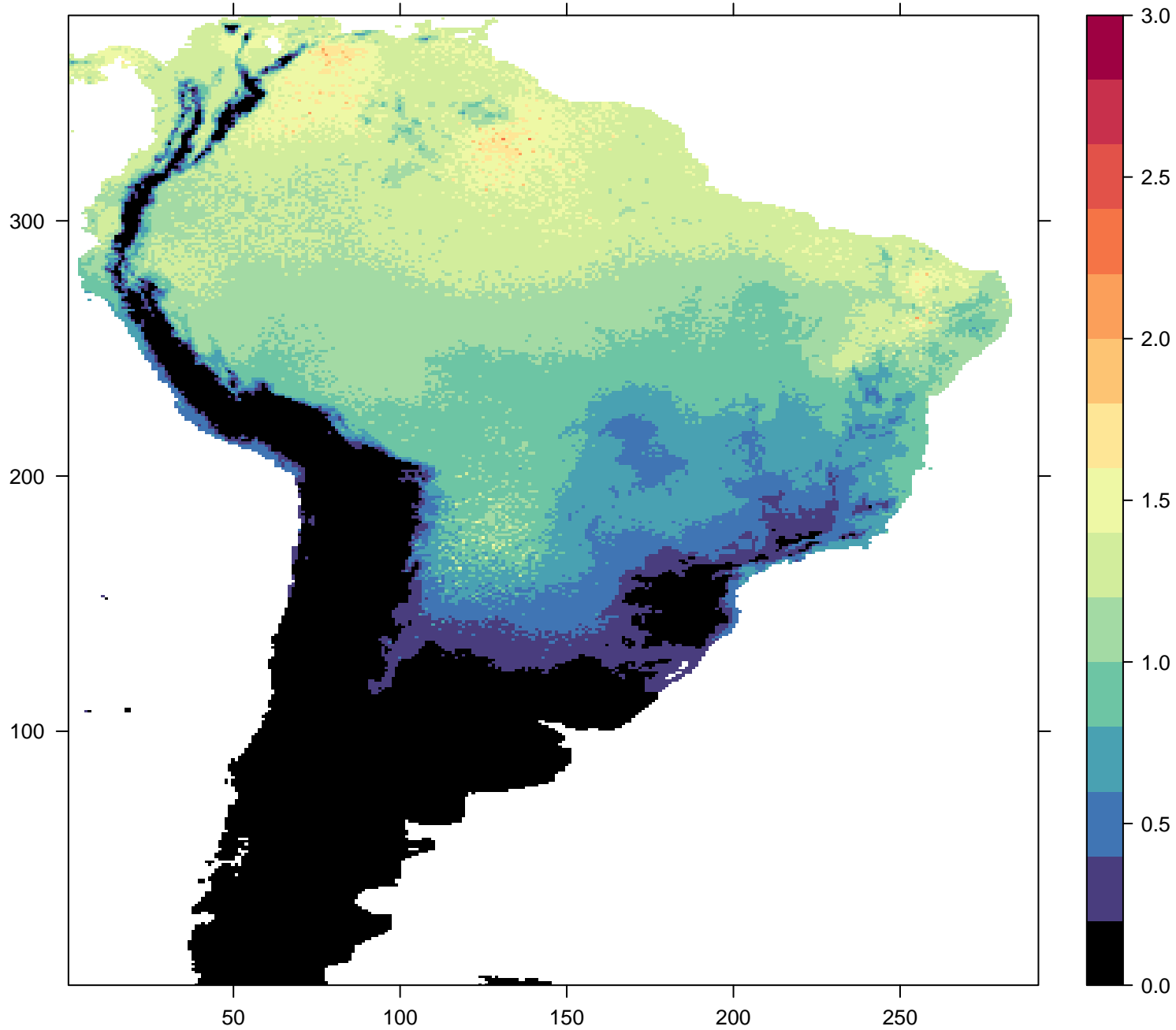

JUNE

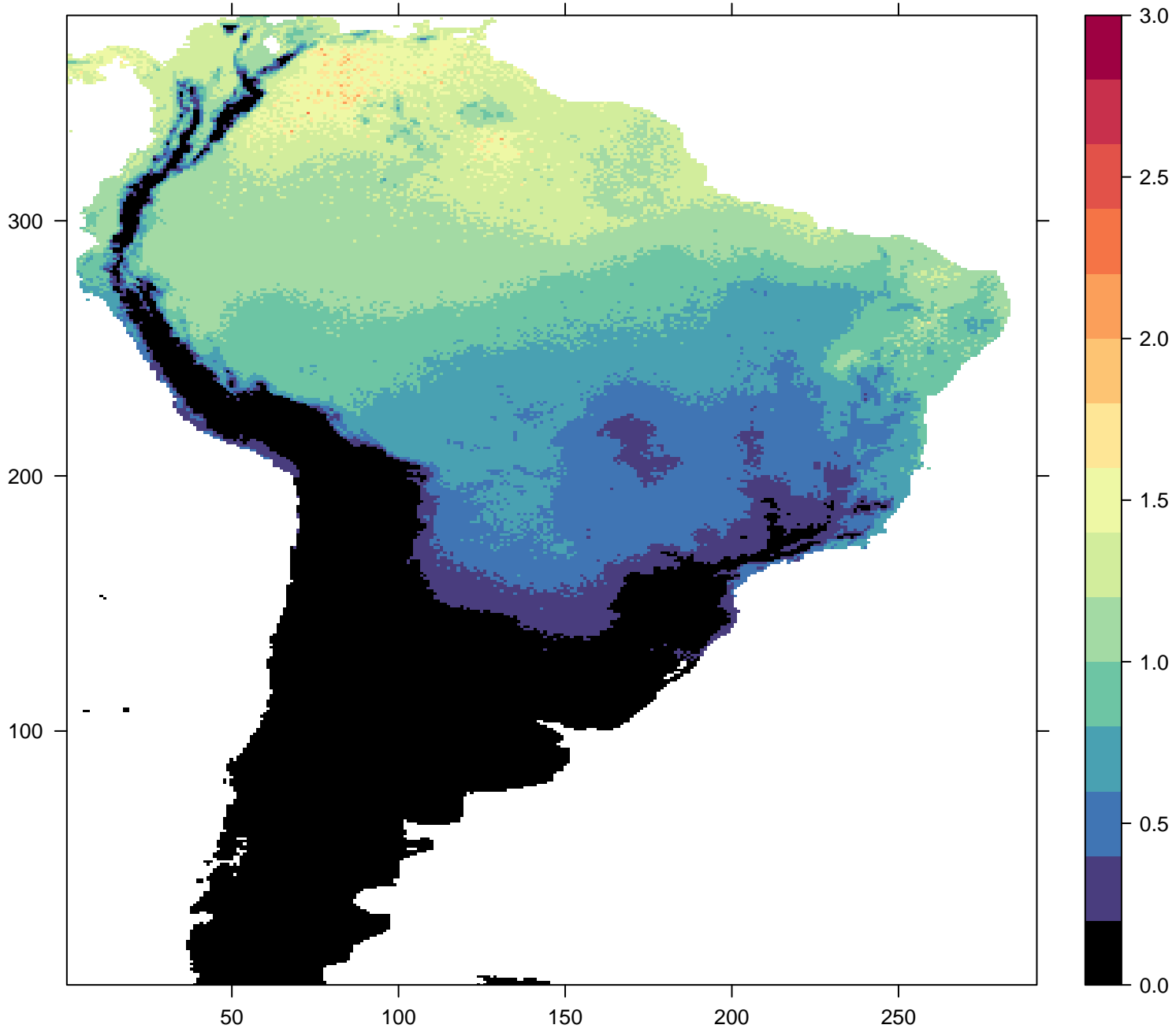

JULY

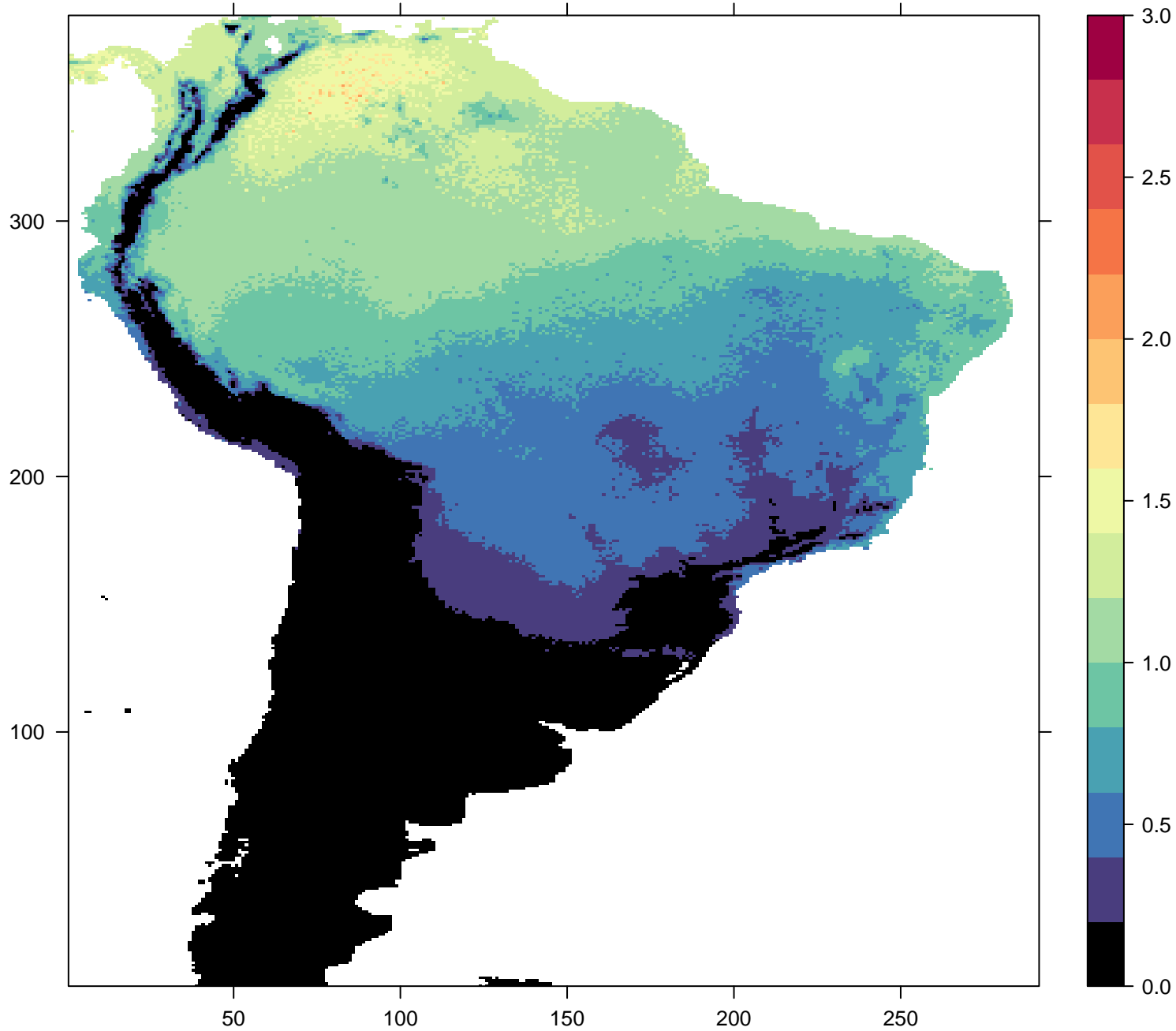

AUGUST

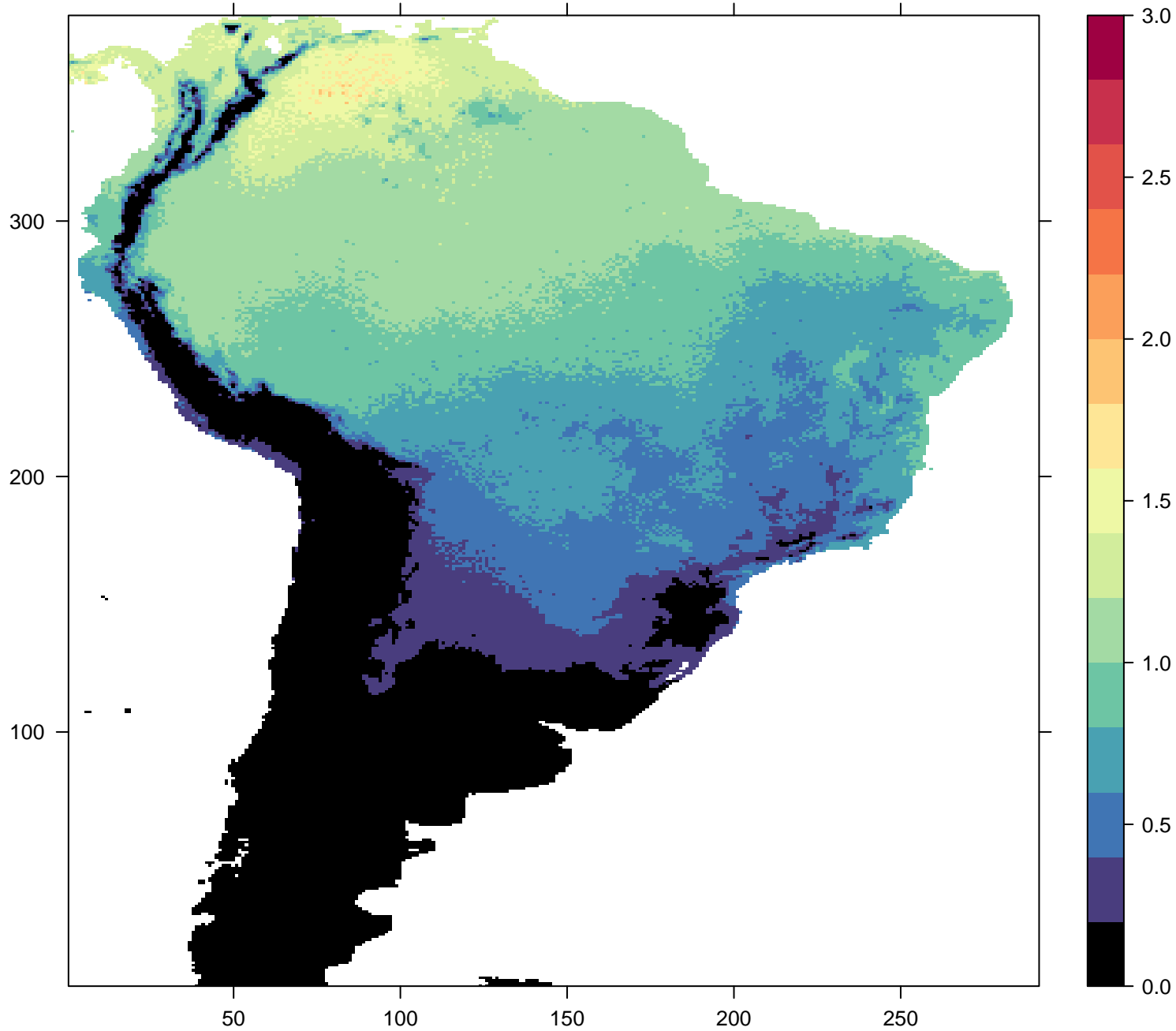

SEPTEMBER

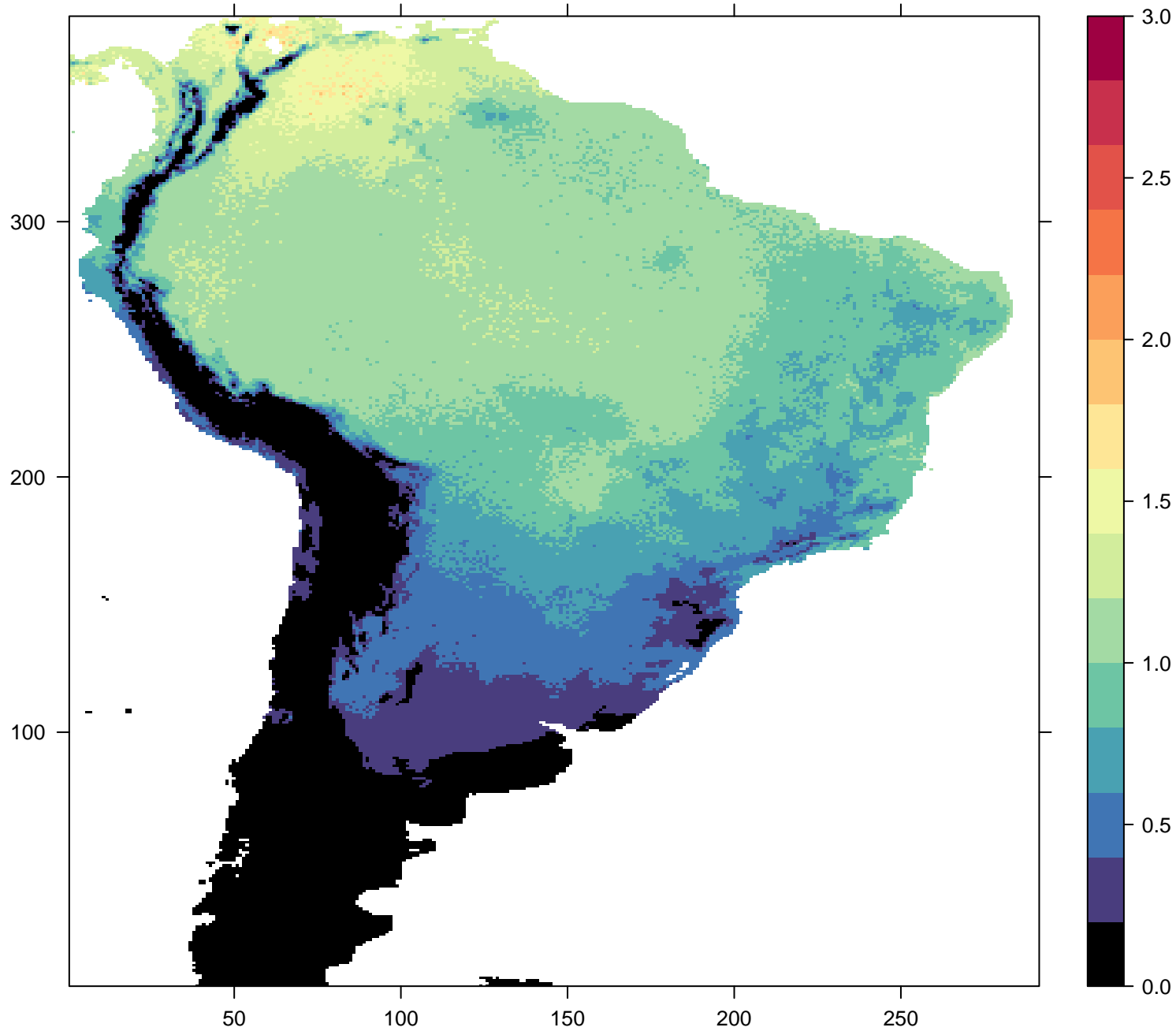

OCTOBER

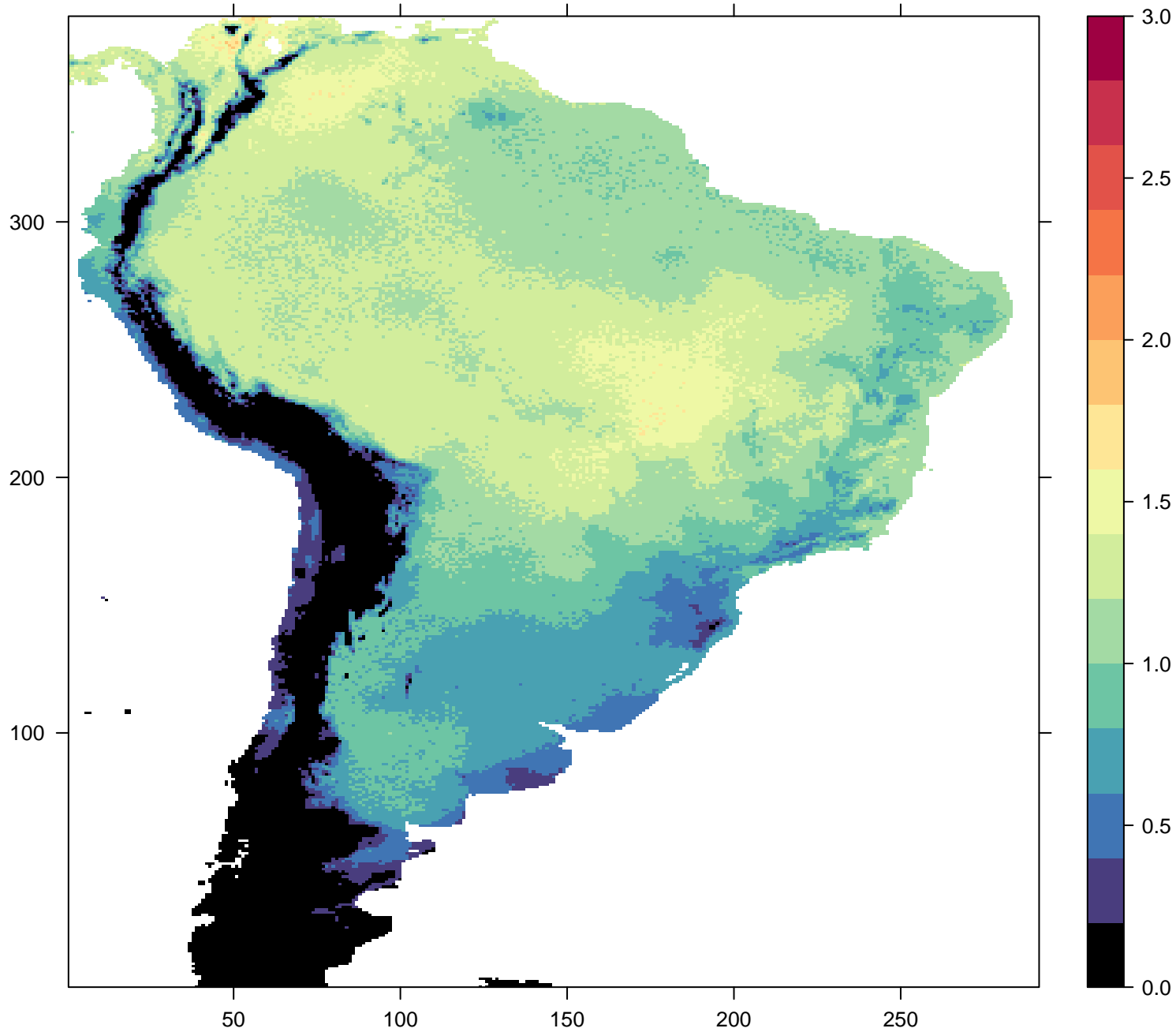

NOVEMBER

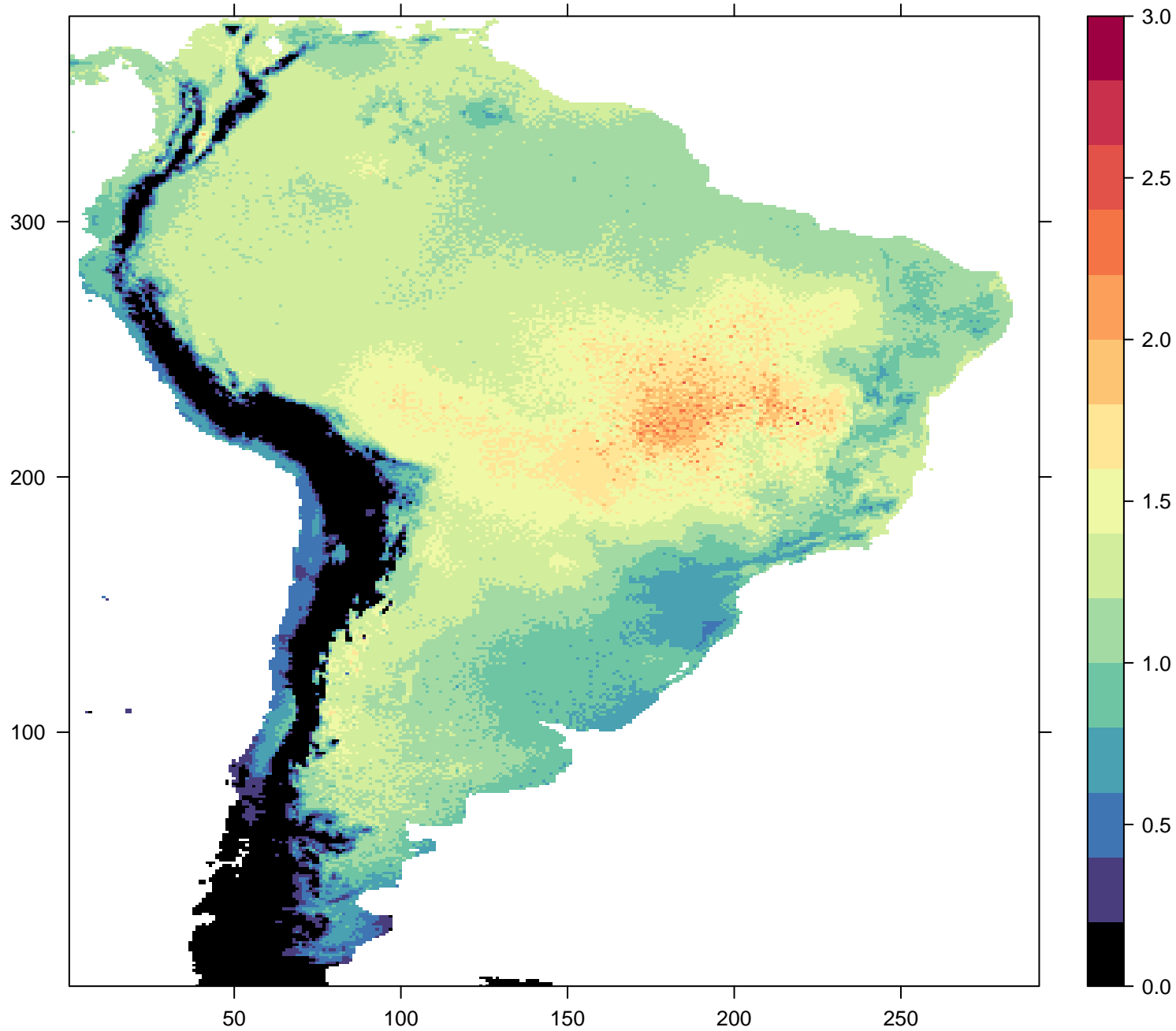

DECEMBER

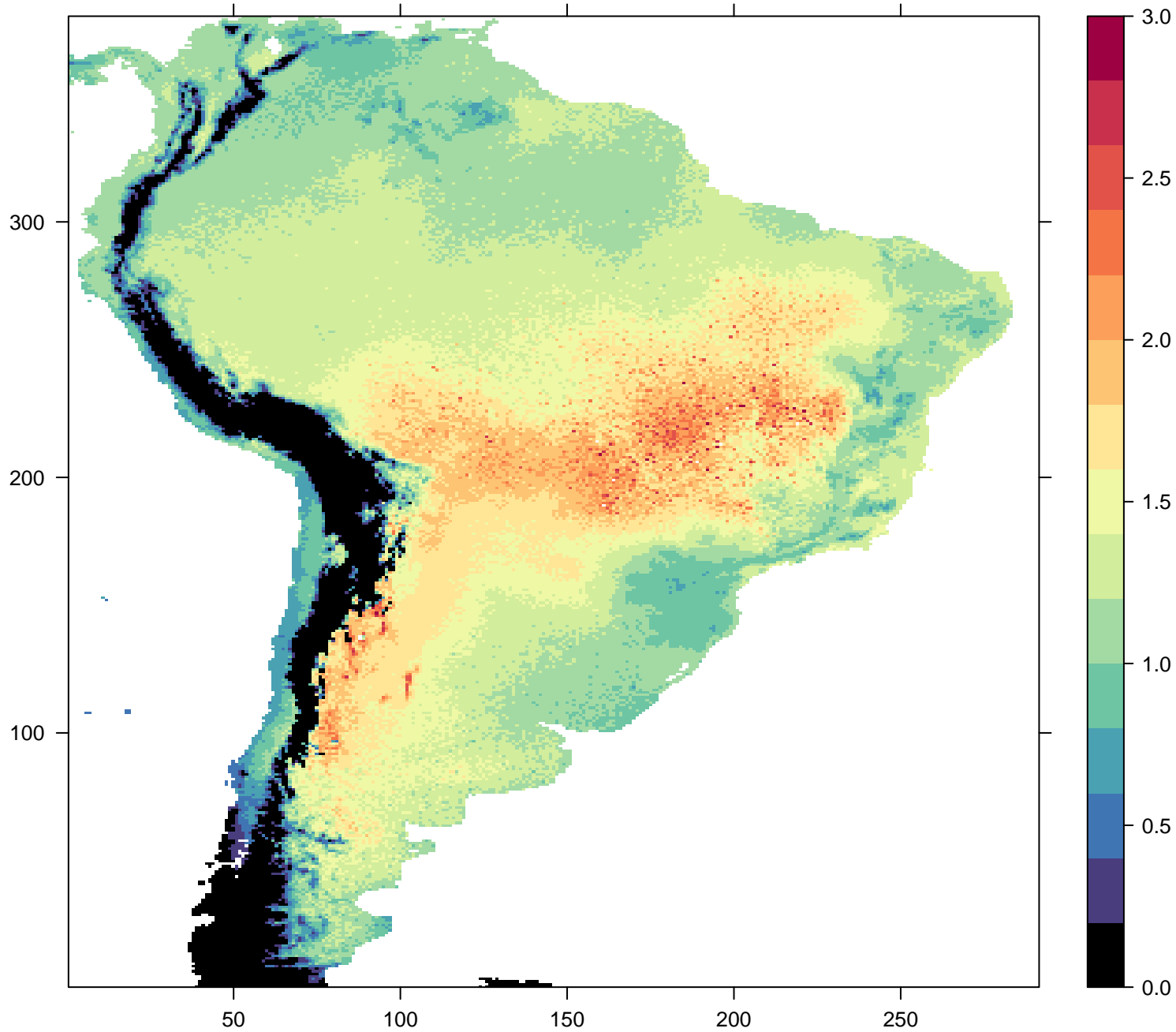

Supplement: Supplementary file 1 [file MEE3-10-1357-s001.zip › mee313205-sup-0001-SpatioTemporalFramesS1.pdf]

# JANUARY

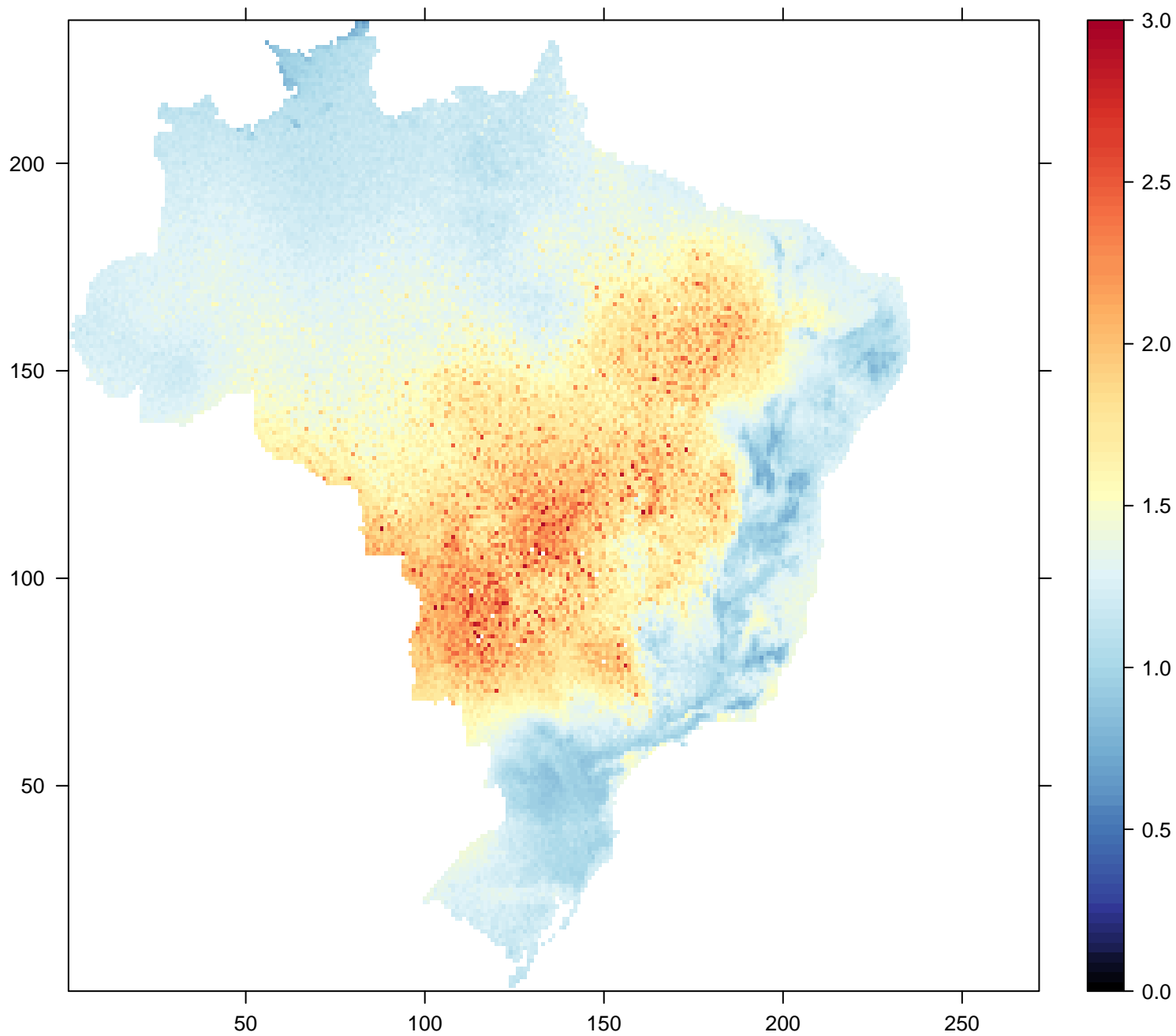

# FEBRUARY

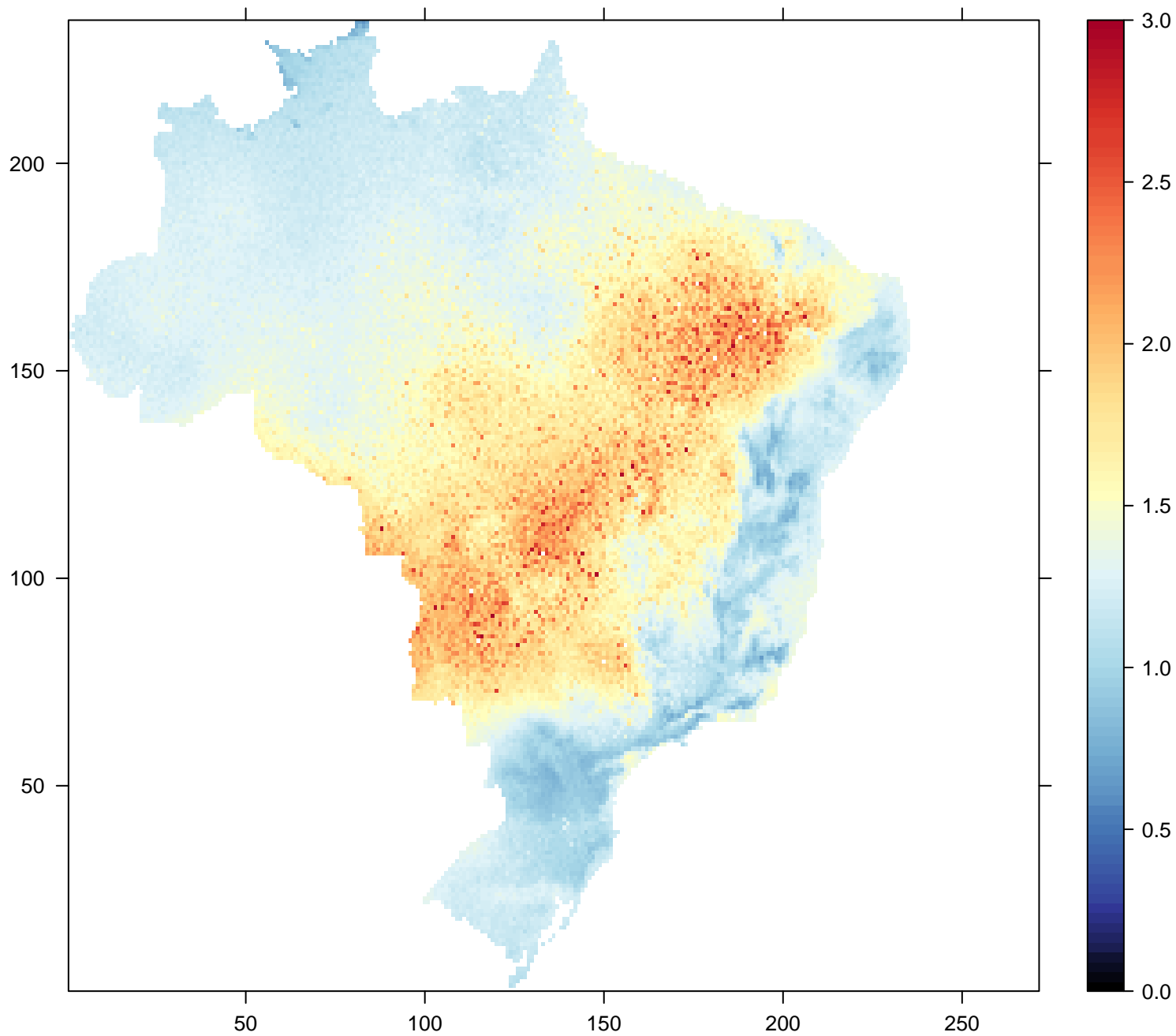

**MARCH**

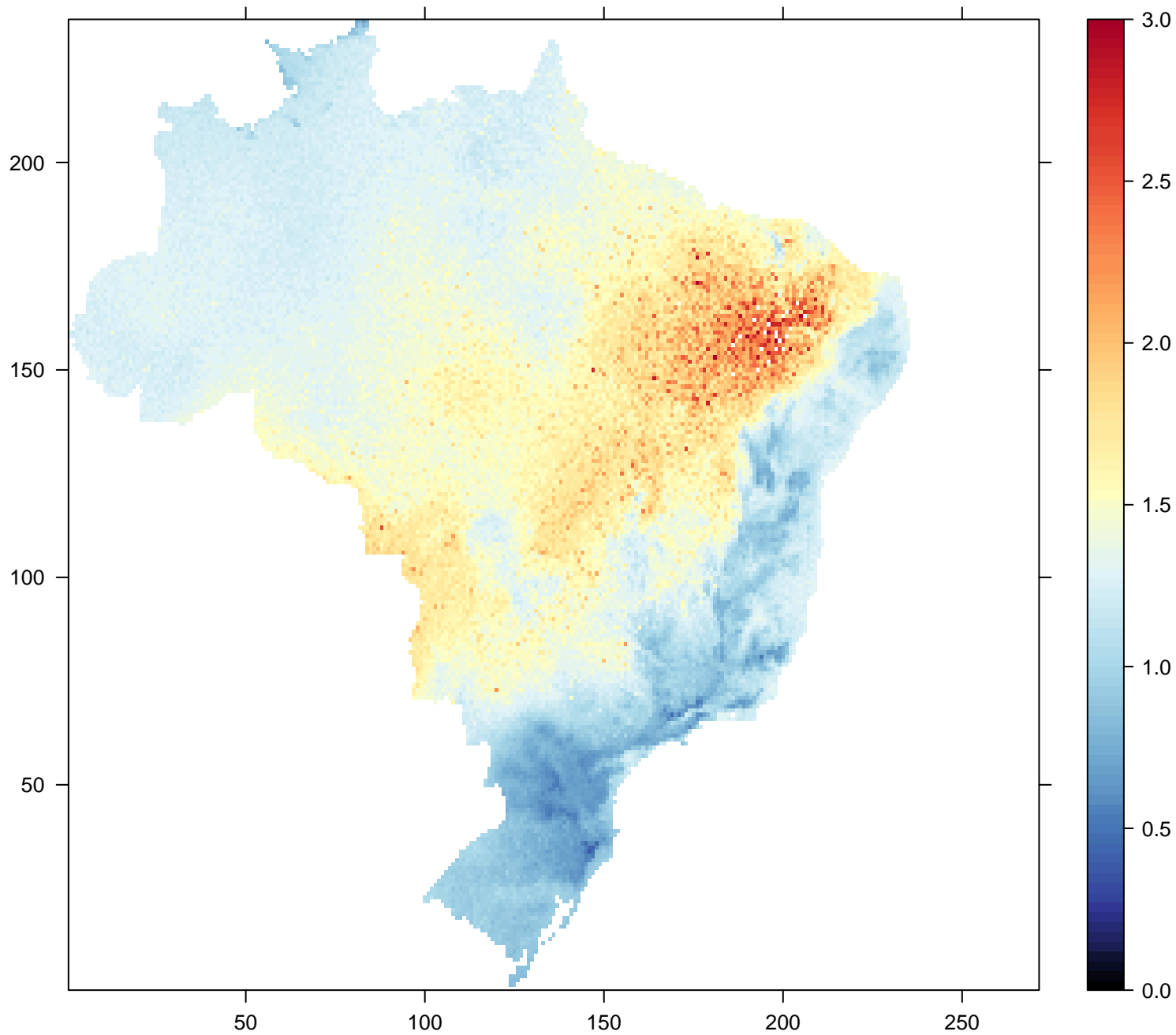

APRIL

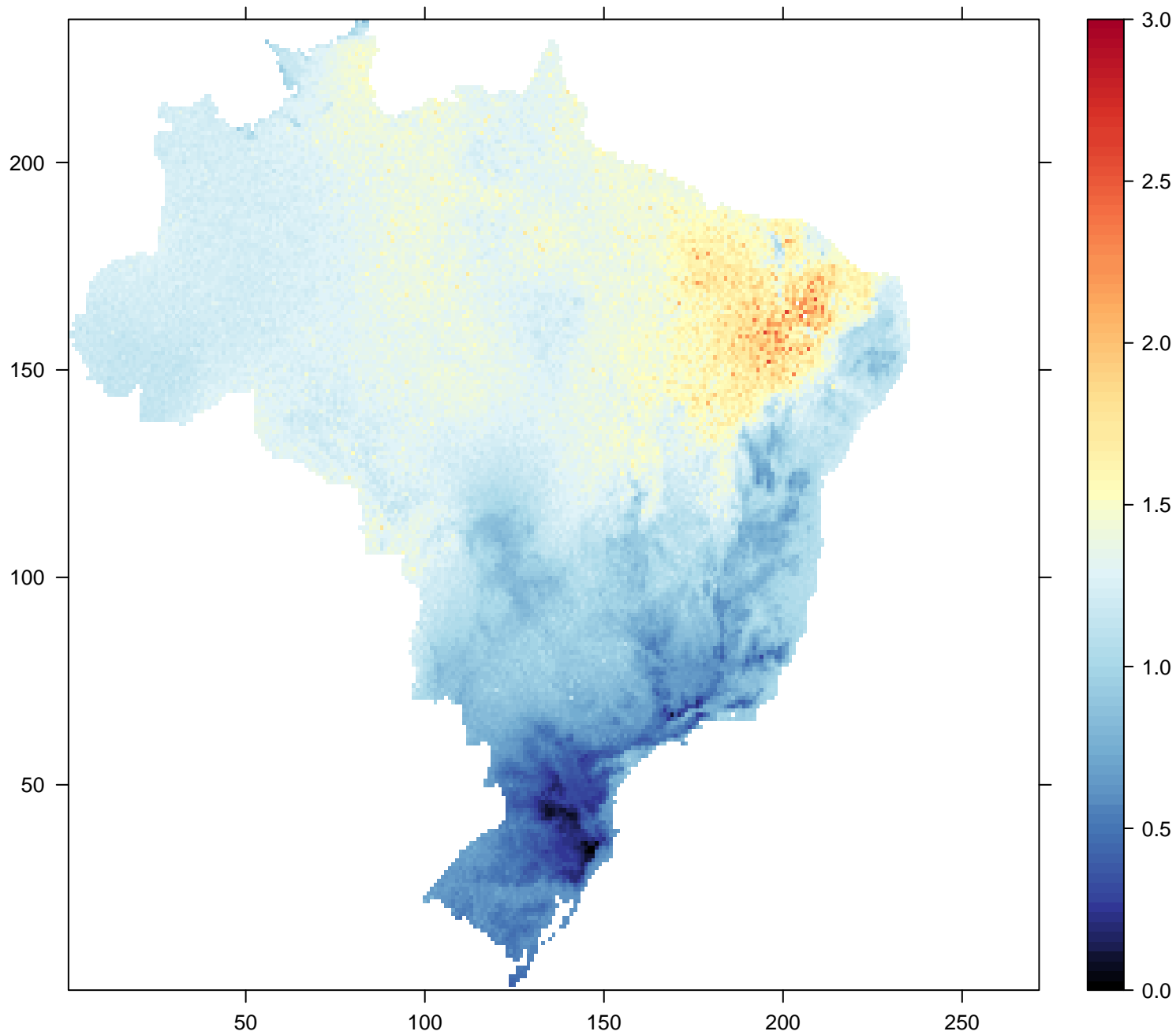

MAY

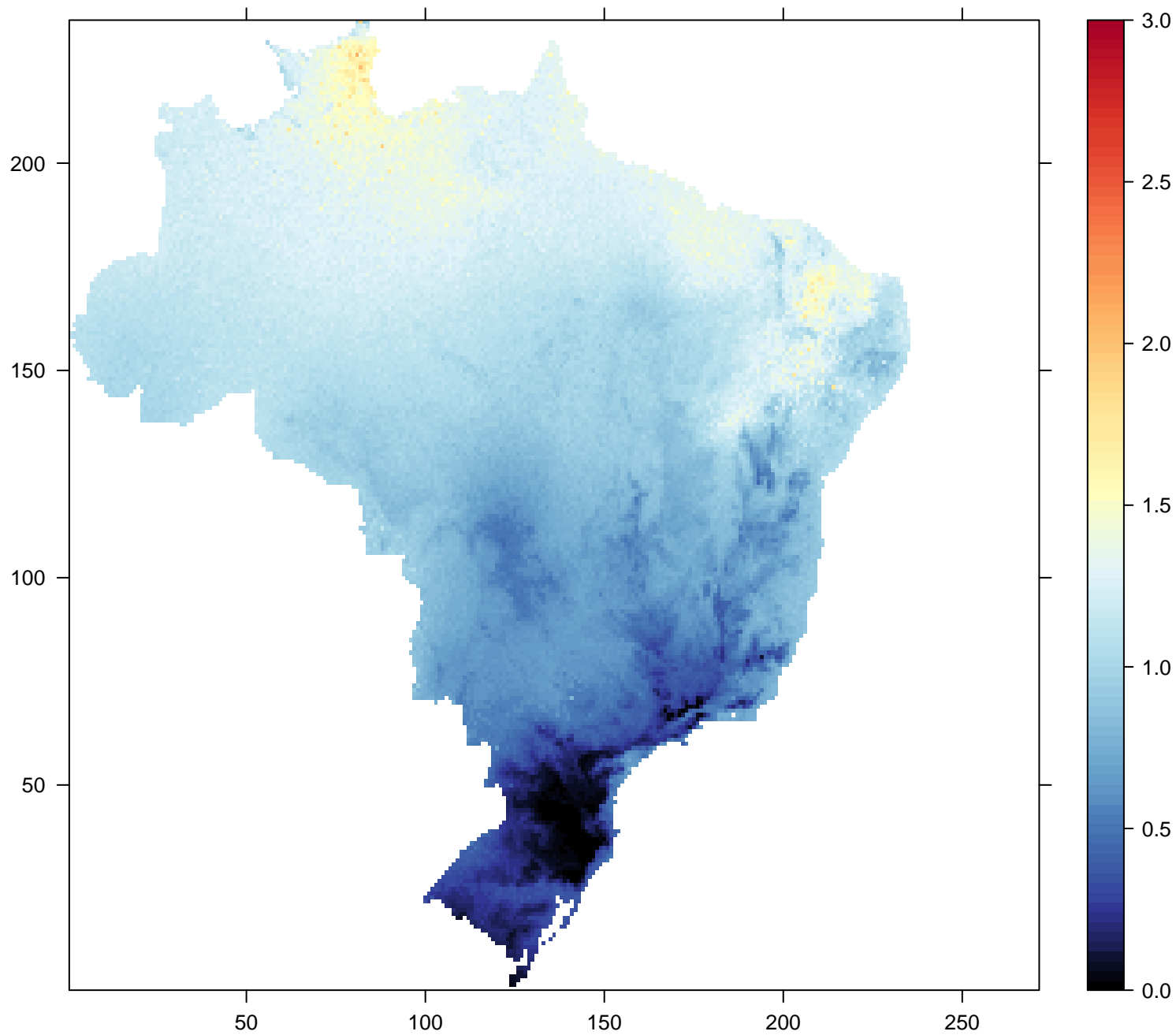

JUNE

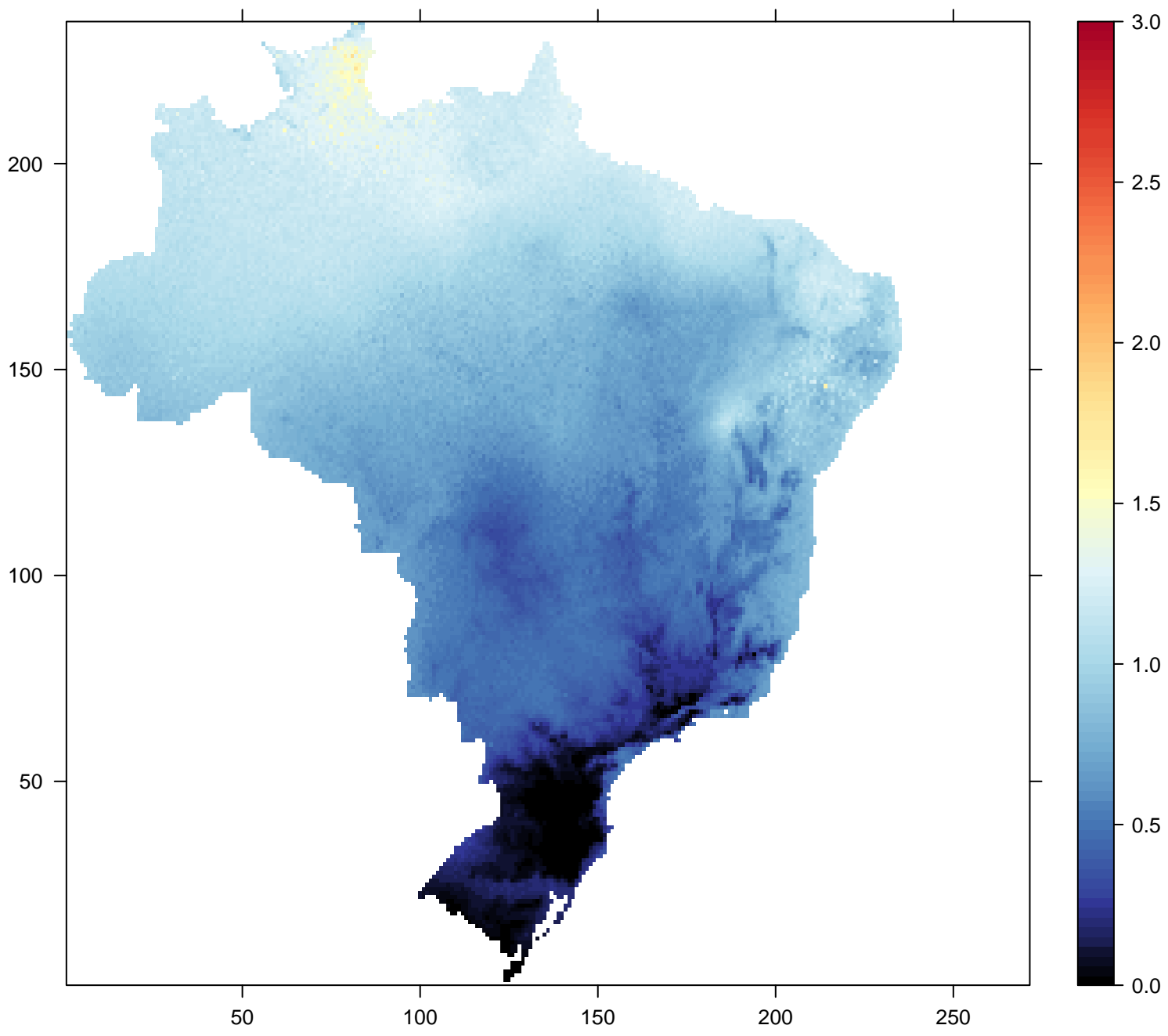

JULY

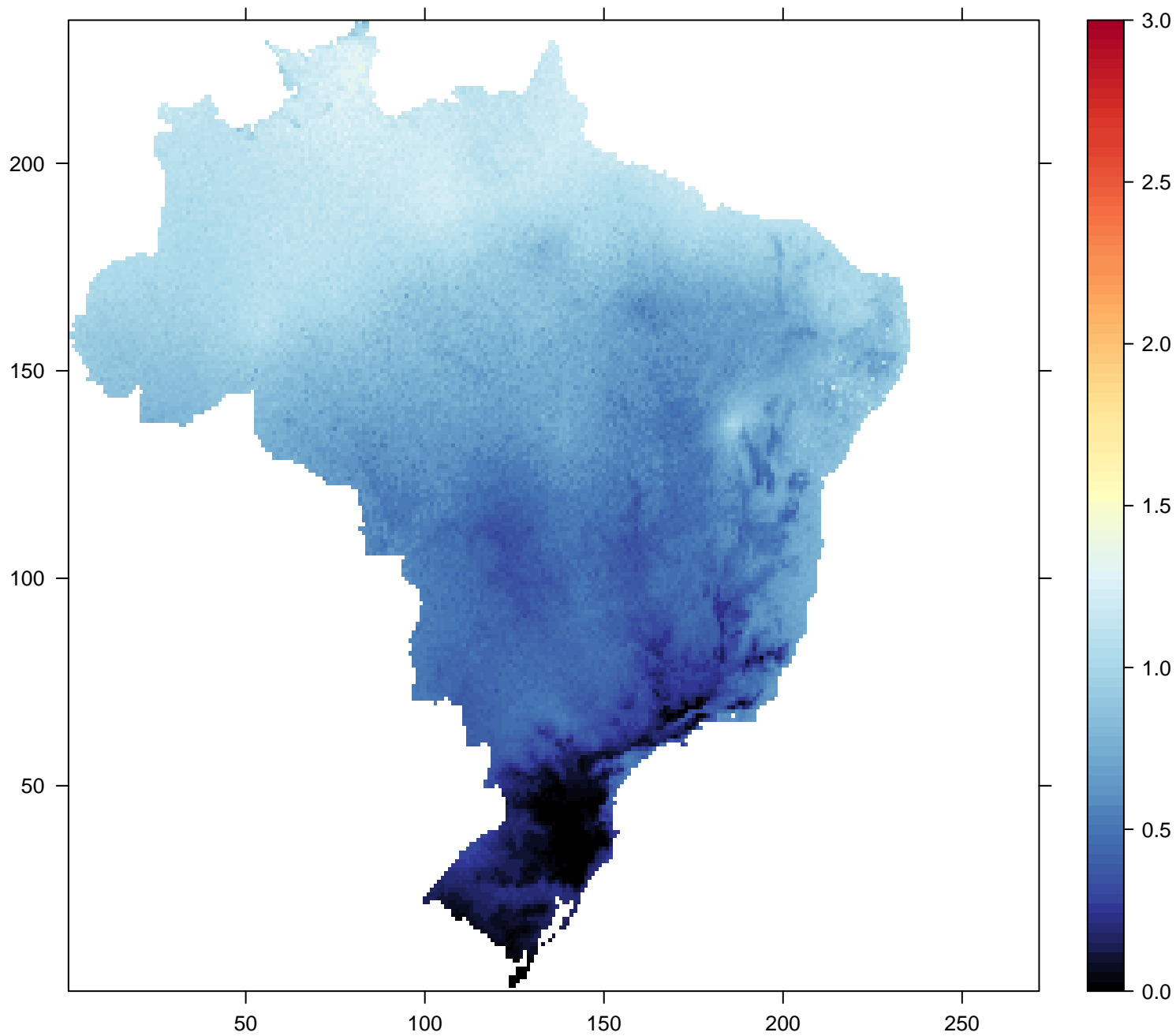

**AUGUST**

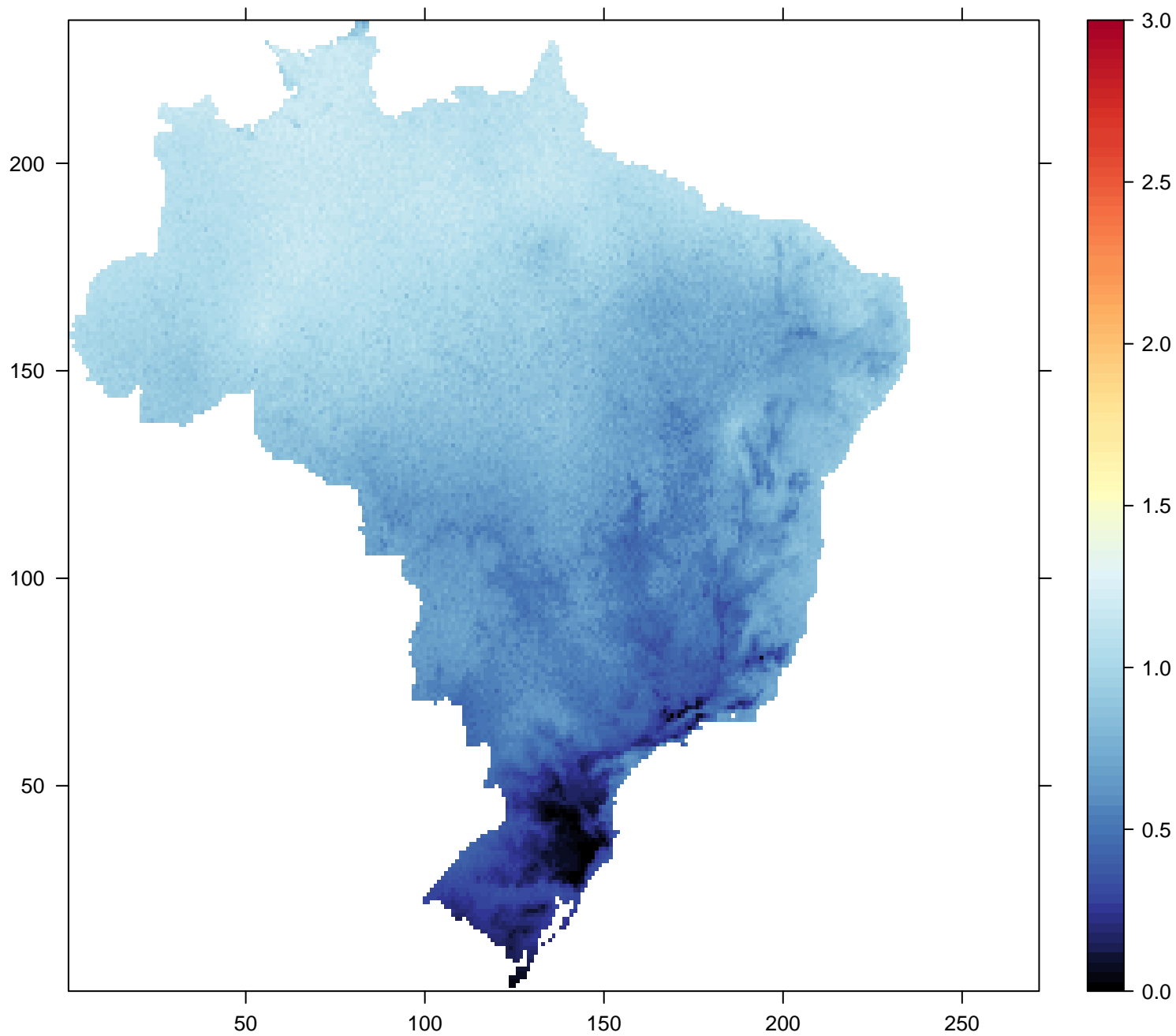

# SEPTEMBER

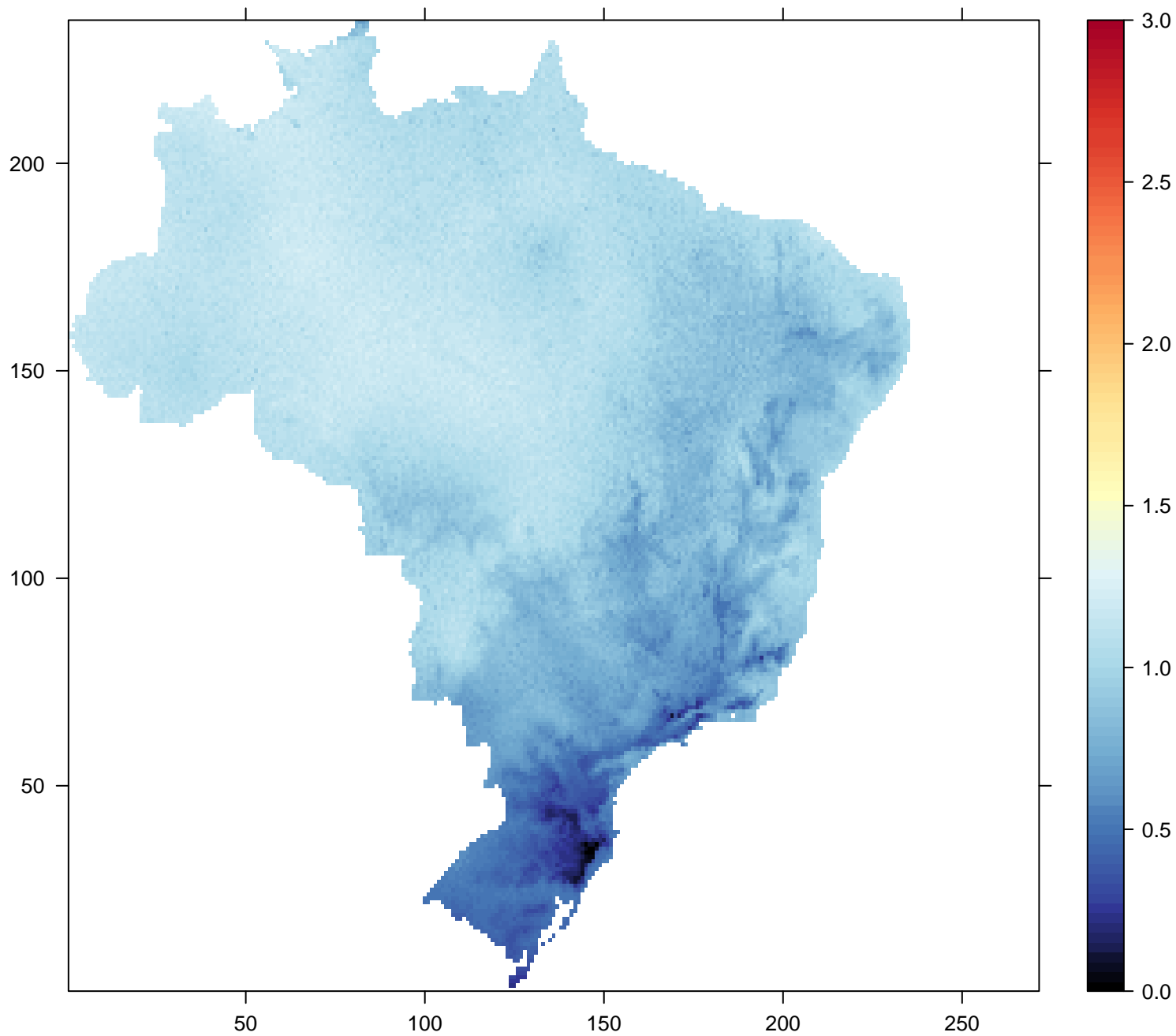

OCTOBER

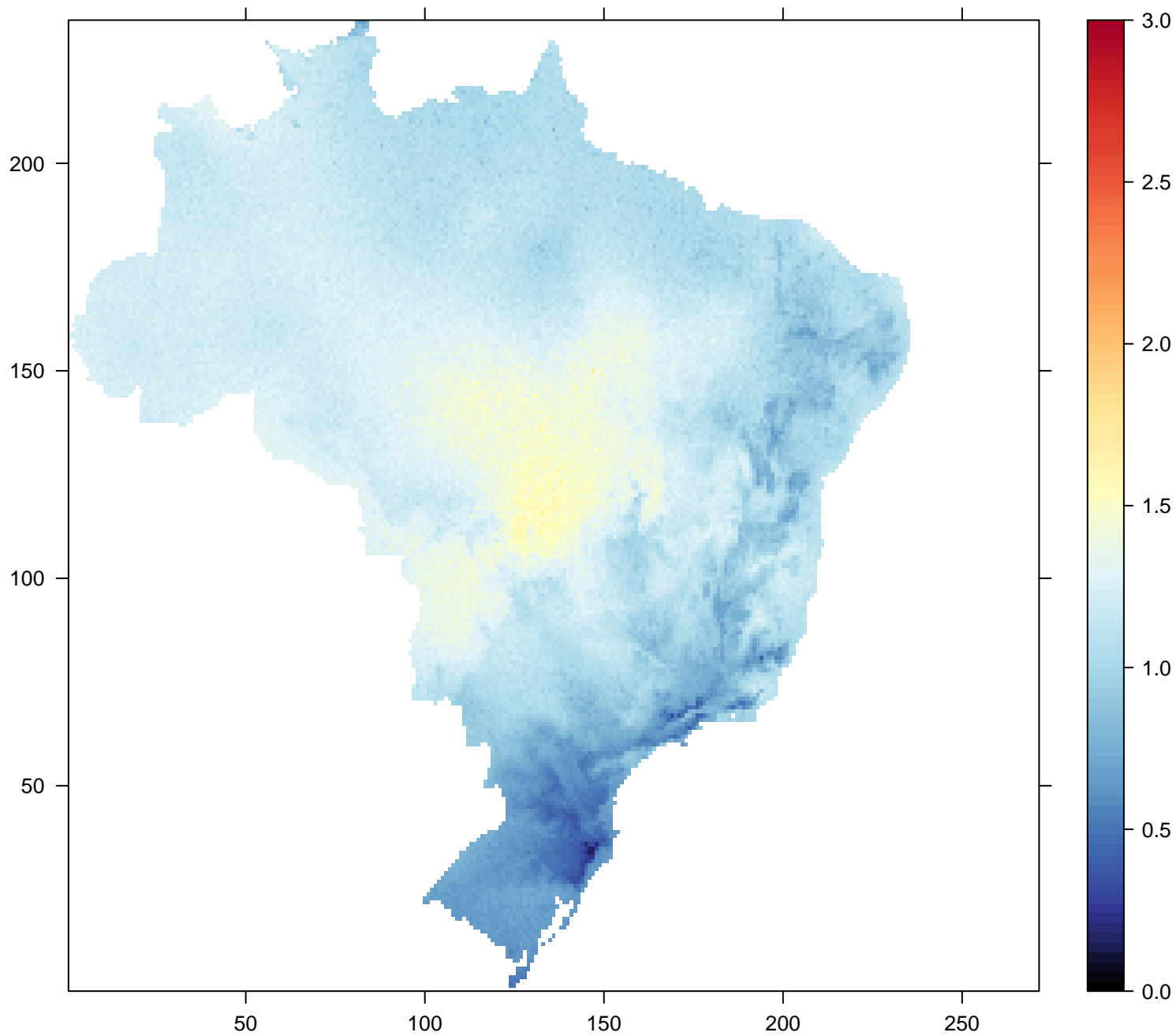

# NOVEMBER

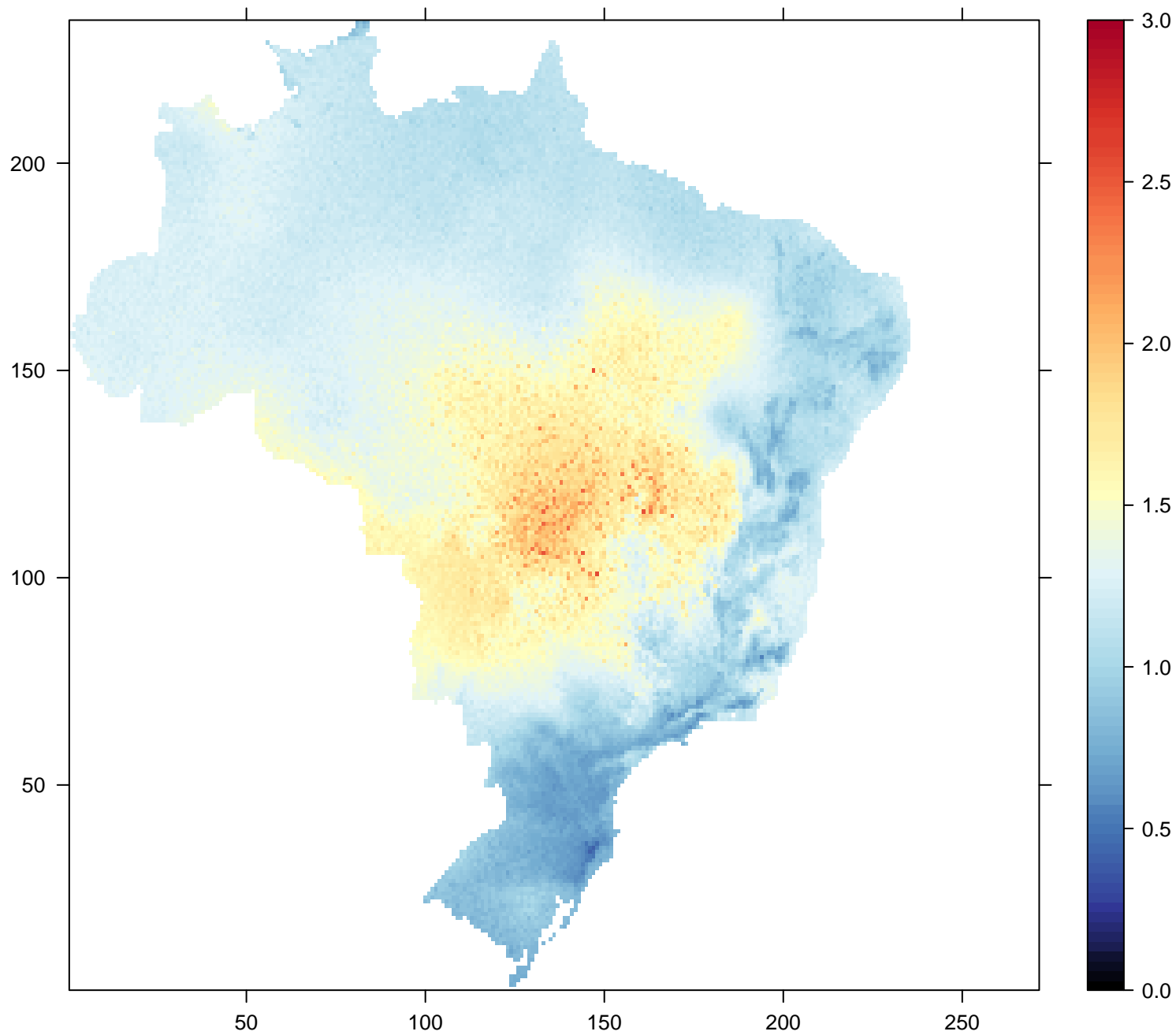

# DECEMBER

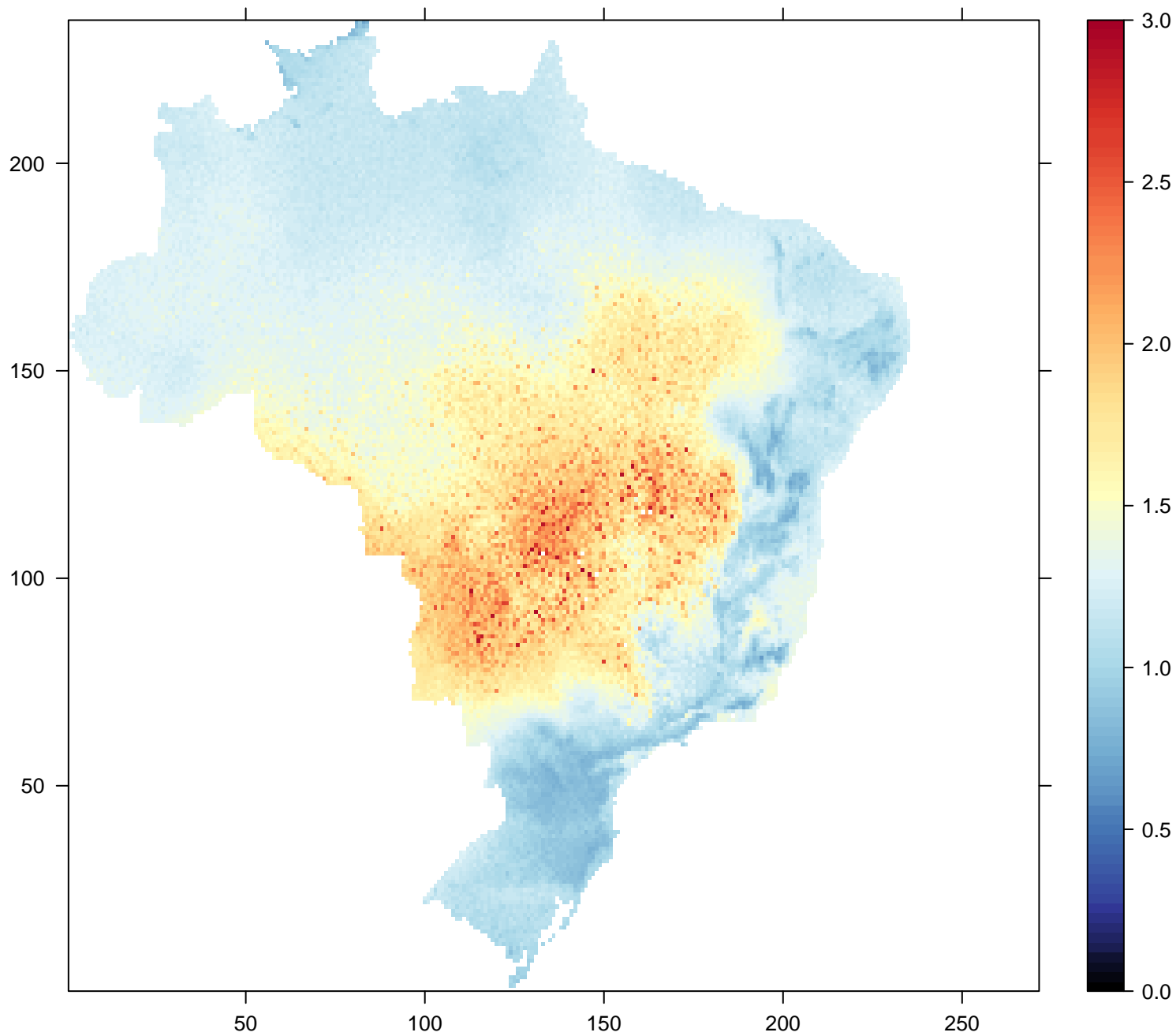

Supplement: Supplementary file 1 [file MEE3-10-1357-s001.zip › mee313205-sup-0002-SpatioTemporalFramesS2.pdf]
